# A quest for the stereo-electronic requirements for selective agonism for the neurotrophin receptors TrkA and TrkB in 17-spirocyclic-dehydroepiandrosterone derivatives

**DOI:** 10.3389/fnmol.2023.1244133

**Published:** 2023-09-28

**Authors:** Daniele Narducci, Despoina Charou, Thanasis Rogdakis, Ioanna Zota, Vivi Bafiti, Maria Zervou, Theodora Katsila, Achille Gravanis, Kyriakos C. Prousis, Ioannis Charalampopoulos, Theodora Calogeropoulou

**Affiliations:** ^1^Institute of Chemical Biology, National Hellenic Research Foundation, Athens, Greece; ^2^Institute of Molecular Biology and Biotechnology, Foundation for Research and Technology Hellas, Heraklion, Greece; ^3^Department of Pharmacology, Medical School, University of Crete, Heraklion, Greece

**Keywords:** neurotrophin mimetics, TrkA, TrkB, receptor agonists, signaling, neuroprotection, neurodegeneration, DHEA derivatives

## Abstract

**Introduction:**

The neurotrophin system plays a pivotal role in the development, morphology, and survival of the nervous system, and its dysregulation has been manifested in numerous neurodegenerative and neuroinflammatory diseases. Neurotrophins NGF and BDNF are major growth factors that prevent neuronal death and synaptic loss through binding with high affinity to their specific tropomyosin-related kinase receptors namely, TrkA and TrkB, respectively. The poor pharmacokinetic properties prohibit the use of neurotrophins as therapeutic agents. Our group has previously synthesized BNN27, a prototype small molecule based on dehydroepiandrosterone, mimicking NGF through the activation of the TrkA receptor.

**Methods:**

To obtain a better understanding of the stereo-electronic requirements for selective activation of TrkA and TrkB receptors, 27 new dehydroepiandrosterone derivatives bearing a C17-spiro-dihydropyran or cyclobutyl moiety were synthesized. The new compounds were evaluated for their ability (a) to selectively activate the TrkA receptor and its downstream signaling kinases Akt and Erk1/2 in PC12 cells, protecting these cells from serum deprivation-induced cell death, and (b) to induce phosphorylation of TrkB and to promote cell survival under serum deprivation conditions in NIH3T3 cells stable transfected with the TrkB receptor and primary cortical astrocytes. In addition the metabolic stability and CYP-mediated reaction was assessed.

**Results:**

Among the novel derivatives, six were able to selectively protect PC12 cells through interaction with the TrkA receptor and five more to selectively protect TrkB-expressing cells via interaction with the TrkB receptor. In particular, compound ENT-A025 strongly induces TrkA and Erk1/2 phosphorylation, comparable to NGF, and can protect PC12 cells against serum deprivation-induced cell death. Furthermore, ENT-A065, ENT-A066, ENT-A068, ENT-A069, and ENT-A070 showed promising pro-survival effects in the PC12 cell line. Concerning TrkB agonists, ENT-A009 and ENT-A055 were able to induce phosphorylation of TrkB and reduce cell death levels in NIH3T3-TrkB cells. In addition, ENT-A076, ENT-A087, and ENT-A088 possessed antiapoptotic activity in NIH-3T3-TrkB cells exclusively mediated through the TrkB receptor. The metabolic stability and CYP-mediated reaction phenotyping of the potent analogs did not reveal any major liabilities.

**Discussion:**

We have identified small molecule selective agonists of TrkA and TrkB receptors as promising lead neurotrophin mimetics for the development of potential therapeutics against neurodegenerative conditions.

## 1. Introduction

Traced back to over 600 million years ago (Hallböök et al., 2006), the neurotrophin (NT) system plays a pivotal role in the development, morphology, and survival of the nervous system (Shahnaz et al., 2015). In addition, it influences a wide range of functions, among which the most studied are related to memory (Mitre et al., 2016), the perception of fear (Penzo et al., 2015), pain (Allen and Dawbarn, 2006), hunger (Ceren et al., 2015), and taste (Fei and Krimm, 2013), and even insulin secretion and receptivity (Baeza-Raja et al., 2012; Houtz et al., 2016).

Over the years, various secreted growth factors belonging to the neurotrophins family were discovered the most important being the nerve growth factor (NGF), the brain-derived neurotrophic factor (BDNF), and neurotrophin 3 (NT-3). To produce their beneficial effects, neurotrophins bind with high affinity to a specific tropomyosin receptor kinase (Trk), namely, NGF and NT-3 (with less affinity than NGF) to TrkA, BDNF to TrkB, and NT-3 to TrkC. The last member of the neurotrophin system is the 75 kDa pan-neurotrophin receptor (p75^NTR^), bound with low affinity by all neurotrophins and with high affinity by their precursors: the proneurotrophins (pro-NTs) (Chao et al., 2006; Mitre et al., 2016). The correct functioning of the neurotrophin system was famously schematized by Bai et al. (2005) with a Yin and Yang representation, to remark how the homeostasis relies on a finely regulated balance between NT/Trk-stimulated survival and pro-NT/p75-stimulated apoptosis. Dysregulations in the aforementioned equilibrium of the neurotrophin system were observed in numerous neurodegenerative and neuroinflammatory diseases. Among the most famous, Alzheimer's disease (AD), Parkinson's disease (PD), Huntington's disease (HD), amyotrophic lateral sclerosis (ALS), and multiple sclerosis (MS) are highlighted (Meldolesi, 2017).

Following this general understanding, activation of the Trks holds the potential to revolutionize the treatment of neurodegeneration from being merely palliative to disease-modifying. Alas, despite numerous past and ongoing attempts, neurotrophins themselves are proving to be an unviable therapeutic modality because of the typical pharmacokinetic issues that characterize peptide-based medications. These include short half-life, poor oral bioavailability, and low ability to cross the blood–brain barrier (Pardridge, 2002), accompanied by topical hyperalgesia, undesirably high spike-like potency, and counterproductive p75^NTR^ activation (Josephy-Hernandez et al., 2017).

A variety of strategies have been conceived to target the neurotrophin receptors and were recently reviewed (Saragovi et al., 2019; Gudasheva et al., 2021; Jeyaram et al., 2023; Rahman et al., 2023). The current study focuses specifically on new small molecule neurotrophin mimetics capable of achieving selective Trk receptor activation circumventing the limitations associated with the direct use of neurotrophins in therapy, thus paving the way for more effective and tailored neuroprotection strategies. In this context, the endogenous neurosteroid dehydroepiandrosterone (DHEA) acts as the stepstone of our study as it can bind to all the neurotrophin receptors stimulating neurogenesis, neuronal survival, and neurite outgrowth and reduce neuroinflammation (Baulieu, 1996; Lazaridis et al., 2011; Pediaditakis et al., 2015; Alexaki et al., 2018; Yilmaz et al., 2019).

Capitalizing on these beneficial properties, Calogeropoulou et al. (2009) designed and synthesized a series of modified DHEA derivatives aiming to enhance the potency and selectivity of the neurotrophin receptors while hindering metabolism into androgens and estrogens. Among these, the C17-spiro-epoxy analog BNN27 emerged as the lead compound. BNN27 is a selective TrkA and p75NTR agonist (Pediaditakis et al., 2016a,b) and can exert neuroprotective and anti-inflammatory activity in several *in vitro* and *in vivo* studies (Glajch et al., 2016; Bonetto et al., 2017; Pitsikas and Gravanis, 2017; Ibán-Arias et al., 2018; Tsika et al., 2021). Furthermore, BNN27 does not bind to hormonal receptors and is not metabolically converted to estrogens or androgens (Calogeropoulou et al., 2009; Bennett et al., 2016). However, it was shown that BNN27 undergoes rapid hydroxylation and subsequent excretion during hepatic catabolism, resulting in an elimination constant (K_el_) of 0.465 h^−1^ and a mean residence time (MRT) of 2.154 h in murine models (Bennett et al., 2016; Tsika et al., 2021). To address the metabolic stability issue of BNN27 while maintaining potency and enhancing selectivity toward TrkA or TrkB receptors, our initial research efforts sought to replace the C-17 spiro epoxide moiety with the more stable cyclopropyl group with promising results (Rogdakis et al., 2022; Yilmaz et al., 2022).

**Figure 1:**
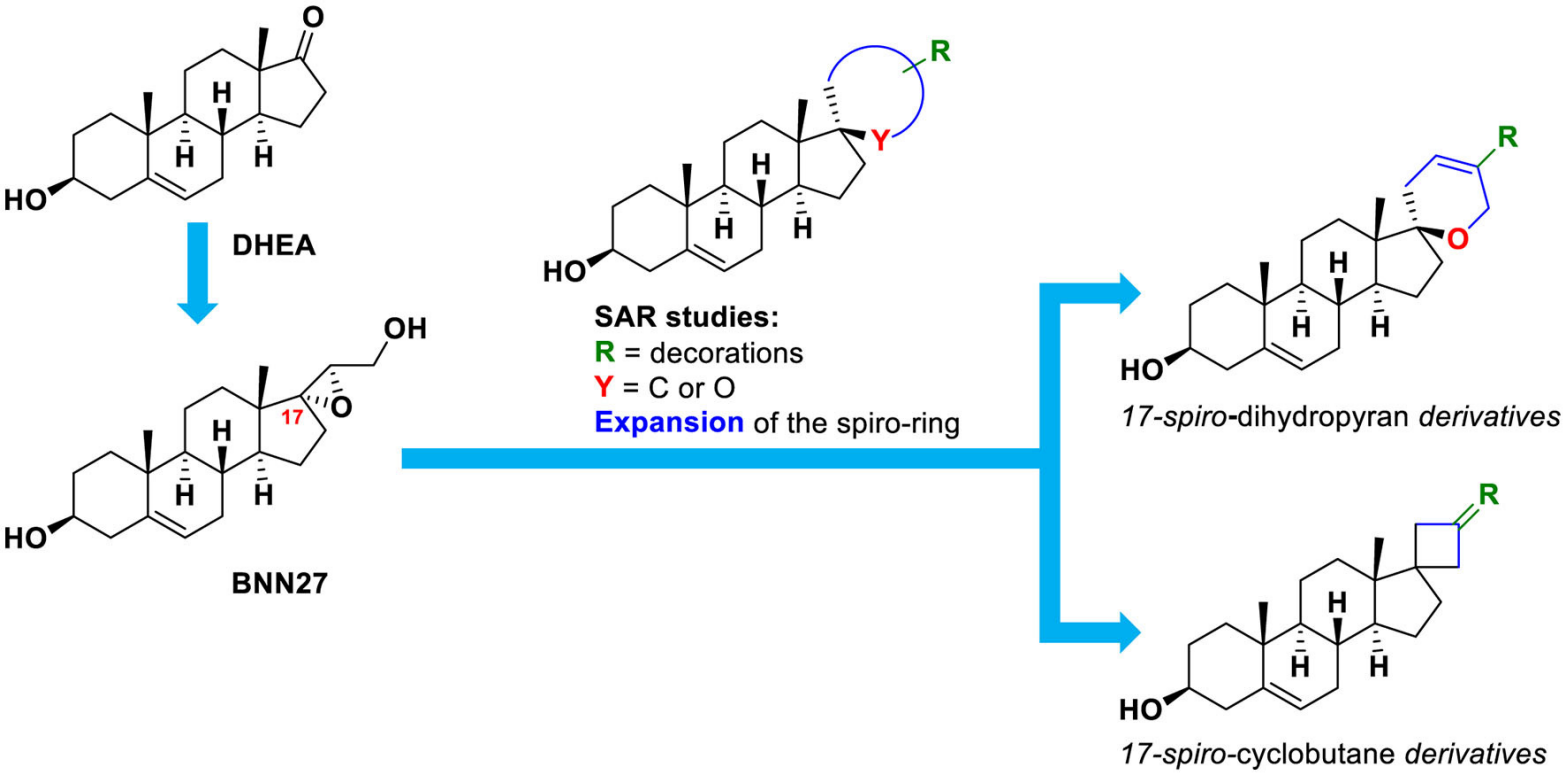
Conceptual representation of the design of the compounds of the present study.

In the current study, we set out to investigate the presence of larger spirocyclic moieties, in particular, cyclobutyl and dihydropyran rings, attached to DHEA at the C17 position (Figure 1). In turn, these rings were decorated with carefully selected substituents to enrich the structure–activity relationships for neurotrophin mimetic activity. Additionally, we synthesized one derivative featuring a C17-spiro-tetrahydropyran ring and one with a C17-spiro-oxepan-3-one moiety.

## 2. Materials and methods

### 2.1. Chemistry

#### 2.1.1. (3*S*,17*R*)-3′,6′-dihydrospiro[5-androstene-17,2′-pyran]-3-ol (ENT-A002)

A solution of tetrabutylammonium fluoride (TBAF) (1.0 M in THF, 1.3 mL, 1.3 mmol) was added to a solution of compound **4** (102 mg, 0.18 mmol) in anhydrous tetrahydrofuran (2 mL), and the reaction was stirred at 25°C for 48 h. The reaction mixture was diluted with dichloromethane and the organic phase was washed with water and brine, dried over Na_2_SO_4_, and the solvent was removed *in vacuo*. The residue was purified by FCC (elution solvent: petroleum ether 40–60°C/diethyl ether: 9/1) to afford **ENT-A002** as a white crystalline solid (52 mg, 83% yield). **Rf:** 0.08 (petroleum ether 40–60°C/diethyl ether: 8/2); **mp:** 119–121°C; ^**1**^**H NMR (600 MHz, CDCl**_**3**_**):** δ 5.75–5.74 (m, 1H), 5.69–5.67 (m, 1H), 5.33 (d, *J* = 4.2 Hz, 1H), 4.21–4.14 (m, 2H), 3.52–3.47 (m, 1H), 2.36 (d, *J* = 16.9 Hz, 1H), 2.28 (dd, *J* = 3.0, 12.7 Hz, 1H), 2.23–2.20 (m, 1H), 2.00–1.05 (m, 18H), 1.01 (s, 3H), 0.87 (s, 3H); ^**13**^**C NMR (151 MHz, CDCl**_**3**_**):** δ 140.8, 125.8, 123.3, 121.3, 83.0, 71.6, 63.2, 51.0, 50.0, 45.8, 42.2, 37.2, 36.5, 33.2, 32.9, 32.4, 31.7, 31.5, 31.2, 23.3, 20.8, 19.3, 13.6; **HR-MS (APCI**^**+**^**):** calcd for C_23_H_35_O_2_ [M+H]^+^ 343.2632, found 343.2623; calcd for C_23_H_33_O [M-H_2_O+H]^+^ 325.2526, found 325.2520.

#### 2.1.2. (3*S*,17*R*)-5′-(chloromethyl)-3′,6′-dihydrospiro[5-androstene-17,2′-pyran]-3-ol (ENT-A025)

HF·pyridine complex (1.3 mL, 14.43 mmol) was added to a solution of compound **6** (0.505 g, 1.00 mmol) in anhydrous dichloromethane (33.0 mL) at 0°C, and the reaction was stirred at the same temperature for 40 min. The reaction was quenched with water and extracted with dichloromethane. The organic layer was washed with brine, dried over Na_2_SO_4_, and concentrated *in vacuo*. The residue was purified by FCC (ethyl acetate/petroleum ether 35-60°C: 4/6) to afford **ENT-A025**, as a white solid (0.39 g, yield quantitative). **Rf:** 0.30 (ethyl acetate/petroleum ether 35-60°C: 4/6); **mp:** 128–130°C; ${\left[a\right]}_{D}^{24}=-$ 46.05° (*c* = 0.00160 g/mL, CHCl_3_); ^**1**^**H NMR (600 MHz, CDCl**_**3**_**):** δ 5.88 (bs, 1H), 5.36–5.33 (m, 1H), 4.25 (ABq, *J*_*AB*_ = 16.4 Hz, 2H), 3.99 (s, 2H), 3.55–3.48 (m, 1H), 2.43–0.86 (m, 22H), 1.02 (s, 3H), 0.92 (s, 3H); ^**13**^**C NMR (75 MHz, CDCl**_**3**_**):** δ 141.0, 133.0, 124.4, 121.5, 83.2, 71.9, 63.8, 51.5, 50.2, 45.9, 45.8, 42.4, 37.4, 36.7, 33.7, 33.3, 32.7, 31.8, 31.5, 29.8, 23.4, 21.0, 19.5, 13.7. **HR-MS (APCI**^**+**^**):** m/z calculated for C_24_H_36_^35^ClO_2_ 391.2398 [M+H]^+^, found 391.2396; **HPLC:** 100% purity, RT = 27.42 min. Column: Kromasil 100-10-SIL (10 × 250 mm, 10 μm), Method: eluting with 20% ethyl acetate −80% toluene, isocratic, flow rate 1.5 mL/min at 25°C, and injection volume 20 μL.

#### 2.1.3. (3*S*,17*R*)-3-hydroxy-3′,6′-dihydrospiro[5-androstene-17,2′-pyran]-5′-yl-acetonitrile (ENT-A033)

A solution of **ENT-A025** (20.0 mg, 0.06 mmol) and KCN (10.6 mg, 0.16 mmol) in a mixture of EtOH/water/dichloromethane: 2/1/1(1.6 mL) was vigorously stirred at 25°C for 12 h. Subsequently, the reaction was stirred at 40°C for 5 h, and then KCN (10.6 mg, 0.16 mmol) was added and the reaction was stirred at 25°C for an additional 12 h. The reaction mixture was evaporated under reduced pressure, and the residue was partitioned between aqueous saturated sodium thiosulphate solution and dichloromethane. The organic layer was washed with brine, dried over Na_2_SO_4_, and concentrated *in vacuo*. The residue was purified by FCC (elution solvent: dichloromethane/MeOH: 99/1) to afford **ENT-A033**, as a white solid (16 mg, 70% yield). **Rf:** 0.50 (dichloromethane/MeOH: 96/4); **mp:** 173–175°C; ${\left[a\right]}_{D}^{24}=+46.88^{•}$(*c* = 0.00128 g/mL, CHCl_3_); ^**1**^**H NMR (600 MHz, CDCl**_**3**_**):** δ 5.91–5.89 (m, 1H), 5.37–5.33 (m, 1H), 4.13 and 4.10 (two d, ABq, *J*_*AB*_ = 17.6 Hz, 2H), 3.56–3.48 (m, 1H), 2.98 (s, 2H), 2.43–0.83 (m, 21H), 1.02 (s, 3H), 0.91 (s, 3H). ^**13**^**C NMR (151 MHz, CDCl**_**3**_**):** δ 141.0, 125.7, 123.3, 121.5, 116.8, 83.4, 71.9, 64.7, 51.4, 50.2, 45.9, 42.4, 37.4, 33.4, 33.2, 32.7, 31.8, 31.8, 31.3, 29.8, 23.5, 21.4, 21.0, 19.6, 13.7; **HR-MS (APCI**^**+**^**):** m/z calculated for C_25_H_36_NO_2_ 382.2741 [M+H]^+^, found 382.2737; **HPLC:** 100% purity, RT = 36.47 min. Column: Kromasil 100-10-SIL (10 × 250 mm, 10 μm), Method: eluting with 20% ethyl acetate-−80% toluene, isocratic, flow rate 1.5 mL/min at 25°C, and injection volume 20 μL.

#### 2.1.4. (3*S*,17*R*)-5′-((diethylamino)methyl)-3′,6′-dihydrospiro[5-androstene-17,2′-pyran]-3-ol (ENT-A075)

Diethylamine (14.5 μL, 0.14 mmol) was added to a solution of **ENT-A025** (27.3 mg, 0.07 mmol) in anhydrous dichloromethane (0.4 mL), and the reaction was stirred at 25°C for 48 h. To the reaction mixture, saturated aqueous ammonium chloride solution was added and extracted with dichloromethane. The organic phase was washed with brine, dried over anhydrous Na_2_SO_4_, and concentrated *in vacuo*. The residue was purified by FCC (elution solvent: dichloromethane/MeOH: 9/1) to afford **ENT-A076** as a white solid (27 mg, 90% yield). **Rf:** 0.21 (dichloromethane/MeOH: 9/1); **mp:** 144°C, decomposition; ${\left[a\right]}_{D}^{24}=\;-$ 46.15° (*c* = 0.00130 g/mL, CHCl_3_); ^**1**^**H NMR (600 MHz, CDCl**_**3**_**):** δ 5.67 (bs, 1H), 5.36–5.32 (m, 1H), 4.18 and 4.15 (two d, ABq, *J*_*AB*_ = 17.3 Hz, 2H), 3.55–3.47 (m, 1H), 2.97 (s, 2H), 2.55–2.49 (m, 4H), 2.39–0.86 (m, 25H), 1.02 (s, 3H), 0.90 (s, 3H); ^**13**^**C NMR (75 MHz, CDCl**_**3**_**):** δ 141.0, 134.1, 121.5, 121.5, 83.3, 71.9, 65.4, 56.6, 51.4, 50.3, 46.5, 45.9, 42.4, 37.5, 36.7, 33.5, 33.2, 32.7, 31.9, 31.8, 31.5, 23.5, 21.1, 19.6, 13.8, 11.3. **HR-MS (APCI**^**+**^**):** m/z calculated for C_28_H_46_O_2_N 428.3523 [M+H]^+^, found 428.3521; **HPLC:** 100% purity, RT = 14.23 min. Column: Kromasil 100-10-SIL (10 × 250 mm, 10 μm), Method: eluting with 15% chloroform-−85% acetone, isocratic, flow rate 2.1 mL/min at 25°C, and injection volume 20 μL.

#### 2.1.5. (3*S*,17*R*)-5′-[(4-(hydroxymethyl)-1H-1,2,3-triazol-1-yl)methyl]-3′,6′-dihydrospiro[5-androstene-17,2′-pyran]-3-ol (ENT-A037)

A mixture of azide **9** (55.7 mg, 0.14 mmol), propargyl alcohol (34.0 μL, 0.42 mmol), CuSO_4_·5H_2_O (10.4 mg, 0.04 mmol), and sodium ascorbate (16.6 mg, 0.08 mmol) in dichloromethane/water/^t^BuOH (1/1/1, 3.0 mL) was stirred at 25°C for 12 h. Subsequently, the reaction mixture was extracted with dichloromethane and the organic layer was washed with brine, dried over anhydrous Na_2_SO_4_, and concentrated under reduced pressure. The residue was purified by FCC (elution solvent: dichloromethane/MeOH: 96/4) to afford **ENT-A037**, as a white solid (31 mg, 49%). **Rf:** 0.28 (dichloromethane/MeOH: 95/5); **mp:** 202–204°C; ${\left[a\right]}_{D}^{24}=+41.38^{•}$(*c* = 0.00145 g/mL, CHCl_3_); ^**1**^**H NMR (300 MHz, CD**_**3**_**OD):** δ 7.87 (s, 1H), 5.88 (bs, 1H), 5.38–5.30 (m, 1H), 4.93 (bs, 2H), 4.67 (s, 2H), 4.04 (bs, 2H), 3.45–3.34 (m, 1H), 2.42 (d, *J* = 17.3 Hz, 1H), 2.26–0.86 (m, 20H), 1.03 (s, 3H), 0.88 (s, 3H); ^**13**^**C NMR (75 MHz, CD**_**3**_**OD):** 149.4, 142.3, 132.6, 125.5, 124.1, 122.2, 84.5, 72.4, 64.4, 56.5, 53.3, 52.5, 51.6, 47.0, 43.0, 38.6, 37.7, 34.4, 34.2, 33.9, 32.8, 32.3, 32.2, 24.3, 22.0, 19.8, 14.0; **HR-MS (APCI**^**+**^**):** m/z calculated for C_27_H_40_N_3_O_3_ 454.3064 [M+H]^+^, found 454.3063; **HPLC:** 100% purity, RT = 41.36 min. Column: Agilent Eclipse XDB-C8 (4.6 × 150 mm, 5 μm), Method: eluting with 10% water-−90% methanol, isocratic, flow rate 1.0 mL/min at 10°C, and injection volume 20 μL.

#### 2.1.6. (3*S*,17*R*)-5′-[(4-((dimethylamino)methyl)-1H-1,2,3-triazol-1-yl)methyl]-3′,6′-dihydrospiro[5-androstene-17,2′-pyran]-3-ol (ENT-A046)

A mixture of azide **9** (13.7 mg, 0.04 mmol), *N,N*-dimethyl-propargyl amine (12.0 μL, 0.11 mmol), CuSO_4_·5H_2_O (2.6 mg, 0.01 mmol), and sodium ascorbate (4.2 mg, 0.02 mmol) in dichloromethane/water/^t^BuOH (1/1/1, 1.0 mL) was stirred at 25°C for 12 h. Subsequently, the reaction mixture was extracted with dichloromethane and the organic layer was washed with brine, dried over anhydrous Na_2_SO_4_, and concentrated under reduced pressure. The residue was purified by FCC (elution solvent: dichloromethane/MeOH: 93/7) to afford **ENT-A046**, as a white solid (17 mg, quantitative yield). **Rf:** 0.37 (dichloromethane/MeOH: 9/1); **mp:** 182°C, decomposition; ${\left[a\right]}_{D}^{24}=-31.50^{•}$(*c* = 0.00127 g/mL, CHCl_3_); ^**1**^**H NMR (300 MHz, CDCl**_**3**_**):** δ 7.60 (s, 1H), 5.83 (bs, 1H), 5.37–5.30 (m, 1H), 4.85 (s, 2H), 4.02 (bs, 2H), 3.70 (bs, 2H), 3.59–3.44 (m, 1H), 2.43–0.83 (m, 24H), 1.01 (s, 3H), 0.86 (s, 3H); ^**13**^**C NMR (75 MHz, CDCl**_**3**_**):** δ 144.5, 141.0, 131.1, 124.9, 122.9, 121.4, 83.3, 71.8, 63.5, 54.2, 52.9, 51.4, 50.2, 45.9, 44.8, 42.4, 37.4, 36.7, 33.3, 33.2, 32.6, 31.8, 31.3, 29.8, 23.4, 21.0, 19.5, 13.7. **HR-MS (APCI**^**+**^**):** m/z calculated for C_29_H_45_N_4_O_3_ 481.3537 [M+H]^+^, found 481.3541; **HPLC:** 100% purity, RT = 29.7 min. Column: Fortis C18 (10 × 250 mm, 10 μm), Method: eluting with 8% water−92% methanol, isocratic, flow rate 1.0 mL/min at 25°C, and injection volume 20 μL.

#### 2.1.7. (3*S*,17*R*)-5′-[(4-(cyclohexylmethyl)-1H-1,2,3-triazol-1-yl)methyl]-3′,6′-dihydrospiro[5-androstene-17,2′-pyran]-3-ol (ENT-A047)

A mixture of azide **9** (24.0 mg, 0.06 mmol), prop-2-yn-1-yl-cyclohexane (26.0 μL, 0.18 mmol), CuSO_4_·5H_2_O (4.5 mg, 0.02 mmol), and sodium ascorbate (7.1 mg, 0.04 mmol) in dichloromethane/water/^t^BuOH (1/1/1, 1.0 mL) was stirred at 25°C for 12 h. Subsequently, the reaction mixture was extracted with dichloromethane and the organic layer was washed with brine, dried over anhydrous Na_2_SO_4_, and concentrated under reduced pressure. The residue was purified by FCC (elution solvent: hexane/ethyl acetate: 6/4) to afford **ENT-A047**, as a white solid (19 mg, 61% yield). **Rf:** 0.22 (petroleum ether 35–60°C/ethyl acetate: 1/1); **mp:** 181°C, decomposition; ${\left[a\right]}_{D}^{24}=-72.99\;$(*c* = 0.00137 g/mL, CHCl_3_); ^**1**^**H NMR (600 MHz, CDCl**_**3**_**):** δ 7.22 (s, 1H), 5.82 (bs, 1H), 5.36–5.32 (m, 1H), 4.82 (s, 2H), 4.03 and 3.99 (two d, ABq, *J*_*AB*_ = 17.8 Hz, 2H), 3.55–3.47 (m, 1H), 2.57 (d, *J* = 7.0 Hz, 2H), 2.44–0.83 (m, 33H), 1.01 (s, 3H), 0.87 (s, 3H). ^**13**^**C NMR (151 MHz, CDCl**_**3**_**):** δ 140.8, 131.2, 124.4, 121.3, 120.8, 83.1, 71.7, 63.5, 52.6, 51.3, 50.1, 45.8, 42.2, 38.0, 37.3, 36.5, 33.4, 33.4, 33.1, 33.0, 33.0, 32.5, 31.7, 31.6, 31.2, 29.7, 26.4, 26.1, 23.3, 20.8, 19.4, 13.5. **HR-MS (APCI**^**+**^**):** m/z calculated for C_33_H_50_N_3_O_2_ 520.3898 [M+H]^+^, found 520.3898; **HPLC:** 100% purity, RT = 32.74 min. Column: Kromasil 100-10-SIL (10 × 250 mm, 10 μm), Method: eluting with 35% ethyl acetate −65% toluene, isocratic, flow rate 2.1 mL/min at 25°C, and injection volume 20 μL.

#### 2.1.8. (3*S*,17*R*)-5′-[(4-benzyl-1H-1,2,3-triazol-1-yl)methyl]-3′,6′-dihydrospiro[5-androstene-17,2′-pyran]-3-ol (ENT-A055)

A mixture of azide **9** (24.0 mg, 0.06 mmol), 3-phenyl-1-propyne (22.0 μL, 0.18 mmol), CuSO_4_·5H_2_O (4.5 mg, 0.02 mmol), and sodium ascorbate (7.1 mg, 0.04 mmol) in dichloromethane/water/^t^BuOH (1/1/1, 1.0 mL) was stirred at 25°C for 12 h. Subsequently, the reaction mixture was extracted with dichloromethane and the organic layer was washed with brine, dried over anhydrous Na_2_SO_4_, and concentrated under reduced pressure. The residue was purified by FCC (elution solvent: hexane/ethyl acetate: 7/3) to afford **ENT-A055**, as a white solid (22 mg, 71% yield). **Rf:** 0.51 (petroleum ether 35–60°C/ethyl acetate: 4/6); **mp:** 201–203°C; ${\left[a\right]}_{D}^{24}=-96.00^{•}$(*c* = 0.00125 g/mL, CHCl_3_); ^**1**^**H NMR (600 MHz, CDCl**_**3**_**):** δ 7.34–7.22 (m, 5H), 7.14 (s, 1H), 5.81 (bs, 1H), 5.37–5.33 (m, 1H), 4.81 (s, 2H), 4.82 and 4.80 (two d, ABq, *J*_*AB*_ = 15.4 Hz, 2H), 4.11 and 4.08 (two d, ABq, *J*_*AB*_ = 16.6 Hz, 2H), 4.02 and 3.99 (two d, ABq, *J*_*AB*_ = 17.5 Hz, 2H), 3.57–3.49 (m, 1H), 2.43–0.85 (m, 21H), 1.03 (s, 3H), 0.87 (s, 3H); ^**13**^**C NMR (151 MHz, CDCl**_**3**_**):** δ 148.2, 141.0, 139.1, 131.2, 128.8, 128.8, 126.6, 124.7, 121.4, 121.2, 83.3, 71.8, 63.6, 52.9, 51.4, 50.2, 45.9, 42.4, 37.4, 36.7, 33.5, 33.2, 32.6, 32.4, 31.8, 31.8, 31.3, 23.4, 21.0, 19.5, 13.6. **HR-MS (APCI**^**+**^**):** m/z calculated for C_33_H_44_N_3_O_2_ 514.3428 [M+H]^+^, found 514.3432; **HPLC:** 100% purity, RT = 24.79 min. Column: Kromasil 100-10-SIL (10 × 250 mm, 10 μm), Method: eluting with 35% ethyl acetate-−65% toluene, isocratic, flow rate 2.1 mL/min at 25°C, and injection volume 20 μL.

#### 2.1.9. Ethyl (E)-(3*S*,17*R*)-3-hydroxy-dihydrospiro[5-androstene-17,2′-pyran]-5′-yl-acrylate (ENT-A007)

**Step 1**. Triethyl phosphonoacetate (0.11 mL, 0.52 mmol) was added to a suspension of NaH (20.8 mg, 0.52 mmol) in anhydrous tetrahydrofuran (0.4 mL) at 0°C, and the reaction was stirred at 25°C for 30 min. The reaction mixture was cooled to 0°C and a solution of aldehyde **10** (64.0 mg, 0.13 mmol) in anhydrous tetrahydrofuran (1.6 mL) was added and the mixture was stirred at the same temperature for 1 h. The reaction was quenched, at the same temperature, by the dropwise addition of brine and extracted with ethyl acetate. The organic layer was dried over anhydrous Na_2_SO_4_ and concentrated *in vacuo*. The product was used without further purification in the next step (32 mg, 54% yield). **Rf:** 0.13 (petroleum ether 35–60°C/ethyl acetate: 85/15).

**Step 2**. HF·pyridine complex (0.2 mL, 2.22 mmol) was added dropwise to a solution of the crude product from step 1 (32 mg, 0.06 mmol) in anhydrous dichloromethane (2.4 mL) at 0°C, and the reaction was stirred at 0°C for 40 min. The reaction was quenched at 0°C with water and extracted with dichloromethane. The organic layer was washed with brine, dried over anhydrous Na_2_SO_4_, and concentrated under reduced pressure. The residue was purified by FCC (elution solvent: petroleum ether 35–60°C/ethyl acetate: 85/15) to afford **ENT-A007**, as a white solid (20.0 mg, 35% yield over two steps). **Rf:** 0.47 (petroleum ether 35–60°C/ethyl acetate: 6/4); **mp:** 161−163°C; ${\left[a\right]}_{D}^{24}=-59.26^{•}$(*c* = 0.00135 g/mL, CHCl_3_); ^**1**^**H NMR (600 MHz, CDCl**_**3**_**):** δ 7.20 (d, *J* = 16.2 Hz, 1H), 6.22 (bs, 1H), 5.58 (d, *J* = 16.2 Hz, 1H), 5.38–5.30 (m, 1H), 4.38 and 4.30 (two d, ABq, *J*_*AB*_ = 16.5 Hz, 2H), 4.20 (q, *J* = 7.1 Hz, 2H), 3.60–3.43 (m, 1H), 2.54 (d, *J* = 18.5 Hz, 1H), 2.32–0.82 (m, 20H), 1.30 (t, *J* = 6.9 Hz, 3H), 1.01 (s, 3H), 0.92 (s, 3H). ^**13**^**C NMR (75 MHz, CDCl**_**3**_**):** δ δ 167.3, 143.2, 141.0, 134.5, 133.2, 121.4, 115.1, 83.5, 71.8, 62.6, 60.5, 51.5, 50.1, 42.3, 37.4, 36.7, 33.8, 33.3, 32.7, 32.6, 31.8, 31.7, 29.8, 23.4, 21.0, 19.5, 14.4, 13.6; **HR-MS (APCI**^**−**^**):** m/z calculated for C_28_H_39_O_4_ 439.2854 [M-H]^−^, found m/z 439.2848; **HPLC:** 100% purity, RT = 29.99 min. Column: Kromasil 100-10-SIL (10 × 250 mm, 10 μm), Method: eluting with 20% ethyl acetate −80% toluene, isocratic, flow rate 1.5 mL/min at 25°C, and injection volume 20 μL.

#### 2.1.10. (E)-(3*S*,17*R*)-3-hydroxy-3′,6′-dihydrospiro[5-androstene-17,2′-pyran]-5′-acrylonitrile (ENT-A009)

**Step 1**. Diethyl (cyanomethyl) phosphonate (30.0 μL, 0.28 mmol) was added to a suspension of NaH (11.2 mg, 0.28 mmol) in anhydrous tetrahydrofuran (0.2 mL) at 0°C, and the reaction was stirred at 25°C for 30 min. The reaction was cooled to 0°C and a solution of aldehyde **10** (34.0 mg, 0.07 mmol) in anhydrous tetrahydrofuran (0.7 mL) was added and the mixture was stirred at the same temperature for 1 h. The reaction was quenched, at the same temperature, by the dropwise addition of brine and extracted with ethyl acetate. The organic layer was dried over anhydrous Na_2_SO_4_ and concentrated *in vacuo*. The product was used without further purification in the next step (27 mg, 71% yield). **Rf:** 0.13 (petroleum ether 35–60°C/Et_2_O: 94/6).

**Step 2**. HF·pyridine complex (60.0 μL, 0.67 mmol) was added dropwise to a solution of the crude product from step 1 (27.0 mg, 0.05 mmol) in anhydrous dichloromethane (1.7 mL) at 0°Cof, and the reaction was stirred at 0°C for 40 min. The reaction was quenched at 0°C with water and extracted with dichloromethane. The organic layer was washed with brine, dried over anhydrous Na_2_SO_4_, and concentrated under reduced pressure. The residue was purified by FCC (elution solvent: petroleum ether 35–60°C/ethyl acetate: 80/20) to afford **ENT-A009**, as a white solid (17.0 mg, 64% yield over two steps). **Rf:** 0.53 (petroleum ether 35–60°C/ethyl acetate: 6/4); **mp:** 205°C, decomposition; ${\left[a\right]}_{D}^{24}$ = + 59.26° (*c* = 0.00135 g/mL, CHCl_3_); ^**1**^**H NMR (600 MHz, CDCl**_**3**_**):** δ 6.91 (d, *J* = 16.7 Hz, 1H), 6.23 (bs, 1H), 5.38–5.30 (m, 1H), 5.00 (d, *J* = 16.8 Hz, 1H), 4.32 and 4.25 (two d, ABq, *J*_*AB*_ = 17.4 Hz, 2H), 3.60–3.43 (m, 1H), 2.55 (d, *J* = 18.8 Hz, 1H), 2.32–0.83 (m, 20H), 1.02 (s, 3H), 0.92 (s, 3H); ^**13**^**C NMR (151 MHz, CDCl**_**3**_**):** δ 149.0, 141.0, 135.9, 133.1, 121.4, 118.4, 93.3, 83.5, 71.9, 61.8, 51.5, 50.2, 46.0, 42.4, 37.4, 36.7, 33.7, 33.3, 32.7, 31.89, 31.77, 29.9, 23.5, 21.0, 19.6, 13.7. **HR-MS (APCI**^**−**^**):** m/z calculated for C_26_H_3_O_2_N 392.2595 [M-H]^−^, found 392.2587; **HPLC:** 100% purity, RT = 34.89 min. Column: Kromasil 100-10-SIL (10 × 250 mm, 10 μm), Method: eluting with 30% ethyl acetate-−70% cyclohexane, isocratic, flow rate 1.5 mL/min at 25°C, and injection volume 20 μL.

#### 2.1.11. (3S,17R)-5′-(cyclobutylidenemethyl)-3′,6′-dihydrospiro[5-androstene-17,2′-pyran]-3-ol (ENT-A065)

**Step 1**. Potassium *tert*-butoxide (53.9 mg, 0.48 mmol) was added to a solution of (4-bromobutyl)-triphenyl-phosphonium bromide (114.8 mg, 0.24 mmol) in anhydrous tetrahydrofuran (0.36 mL) at 0°C, and the reaction was stirred at 25°C for 30 min. The reaction was cooled to 0°C, a solution of aldehyde **10** (30.0 mg, 0.06 mmol) in anhydrous tetrahydrofuran (0.36 mL) was added, and the mixture was stirred at the same temperature for 1 h. The reaction was quenched, at the same temperature, by the dropwise addition of brine and extracted with ethyl acetate. The organic layer was dried over anhydrous Na_2_SO_4_ and concentrated *in vacuo*. The mixture was filtered through a silica pad (elution solvent: petroleum ether 35–60°C/ethyl acetate: 97/3) to remove triphenylphosphine oxide and the solvent evaporated *in vacuo*. The crude product was used without further purification in the next step (20 mg, 64% yield). **Rf:** 0.53 (petroleum ether 35–60°C/ethyl acetate: 94/6).

**Step 2**. HF·pyridine complex (52.0 μL, 0.58 mmol) was added dropwise to a solution of the crude product from step 1 (20.0 mg, 0.04 mmol) in anhydrous dichloromethane (1.3 mL) at 0°C, and the reaction was stirred at 0°C for 40 min. The reaction was quenched at 0°C with water and extracted with dichloromethane. The organic layer was washed with brine, dried over anhydrous Na_2_SO_4_, and concentrated under reduced pressure. The residue was purified by FCC (elution solvent: hexane/ethyl acetate: 7/3) to afford **ENT-A065**, as a white solid (17.0 mg, 64% yield over two steps). **Rf:** 0.23 (petroleum ether 35–60°C/ethyl acetate: 7/3); **mp:** 164–166°C; ${\left[a\right]}_{D}^{24}=+$ 6.58° (*c* = 0.00152 g/mL, CHCl_3_); **NMR (600 MHz, CDCl**_**3**_**):** δ 5.54 (bs, 1H), 5.52 (bs, 1H), 5.37–5.32 (m, 1H), 4.37 and 4.30 (two d, ABq, *J*_*AB*_ = 16.0 Hz, 2H), 3.56–3.47 (m, 1H), 2.89–2.79 (m, 2H), 2.72 (t, *J* = 8.1 Hz, 2H), 2.47–0.85 (m, 23H), 1.02 (s, 3H), 0.91 (s, 3H); ^**13**^**C NMR (151 MHz, CDCl**_**3**_**):** δ 141.4, 141.0, 135.0, 121.6, 120.77, 120.5, 83.1, 71.9, 64.5, 51.4, 50.3, 45.9, 42.4, 37.4, 36.7, 33.4, 33.2, 32.7, 32.4, 32.3, 31.9, 31.8, 31.8, 23.5, 21.0, 19.6, 17.4, 13.8. **HR-MS (APCI**^**+**^**):** m/z calculated for C_28_H_41_O_2_ 409.3101 [M+H]^+^, found 409.3101; **HPLC:** 100% purity, RT = 34.89 min. Column: Kromasil 100-10-SIL (10 × 250 mm, 10 μm), Method: eluting with 20% ethyl acetate −80% toluene, isocratic, flow rate 1.5 mL/min at 25°C, and injection volume 20 μL.

#### 2.1.12. (3S,17R)-5′-(2-bromovinyl)-3′,6′-dihydrospiro[5-androstene-17,2′-pyran]-3-ol (ENT-A066)

**Step 1**. NaHMDS (1M in tetrahydrofuran; 0.28 mL, 0.28 mmol) was added to a solution of (bromomethyl)-triphenyl-phosphonium bromide (112.1 mg, 0.28 mmol) in anhydrous tetrahydrofuran (0.42 mL) at 0°C, and the reaction was stirred at 25°C for 30 min. The reaction was cooled to 0°C, a solution of aldehyde **10** (33 mg, 0.07 mmol) in anhydrous tetrahydrofuran (0.42 mL) was added, and the mixture was stirred at the same temperature for 14 h. The reaction was quenched, at the same temperature, by the dropwise addition of brine and extracted with ethyl acetate. The organic layer was dried over anhydrous Na_2_SO_4_ and concentrated *in vacuo*. The mixture was filtered through a silica pad (elution solvent: petroleum ether 35–60°C/ethyl acetate: 94/6) to remove triphenylphosphine oxide and the solvent evaporated *in vacuo*. The crude product was used as such in the next step (28 mg, 72% yield). **Rf:** 0.63 (petroleum ether 35–60°C/ethyl acetate: 94/6).

**Step 2**. HF·pyridine complex (65.0 μL, 0.72 mmol) was added dropwise to a solution of the crude product from step 1 (28.0 mg, 0.05 mmol) in anhydrous dichloromethane (1.5 mL) at 0°C, and the reaction was stirred at 0°C for 40 min. The reaction was quenched at 0°C with water and extracted with dichloromethane. The organic layer was washed with brine, dried over anhydrous Na_2_SO_4_, and concentrated under reduced pressure. The residue was purified by FCC (elution solvent: hexane/ethyl acetate: 7/3) to afford **ENT-A066 (*E,Z* mixture 6:4)**, as a white solid (21.0 mg, 93% yield). **Rf:** 0.27 (petroleum ether 35–60°C/ethyl acetate: 7/3); **mp:** 68–70°C; [*a*]_*D*_^24^ = + 30.30° (*c* = 0.00165 g/mL, CHCl_3_); ^**1**^**H NMR (600 MHz, CDCl**_**3**_**):** δ 6.63 (d, *J* = 14.2 Hz) and 6.50 (d, *J* = 8.1 Hz) (1H), 6.05 and 5.82 (two bs, 1H), 6.04–5.97 (m, 1H), 5.37–5.32 (m, 1H), 4.66 and 4.58 (two d, ABq, *J*_*AB*_ = 16.5 Hz) and 4.31 and 4.26 (two d, ABq, *J*_*AB*_ = 15.4 Hz) (2H), 3.56–3.48 (m, 1H), 2.47–0.86 (m, 21H), 1.02 and 1.02 (two s, 3H), 0.93 and 0.91 (two s, 3H); ^**13**^**C NMR (151 MHz, CDCl**_**3**_**):** δ 141.0, 136.3, 133.4, 133.3, 131.6, 129.0, 126.6, 121.5, 103.2, 102.8, 83.5, 82.8, 71.9, 64.7, 62.4, 51.4, 50.2, 45.9, 42.4, 37.4, 36.7, 33.7, 33.5, 33.3, 33.2, 32.7, 32.2, 32.1, 31.8, 31.8, 29.8, 23.5, 21.0, 19.5, 13.7. **HR-MS (APCI**^**+**^**):** m/z calculated for C_25_H_36_O_2_Br 447.1893 [M+H]^+^, found 447.1892; **HPLC:** 100% purity, RT = 27.36 min (*E*) and 28.47 min (Z). Column: Kromasil 100-10-SIL (10 × 250 mm, 10 μm), Method: eluting with 25% ethyl acetate-−75% cyclohexane, isocratic, flow rate 1.5 mL/min at 25°C, and injection volume 20 μL.

#### 2.1.13. (E, Z) (3S,17R)-5′-(prop-1-en-1-yl)-3′,6′-dihydrospiro[5-androstene-17,2′-pyran]-3-ol (ENT-A068)

**Step 1**. Potassium *tert*-butoxide (26.9 mg, 0.24 mmol) was added to a solution of ethyl triphenyl-phosphonium bromide (89.1 mg, 0.24 mmol) in anhydrous tetrahydrofuran (0.42 mL) at 0°C, and the reaction was stirred at 25°C for 30 min. The reaction was cooled to 0°C, a solution of aldehyde **10** (50.0 mg, 0.10 mmol) in anhydrous tetrahydrofuran (0.42 mL) was added, and the mixture was stirred at the same temperature for 14 h. The reaction was quenched, at the same temperature, by the dropwise addition of brine and extracted with ethyl acetate. The organic layer was dried over anhydrous Na_2_SO_4_ and concentrated *in vacuo*. The mixture was filtered through a silica pad (elution solvent: petroleum ether 35–60°C/ethyl acetate: 94/6) to remove triphenylphosphine oxide and the solvent evaporated *in vacuo*. The crude product was used as such in the next step (28 mg, 94% yield). **Rf:** 0.63 in petroleum ether 35–60°C/ethyl acetate: 94/6).

**Step 2**. HF·pyridine complex (78.0 μL, 0.87 mmol) was added to a solution of the crude product from step 1 (28 mg, 0.06 mmol) in anhydrous dichloromethane (1.8 mL) at 0°C, and the reaction was stirred at 0°C for 40 min. The reaction was quenched at 0°C with water and extracted with dichloromethane. The organic layer was washed with brine, dried over anhydrous Na_2_SO_4_, and concentrated under reduced pressure. The residue was purified by FCC (elution solvent: hexane/ethyl acetate: 7/3) to afford **ENT-A068 (*E, Z*** mixture), as a white solid (20 mg, 87% over two steps); **Rf:** 0.22 (petroleum ether 35–60°C/ethyl acetate: 7/3); **mp:** 103–105°C; ${\left[a\right]}_{D}^{24}=-36.23^{•}$(*c* = 0.00138 g/mL, CHCl_3_); ^**1**^**H NMR (600 MHz, CDCl**_**3**_**):** δ 5.95 (d, *J* = 16.0 Hz, 0.4H), 5.68–5.63 (m, 1.6H), 5.50–5.45 (m, 0.6 H), 5.40–5.34 (m, 1.4H), 4.36–4.26 (m, 2H), 3.56–3.49 (m, 1H), 2.48–1.19 (m, 19H), 1.76 (d, *J* = 7.2 Hz) and 1.73 (d, *J* = 6.6 Hz) (3H), 1.024 and 1.023 (two s, 3H), and 0.92 (s, 3H). ^**13**^**C NMR (151 MHz, CDCl**_**3**_**):** ^**13**^**C NMR (151 MHz, CDCl**_**3**_**):** δ 141.0, 134.4, 134.1, 130.4, 127.8, 125.3, 123.2, 122.3, 122.0, 121.5, 83.6, 83.0, 71.9, 65.7, 63.3, 51.4, 50.3, 45.9, 42.4, 37.4, 36.7, 33.7, 33.5, 33.3, 33.2, 32.7, 31.9, 31.8, 31.7, 23.5, 21.0, 19.6, 18.6, 15.7, 14.3, 13.8; **HR-MS (APCI**^**+**^**):** m/z calculated for C_26_H_39_O_2_ 383.2945 [M+H]^+^, found 383.2941; **HPLC:** 100% purity, RT = 25.17 min. Column: Kromasil 100-10-SIL (10 × 250 mm, 10 μm), Method: eluting with 25% ethyl acetate −75% cyclohexane, isocratic, flow rate 1.5 mL/min at 25°C, and injection volume 20 μL.

#### 2.1.14. (3*S*,17*R*)-5′-ethyl-3′,6′-dihydrospiro[5-androstene-17,2′-pyran]-3-ol (ENT-A070)

Methyl magnesium bromide (3M in Et_2_O, 40.5 μL, 0.12 mmol) was added to a solution of **ENT-A025** (31.7 mg, 0.081 mmol) in anhydrous tetrahydrofuran (0.81 mL) at 0°C, and the resulting solution was stirred at 25°C for 12 h. The reaction was quenched with a saturated aqueous solution of ammonium chloride and concentrated under reduced pressure. The residue was solubilized in ethyl acetate and washed with brine. The organic layer was dried over anhydrous Na_2_SO_4_ and concentrated under reduced pressure. The residue was purified by FCC (elution system: hexane/ethyl acetate: 7/3) to obtain **ENT-A070** as a white solid (27 mg, 90% yield). **Rf:** 0.30 (petroleum ether 35–60°C/ethyl acetate: 6/4); **mp:** 137–139°C; ${\left[a\right]}_{D}^{24}=-$ 5.26° (*c* = 0.00129 g/mL, CHCl_3_); ^**1**^**H NMR (600 MHz, CDCl**_**3**_**):** δ 5.42 (bs, 1H), 5.35–5.31 (m, 1H), 4.08 and 4.04 (two d, ABq, *J*_*AB*_ = 18.4 Hz, 2H), 3.54–3.46 (m, 1H), 2.36–0.85 (m, 23H), 1.01 (s, 3H), 0.98 (t, *J* = 7.4 Hz, 3H), 0.89 (s, 3H). ^**13**^**C NMR (151 MHz, CDCl**_**3**_**):** δ 140.8, 137.5, 121.4, 115.9, 83.1, 71.7, 65.6, 51.2, 50.09, 45.1, 42.2, 37.3, 36.5, 33.3, 33.1, 32.5, 31.7, 31.6, 31.1, 25.3, 23.30, 20.9, 19.4, 13.6, 12.1; **HR-MS (APCI**^**+**^**):** m/z calculated for C_25_H_39_O_2_ 371.2945 [M+H]^+^, found 371.2947; **HPLC:** 96.5% purity, RT = 27.36 min. Column: Kromasil 100-10-SIL (10 × 250 mm, 10 μm), Method: eluting with 25% ethyl acetate −75% cyclohexane, isocratic, flow rate 1.5 mL/min at 25°C, and injection volume 20 μL.

#### 2.1.15. (3S,17R)-5′-(ethyl)-3′,6′-tetrahydrospiro[5-androstene-17,2′-pyran]-3-ol (ENT-A069)

A round-bottom flask was loaded with **ENT-A070** (20 mg, 0.03 mmol) and tetrahydrofuran (0.5 mL). The solution was reduced using H-Cube Mini+ over 5% PtS/C, at 25°C, hydrogen pressure 1 atm, and flow rate 1 mL/min. The reaction mixture was circulated three times through the 5% PtS/C cartridge until the reaction was complete (checked by ^1^H-NMR). The reaction was concentrated *in vacuo* and the residue was purified by FCC (elution solvent: ethyl acetate/hexane/ethyl acetate: 7/3) to afford **ENT-A069 (**mixture of C5′ epimers), as a white solid (15 mg, 70% yield). **Rf:** 0.54 (petroleum ether 35–60°C/ethyl acetate: 6/4); **mp:** 107–109°C; ${\left[a\right]}_{D}^{24}=-$ 40.65° (*c* = 0.00123 g/mL, CHCl_3_); ^**1**^**H NMR (600 MHz, CDCl**_**3**_**):** δ 5.22–5.18 (m, 1H), 3.60 (dd, *J* = 12.2, 4.2 Hz) and 2.93 (t, *J* = 11.3 Hz) (1H), 3.49 (dd, *J* = 11.5, 3.2 Hz) and 3.24 (dd, *J* = 11.5, 5.9 Hz) (1H), 3.44–3.31 (m, 1H), 2.18–0.67 (m, 26H), 0.88 (s, 3H), 0.77–0.71 (m, 3H), 0.74 and 0.70 (s, 3H). ^**13**^**C NMR (75 MHz, CDCl**_**3**_**):** δ 141.0, 121.6, 84.8, 84.7, 71.9, 69.4, 68.2, 51.4, 50.4, 50.2, 46.6, 46.2, 42.4, 38.0, 37.4, 37.4, 36.7, 36.7, 36.3, 35.8, 35.3, 32.7, 32.6, 32.1, 32.0, 31.9, 31.8, 30.4, 30.0, 26.5, 26.0, 25.5, 24.2, 24.0, 23.7, 21.2, 20.9, 19.5, 14.2, 13.9, 11.9, 11.4; **HR-MS (APCI**^**+**^**):** calculated for C_25_H_41_O_2_ m/z 373.3101 [M+H]^+^, found m/z 373.3100; **HPLC:** 100% purity, RT = 24.99 min. Column: Kromasil 100-10-SIL (10 × 250 mm, 10 μm), Method: eluting with 12% ethyl acetate −88% toluene, isocratic, flow rate 2.1 mL/min at 25°C, and injection volume 20 μL.

#### 2.1.16. (3S)-3-hydroxy-spiro[5-androsten-17-cyclobutan]-3′-one (ENT-A076)

Potassium carbonate (611 mg, 4.42 mmol) was added to a solution of compound **13** (480 mg, 1.34 mmol) in a mixture of methanol/water (54/1, 547 mL). The resulting solution was stirred at 25°C for 4 h. Upon completion of the reaction, the reaction mixture was concentrated under reduced pressure, and the residue was extracted with ethyl acetate. The organic layer was washed with brine, dried over anhydrous Na_2_SO_4_, and concentrated under reduced pressure. The residue was purified by FCC (elution solvent: hexane/ethyl acetate: 8/2) to afford **ENT-A076**, as a white crystalline solid (440 mg, quantitative yield). **Rf:** 0.13 (petroleum ether 35–60°C/ethyl acetate: 8/2); **mp:** 183–185°C; ${\left[a\right]}_{D}^{24}=-$130.77° (*c* = 0.00130 g/mL, CHCl_3_); ^**1**^**H NMR (600 MHz, CDCl**_**3**_**):** δ 5.34–5.30 (m, 1H), 3.53–3.45 (m, 1H), 3.08 (d, *J* = 17.3 Hz, 1H), 3.00 (d, *J* = 17.3 Hz, 1H), 2.64 (dd, *J* = 17.3, 3.4 Hz, 1H), 2.43 (dd, *J* = 17.3, 3.4 Hz, 1H), 2.29–0.75 (m, 19H), 1.00 (s, 3H), 0.77 (s, 3H); ^**13**^**C NMR (151 MHz, CDCl**_**3**_**):** δ 208.3, 140.9, 121.4, 71.7, 54.3, 52.6, 51.4, 50.0, 43.4, 42.8, 42.3, 37.5, 37.4, 36.6, 33.0, 32.0, 31.9, 31.6, 24.7, 20.6, 19.5, 14.6. **HR-MS (APCI**^**+**^**):** m/z calculated for C_22_H_33_O_2_ 329.2475 [M+H]^+^, found 329.2474; **HR-MS (APCI**^**+**^**):** m/z calculated for C_22_H_33_O_2_ 329.2475 [M+H]^+^, found 329.2474; **HPLC:** 100% purity, RT = 31.56 min. Column: Kromasil 100-10-SIL (10 × 250 mm, 10 μm), Method: eluting with 20% ethyl acetate −80% toluene, isocratic, flow rate 1.5 mL/min at 25°C, and injection volume 20 μL.

#### 2.1.17. (E, Z) (3S)-3-hydroxy-spiro[5-androsten-17-cyclobutan]-3′-ylidene)acetonitrile (ENT-A077)

Diethyl (cyanomethyl) phosphonate (64.7 μL, 0.40 mmol) was added to a suspension of NaH (60% dispersion in mineral oil, 16 mg, 0.40 mmol) in anhydrous tetrahydrofuran (0.3 mL) at 0°C, and the reaction mixture was stirred at 25°C for 30 min. The reaction was cooled to 0°C, a solution of **ENT-A076** (38 mg, 0.10 mmol) in anhydrous tetrahydrofuran (1.0 mL) was added, and the mixture was stirred at 25°C for 12 h. The reaction was quenched, at 0°C, by the dropwise addition of brine, and the mixture was concentrated *in vacuo*. The residue was extracted with ethyl acetate, and the organic layer was washed with brine, dried over anhydrous Na_2_SO_4_, and concentrated under reduced pressure. The residue was purified by FCC (elution solvent: hexane/ethyl acetate: 8/2) to obtain **ENT-A077** (***E, Z*** mixture *1/1)*, a white crystalline solid (25 mg, 71% yield). **Rf:** 0.05 (petroleum ether 35–60°C/ethyl acetate: 8/2); **mp:** 161–163°C; ${\left[a\right]}_{D}^{24}=-141.84^{•}$(*c* = 0.00141 g/mL, CHCl_3_); ^**1**^**H NMR (600 MHz, CDCl**_**3**_**):** δ 5.36–5.32 (m, 1H), 5.15 and 5.13 (two s, 1H), 3.56–3.50 (m, 1H), 2.99 (d, *J* = 16.6 Hz) and 2.78 (d, *J* = 16.6 Hz) (1H), 2.89 (t, *J* = 17.4 Hz, 1H), 2.64–0.86 (m, 21H), 1.01 (s, 3H), 0.69 and 0.67 (two s, 3H). ^**13**^**C NMR (75 MHz, CDCl**_**3**_**):** δ 167.9, 140.9, 121.6, 116.7, 110.2, 91.6, 71.9, 51.9, 50.1, 48.2, 43.4, 42.4, 41.3, 38.0, 37.8, 37.4, 37.3, 36.7, 32.9, 32.0, 31.7, 29.8, 24.6, 20.7, 19.6, 13.8. **HR-MS (APCI**^**+**^**):** m/z calculated for C_24_H_34_ON 352.2635 [M+H]^+^, found 352.2635; **HPLC:** 100% purity, RT = 31.56 min. Column: Kromasil 100-10-SIL (10 × 250 mm, 10 μm), Method: eluting with 30% ethyl acetate −70% cyclohexane, isocratic, flow rate 1.5 mL/min at 25°C, and injection volume 20 μL.

#### 2.1.18. (E, Z) Ethyl (3S)-3-hydroxy-spiro[5-androsten-17-cyclobutan]-3′-ylidene)acetate (ENT-A079)

Triethyl phosphonoacetate (1.0 mL, 5.08 mmol) was added to a suspension of NaH (60% dispersion in mineral oil, 203 mg, 0.51 mmol) in anhydrous tetrahydrofuran (3.8 mL) at 0°C, and the reaction mixture was stirred at 25°C for 30 min. The solution was cooled to 0°C, a solution of **ENT-A076** (469 mg, 1.27 mmol) in anhydrous tetrahydrofuran (12.7 mL) was added dropwise, and the mixture was stirred at 25°C for 12 h. The reaction was quenched, at 0°C, by the dropwise addition of brine, and the mixture was concentrated under reduced pressure. To the residue was added ethyl acetate, and the organic phase was washed with brine and dried over anhydrous Na_2_SO_4_ and was concentrated *in vacuo*. The residue was purified by FCC (elution solvent: hexane/ethyl acetate: 7/3) to obtain **ENT-A079 (*E,Z* mixture 1:1)** as a solid (122 mg, 32% yield). **Rf:** 0.14 (petroleum ether 35–60°C/ethyl acetate: 7/3); ^**1**^**H NMR (300 MHz, CDCl**_**3**_**):** δ 5.68–5.58 (m, 1H), 5.39–5.31 (m, 1H), 4.21–4.05 (m, 2H), 3.59–3.46 (m, 1H), 3.19–0.93 (m, 23H), 1.33–1.19 (m, 3H), 1.02 (s, 3H), and 0.70 and 0.67 (two s, 3H).

#### 2.1.19. (3S)-3-hydroxy-spiro[5-androsten-17-cyclobutan]-3′-ylidene) acetic acid (ENT-A080)

NaOH (70 mg, 1.75 mmol) was added to a mixture of **ENT-A079** (*E/Z* mixture 1:1) (99 mg, 0.25 mmol) in a mixture of EtOH/water (2.5 mL/0.75 mL), and the resulting mixture was refluxed for 4 h. The reaction was cooled to room temperature, and the mixture was concentrated under reduced pressure. To the residue was added dropwise 10% HCl_aq_ with stirring until a pale-yellow precipitate formed, which was filtered, and the solid was washed with hexane and Et_2_O, resulting in a white solid. The solid was then dissolved in tetrahydrofuran, and the solution was dried over anhydrous Na_2_SO_4_ and concentrated under reduced pressure. The residue was purified by FCC (elution solvent: ethyl acetate) to obtain **ENT-A080 (*E,Z* mixture 1:1)**, as a white crystalline solid (87 mg, 94% yield). **Rf:** 0.58 (ethyl acetate); **mp:** 221°C, decomposition; ${\left[a\right]}_{D}^{24}=-$ 73.53° (*c* = 0.00136 g/mL, CHCl_3_); ^**1**^**H NMR (600 MHz, CDCl**_**3**_**):** δ 5.66 and 5.64 (two s, 1H), 5.39–5.32 (m, 1H), 3.58–3.50 (m, 1H), 3.19–0.62 (m, 23H), 1.02 (s, 3H), 0.69 and 0.68 (two s, 3H). ^**13**^**C NMR (151 MHz, CDCl**_**3**_**):** δ 171.4, 166.9, 140.7, 121.5, 111.9, 71.7, 51.5, 50.0, 48.8, 43.2, 42.6, 42.2, 37.8, 37.3, 36.6, 32.7, 32.0, 31.8, 31.6, 29.7, 29.4, 24.5, 22.7, 20.6, 19.4, 14.1, 13.7. **HR-MS (APCI**^**−**^**):** m/z calculated for C_24_H_33_O_3_ 369.2435 [M-H]^−^, found m/z 369.2421; **HPLC:** 100% purity, RT = 18.88 min and 19.92 min (each peak corresponding to one geometrical isomer). Column: Kromasil 100-10-SIL (10 × 250 mm, 10 μm), Method: eluting with 50% ethyl acetate −50% toluene, isocratic, flow rate 1.5 mL/min at 25°C, and injection volume 20 μL.

#### 2.1.20. (E,Z) Methyl (3S)-3-hydroxy-spiro[5-androsten-17-cyclobutan]-3′-ylidene)acetate (ENT-A087)

NaOH (50 mg, 1.25 mmol) was added to a solution of **ENT-A079** (*E,Z* mixture 1:1) (459 mg, 1.24 mmol) in MeOH/water: (54/1; 10.6 mL/0.2 mL). The mixture was stirred at 25°C for 12 h. The reaction mixture was concentrated at reduced pressure and to the residue was added dropwise with stirring 10% HCl_aq_ until a pale-yellow precipitate formed which was filtered, and the solid was washed with hexane and Et_2_O, affording a white solid. The solid was then dissolved in tetrahydrofuran, and the solution was dried over anhydrous Na_2_SO_4_ and concentrated under reduced pressure. The residue was purified by FCC (elution solvent: hexane/ethyl acetate: 7/3) to obtain **ENT-A080** (*E, Z* mixture 1:1, 144 mg, 31% yield), **ENT-A087** (*E,Z* mixture 1:1; 100 mg, 21% yield), and **ENT-A088** (mixture of the two C17 epimers in a 7/3 ratio; 33 mg, 7% yield), as white solids.

**ENT-A087** (*E, Z* mixture 1:1) **Rf:** 0.12 (petroleum ether 35-60°C/ethyl acetate: 8/2); **mp:** 87–89°C; ${\left[a\right]}_{D}^{24}=-150.79^{•}$ (*c* = 0,00126 g/mL, CHCl_3_); ^**1**^**H NMR (600 MHz, CDCl**_**3**_**):** δ 5.63 and 5.61 (two s, 1H), 5.34–5.30 (m, 1H), 3.66 and 3.65 (two s, 3H), 3.54–3.45 (m, 1H), 3.14–0.87 (m, 23H), 0.99 (s, 3H), 0.67 and 0.65 (two s, 3H). ^**13**^**C NMR (75 MHz, CDCl**_**3**_**):** δ 167.2, 163.6, 140.9, 121.5, 112.2, 71.7, 71.7, 51.8, 51.0, 50.1, 49.0, 43.3, 42.3, 41.0, 38.8, 37.6, 37.4, 36.6, 32.8, 32.0, 31.6, 24.6, 24.6, 20.7, 19.5, 13.7. **HR-MS (APCI**^**+**^**):** m/z calculated for C_25_H_37_O_3_ [M+H]^+^ 385.2737, found 385.2735; **HPLC:** 100% purity, RT = 35.65 min and 38.66 min (each peak corresponding to one geometrical isomer). Column: Kromasil 100-10-SIL (10 × 250 mm, 10 μm), Method: eluting with 25% ethyl acetate −75% cyclohexane, isocratic, flow rate 1.5 mL/min at 25°C, and injection volume 20 μL.

**ENT-A088** (17*R,S*) (3*S*)-3-hydroxy-spiro[5-androsten-17,3′-oxepan]-7′-one **Rf:** 0.28 petroleum ether 35–60°C/ethyl acetate: 6/4); **mp:** 181–183°C; ${\left[a\right]}_{D}^{24}=-33.06^{•}$(*c* = 0.00121 g/mL, CHCl_3_); ^**1**^**H NMR (600 MHz, CDCl**_**3**_**):** δ 5.36–5.32 (m, 1H), 4.30 (d, *J* = 8.8 Hz) and 4.23 (d, *J* = 9.0 Hz) (1H), 4.05 (d, *J* = 9.0 Hz) and 3.88 (d, *J* = 8.8 Hz, 1H), 3.51 (s, 1H), 2.59–0.75 (m, 25H), 1.01 (s, 3H), and 0.78 and 0.76 (two s, 3H). ^**13**^**C NMR (75 MHz, CDCl**_**3**_**):** δ 177.1, 140.9, 121.3, 76.0, 75.0, 71.7, 60.5, 53.3, 53.1, 52.7, 50.0, 43.6, 42.3, 38.4, 37.4, 36.7, 36.1, 35.9, 34.8, 32.6, 32.5, 32.3, 32.1, 32.0, 31.9, 31.7, 24.5, 24.3, 20.6, 19.5, 16.1, 14.7, 14.3; **HPLC:** 100% purity, RT = 35.29 min and 37.43 min. Column: Kromasil 100-10-SIL (10 × 250 mm, 10 μm), Method: eluting with 25% ethyl acetate −75% toluene, isocratic, flow rate 1.5 mL/min at 25°C, and injection volume 20 μL.

### 2.2. Biological evaluation

#### 2.2.1. Screening for TrkA activity and related assays

##### 2.2.1.1. Cell lines

PC12 cells were obtained from LGC Promochem and cultured under specified conditions. Cells were grown in DMEM medium (Thermo Fischer Scientific, cat# 10566016) containing 10% horse serum (Thermo Fischer Scientific, cat# 16050122), 5% fetal bovine serum (Thermo Fischer Scientific, cat# 10270106), 100 units/mL penicillin, and 0.1 mg/mL streptomycin (Thermo Fischer Scientific, cat# 15140122) at 5% CO_2_ and 37°C. Cells were used between passages 5 and 20.

##### 2.2.1.2. Immunoprecipitation and Immunoblotting

For immunoprecipitation experiments, PC12 cells were used when they were at 70–80% confluent. Cells were starved from serum for 4 h and subsequently treated with 100 ng/mL NGF (Alomone Labs, cat# N-100) or 500 nM of each compound for 30 min. Cells were then lysed in Pierce™ IP Lysis Buffer (Thermo Fischer Scientific, cat# 87787) containing proteases (Sigma-Aldrich, cat# 539131) and phosphatase inhibitors (Millipore, cat# 524629). Lysates were then immunoprecipitated overnight at 4°C with TrkA antibody (1:100, Millipore, cat# 06-574) followed by 4 h incubation with protein G-plus agarose beads (Santa Cruz Biotechnology, cat# sc-2002). Beads were then collected, washed three times with lysis buffer, resuspended in SDS loading buffer, and subjected to Western blot against phosphorylated Tyrosine (1:1000, R&D systems, cat# BAM1676). Whole-cell lysates were subjected to Western blot against TrkA (1:1,000, Millipore, cat# 06-574), phosphorylated Akt (1:1,000, Cell Signaling, cat# 9271S), phosphorylated Erk1/2 (1:1,000, Cell Signaling, cat# 9101), total Akt (1:1,000, Cell Signaling, cat# 4,691), and total Erk1/2 (1:1000, Cell Signaling, cat# 9,194).

##### 2.2.1.3. CellTox assay

CellTox assay (G8742, Promega, Leiden, Belgium) was used to assess the survival of PC12 cells under serum deprivation conditions. PC12 cells were plated in 96-well plates, starved from serum for 4 h, and subsequently treated with NGF (100 ng/mL) or compound (500 nM) for 24 h. CellTox assay reagents and Hoescht (1:10,000, H3570, Invitrogen, Massachusetts, USA) were then added to each well for 30 min, and then, cells were imaged with a Zeiss AXIO Vert A1 fluorescent microscope. CellTox positive cells were normalized to the total number of cells for each image.

#### 2.2.2. Screening for TrkB activity and related assays

##### 2.2.2.1. Cell culture

We tested compounds on three cellular systems, primary cortical astrocytes from 2-day-old mice (as a natively TrkB-expressing neuronal population), and NIH-3T3 fibroblasts stable transfected with TrkB and NIH-3T3 naïve cells. Cells were grown with high-glucose DMEM medium supplemented with 10% fetal bovine serum (FBS), 100 μg/mL streptomycin, and 100 units/mL penicillin at 37°C in a humidified 5% CO_2_ atmosphere. Phosphorylation results presented here are in primary astrocytes, and cell toxicity assay results are in NIH-3T3 stable transfected cells with TrkB.

##### 2.2.2.2. Western blot

Cells were plated in 12 well plates, at a density of 100,000 cells per well, with 6 h of serum deprivation carried out on the following day. To assess the effect of compounds on TrkB phosphorylation, 20-min treatments were applied, using either BDNF at 500 ng/mL (Peprotech 450-02) (positive control) or compounds at 1 μM.

Cell lysis for 10 min was carried out on ice using the Pierce IP Lysis Buffer by Thermo Scientific and a phospho-protease inhibitor cocktail by Millipore. After adding loading buffer (5 × Laemni), 25 μg from each protein sample were incubated for 5 min at 95°C and subjected to SDS-PAGE. Proteins were transferred to a nitrocellulose membrane at 350 mA for 2 h. Membrane blocking was performed using 5% bovine serum albumin (BSA) at room temperature for 1 h, before adding the primary antibodies to the blocking solution at 4°C overnight. HRP-conjugated secondary antibodies were used for the detection of chemiluminescence with ECL solution. Primary antibodies: Phospho-TrkB (Tyr816) Millipore # ABN1381, and Anti-TrkB Abcam #ab33655.

##### 2.2.2.3. CellTox green cytotoxicity assay

NIH-3T3 stable transfected cells with TrkB were plated in 96-well plates at a density of 10,000 cells/well, before carrying out serum starvation for 24 h. Cells were then treated for 24 h with either BDNF at 500 ng/mL (control) or the compounds at 1 μM.

For the cell toxicity assay, we used CellTox™ Green Dye (2,000 × , Promega) for the visualization of dead cells and Hoechst 33,342 solution (10,000 × ) for total cells. A ZEISS Axio Vert.A1 microscope was used for image capture, and the ImageJ software (https://imagej.nih.gov/ij/) was used for analysis. The number of CellTox positive cells was normalized against the total number of cells observed in each image.

##### 2.2.2.4. Primary astrocytes

Mixed glial cultures were isolated from the cortex of C57B/6 pups at post-natal day 2 (P2). Cells were plated in a medium containing high-glucose DMEM, 200 U/mL penicillin, 200 μg/mL streptomycin, and 10% fetal bovine serum (FBS). When cells reach 100% confluency (7–8 days), the anti-mitotic agent Ara-C was added in the media at a final concentration of 10 μM, for 3 to 4 days to target the highly proliferative microglial cells. Ara-C was removed, and primary astrocytes (97% purity) were cultured at 5% CO_2_ and 37°C.

##### 2.2.2.5. Immunoprecipitation and Immunoblotting

Cells were plated at 70–80% confluency. The next day, they were deprived of serum for 6 h and subsequently treated with 500 ng/mL BDNF or 1 μM of compounds for 20 min. Cells were then lysed in Pierce™ IP Lysis Buffer (87788, Thermo Fischer Scientific, Rockford, USA), containing proteases (539138, Calbiochem, Darmstadt, Germany), and phosphatase inhibitors (524629, Calbiochem, Darmstadt, Germany). Lysates were then suspended in an SDS loading buffer and subjected to Western blot against phosphorylated TrkB (1:1,000, ABN1381, Sigma-Aldrich, St. Louis, MO, USA) and total TrkB (1:1,000, 07-225-I, Sigma-Aldrich, St. Louis, MO, USA).

##### 2.2.2.6. Quantitative RT-PCR

Total RNA was extracted from cells using TRIzol reagent (15596026, Thermo Fisher, Waltham, MA, USA), and cDNA was synthesized using the High-Capacity cDNA Reverse Transcription kit (4368814, Thermo Fisher, Waltham, MA, USA) according to the supplier protocols. For qPCR experiments run with SYBR green dye, for 20 s at 95°C, followed by 40 cycles of 95°C for 3 s and 60°C for 30 s on a StepOne Real-Time PCR System (Thermo Fisher Scientific, Waltham, MA, USA). B-Actin was used as a housekeeping gene to normalize the gene expression levels. Data were collected and analyzed using the StepOne Software v2.3 (Thermo Fischer Scientific, Waltham, MA, USA).

##### 2.2.2.7. Statistical analysis

The data are presented as the mean ± standard error of the mean (SEM). Statistical analysis was conducted using GraphPad Prism 7 software (GraphPad Software Inc., San Diego, CA, USA). Student's *t-*test was used for the comparisons. A *p*-value of < 0.05 was considered to mark statistical significance. In cases where fold change values were utilized, statistical analysis was performed on the portion of each case normalized to the DMSO condition. A *p-*value of < 0.05 was considered statistically significant.

### 2.3. Prediction of physicochemical descriptors and ADME properties

The physicochemical descriptors and ADME properties of the compounds were predicted through the application of the Qikprop module of the Schrödinger platform (Schrodinger Release 2020-3, 2020). Before Qikprop calculations, the compounds were imported in the Maestro platform of Schrodinger software as .smi files (Schrödinger Release 2020-3, 2020a), converted to 3D form, and optimized through the energy minimization module of MacroModel (Schrödinger Release 2020-3, 2020b) and Ligprep module (Schrödinger Release 2020-3, 2020c) by applying OPLS_2005 force field at pH = 7.0 ± 0.5.

### 2.4. Assessment of metabolic stability

For metabolic stability assessment, pooled human liver microsomes were employed at 0.5 mg/mL. Incubation conditions were optimized to ensure linear metabolite formation with respect to protein concentration and reaction time. All test compounds were assessed at 1 μM based on our ADMET standard operating procedures and protocols, also published previously (1 μM was selected based on our ADMET standard operating procedures and protocols also published previously) (Rogdakis et al., 2022; Yilmaz et al., 2022). Approximately 1 mM of NADPH served as a cofactor. Reactions took place in triplicates at 37°C. Negative and positive controls (low vs. rapid clearance) were included for low vs. rapid clearance. After 60 min, reactions were terminated. To profile the depletion of test compounds and hence determine the residual (%) of time zero, readouts were recorded by Lionheart FX (Agilent BioTek).

### 2.5. Isozyme-specific CYP450 metabolism

Human cytochrome P450 (CYP450) isoenzymes were expressed in Baculosomes^®^, purchased from Thermo Fisher Scientific (Waltham, MA, USA). All reagents were handled and prepared according to the manufacturer's protocol. Test compounds were assessed at 1 μM for their effects on CYP1A2, CYP2A6, CYP2C9, CYP2C19, CYP2D6, and CYP3A4. Approximately 1 μM was selected based on our ADMET standard operating procedures and protocols also published previously (Rogdakis et al., 2022; Yilmaz et al., 2022). CYP450-enzymatic activity was determined based on the kinetic model for each CYP450 isoform (Cohen et al., 2003; Veith et al., 2009). Reactions took place in triplicates at 20°C. For this, NADP^+^ (10 mM in 100 mM potassium phosphate, pH 8.0) was converted into NADPH by the regeneration system present (glucose-6-phosphate at 333 mM and glucose-6-phosphate-dehydrogenase at 30 U/mL in 100 mM potassium phosphate, pH 8.0). Following the addition of the fluorescent substrate, signal monitoring over time took place immediately (<2 min) at suitable excitation and emission wavelengths (Agilent BioTek Lionheart FX). CYP450 inhibition (%) was determined based on the reaction rates (fluorescence intensity changes per unit time). In total, *n* = 60 measurements per minute were acquired (*t* = 60 min).


$$\begin{array}{l} \%\;Inhibition=\left(1-\frac{X}{A}\right)\times 100\% \end{array}$$

X is the rate observed in the presence of the test compound.

A is the rate observed in the presence of the negative (solvent, DMSO) control.

## 3. Results

### 3.1. Chemistry

#### 3.1.1. C17-spiro-(2H)dihydropyran derivatives

**Figure 2:**
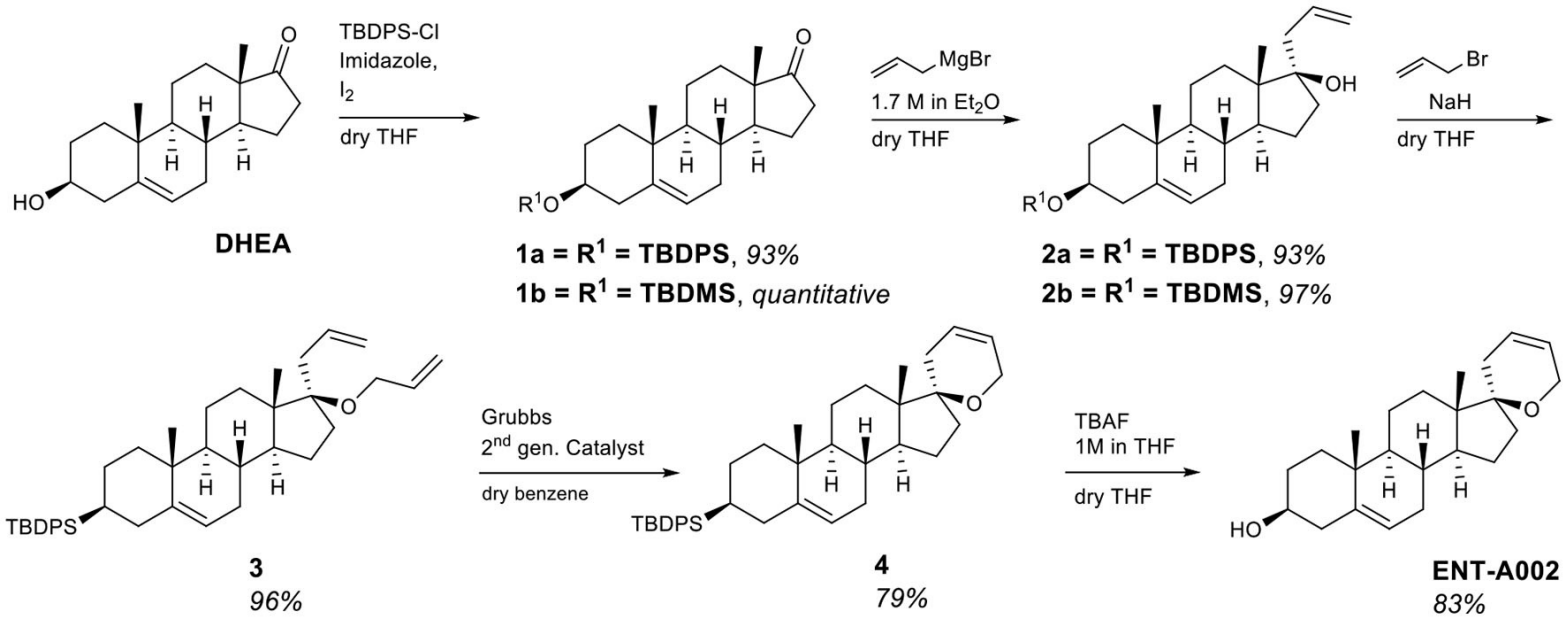
Synthesis of **ENT-A002**.

The first series of derivatives involves the modification of the DHEA scaffold by a C17-spiro-2*H*-dihydropyran ring variously decorated at the C5′ position. Initially, the unsubstituted derivative **ENT-A002** was prepared from DHEA. Quantitative protection of the 3β-hydroxy group of DHEA as the tert-butyldiphenylsilyl ether (**1a**) was achieved using tert-butyldiphenylsilyl chloride in the presence of imidazole as the base and iodine. The steric hindrance imposed by the axial C18 methyl group on the C17-ketone (Xun et al., 1994) resulted in the exclusive addition of allylmagnesium bromide from the equatorial face of compound **1a** with a conversion of 97%. The resulting tertiary alcohol (**2a**) was then deprotonated with NaH and alkylated by allyl bromide, yielding compound **3** in 97% yield. The desired (2*H*)dihydropyran ring was formed via a ring-closing metathesis reaction using diene **3** and Grubbs 2nd generation catalyst affording derivative **4** in 94% yield. Finally, the deprotection of the C3-alcohol of **4** was achieved using tetra-n-butylammonium fluoride (TBAF) to give compound **ENT-A002** an 83% yield (Figure 2).

**Figure 3:**
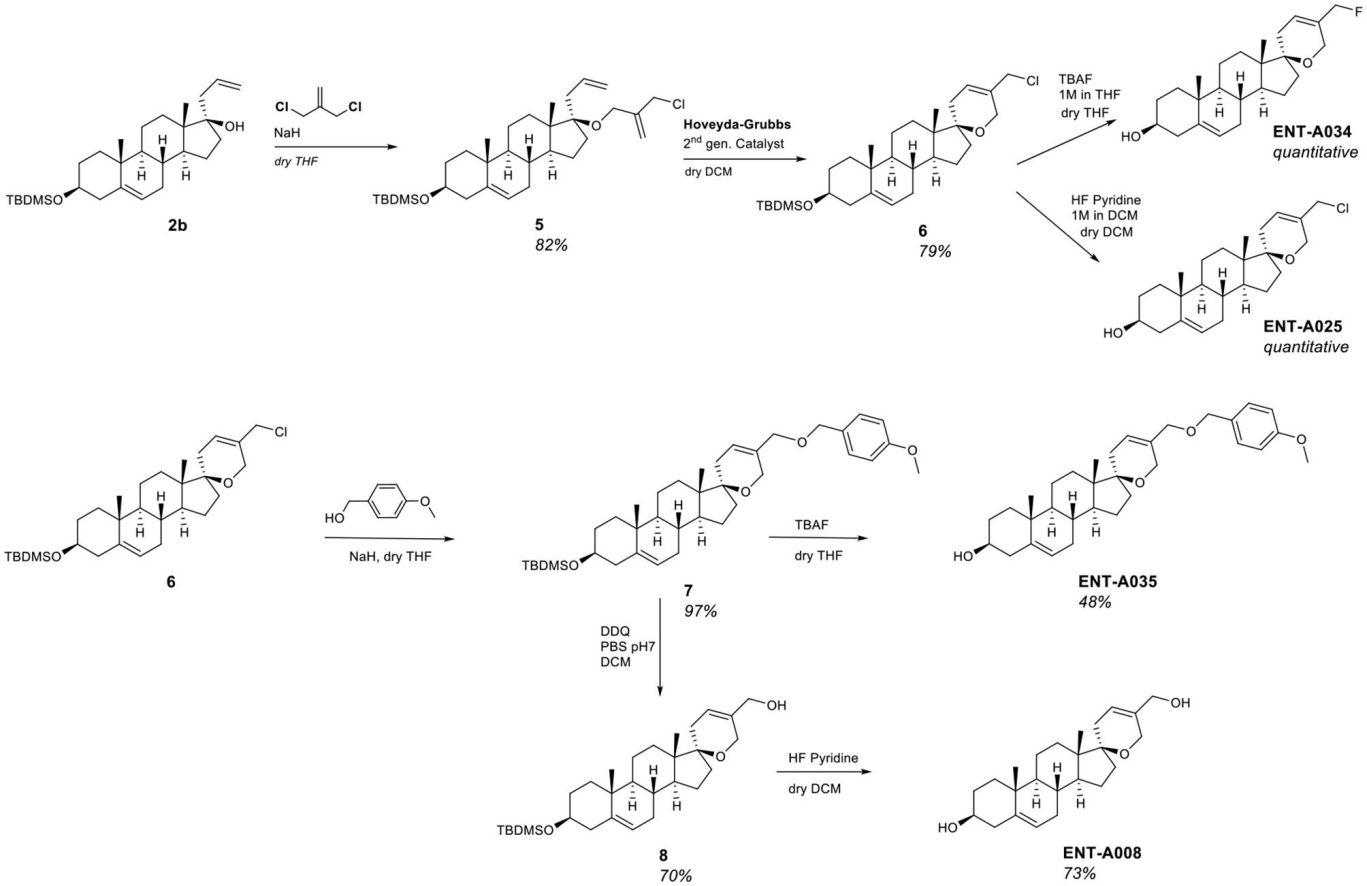
Synthesis of the key intermediate **6** and compounds **ENT-A008**, **ENT-A025**, **ENT-A034**, and **ENT-A035**.

Compound **ENT-A002** represents the unsubstituted canvas of this series. To explore the stereo-electronic requirements for selectivity and agonism on the neurotrophin Trk receptors, we incorporated a chloromethyl handle, on the C17-spiro-(2*H*)dihydropyran moiety (compound **6**) that could further elaborate a variety of functionalities. Our synthetic strategy involved the protection of the C3-alcohol of DHEA with tert-butyldimethylchlorosilane to afford compound **1b** in quantitative yield that was in turn transformed to compound **2b** upon the addition of allyl magnesium bromide (Figure 2). Alkylation of the tertiary alcohol of compound **2b** using 3-chloro-2-chloromethyl-propene and NaH as a base afforded the chloro-substituted diene **5** in 82% yield (Figure 3). Subsequently, a ring-closing metathesis reaction employing Hoveyda-Grubbs 2^nd^ generation catalyst resulted in the formation of compound **6** in 79% yield (Figure 3). Deprotection of compound **6** using HF·pyridine complex gave the C5′-chloromethyl derivative **ENT-A025**. Alternatively, by treating compound **6** with TBAF, the fluorinated congener of **ENT-A025** compound **ENT-A034** was obtained in quantitative yield (Figure 3).

Alkylation of compound **6** with para-methoxybenzyl alcohol in the presence of NaH resulted in the corresponding para-methoxybenzyl ether derivative **7** in 97% yield which enabled the obtainment of two derivatives, namely **ENT-A035** and **ENT-A008**. Deprotection of the 3β-hydroxy group of **7** using TBAF afforded **ENT-A035** in 48% yield. In turn, a reaction of **7** with 2,3-dichloro-5,6-dicyano-1,4-benzoquinone (DDQ), yielded the C5′-hydroxymethyl derivative **8** in 70% yield, which was then treated with HF·pyridine complex to afford **ENT-A008** in 73% yield (Figure 3).

**Figure 4:**
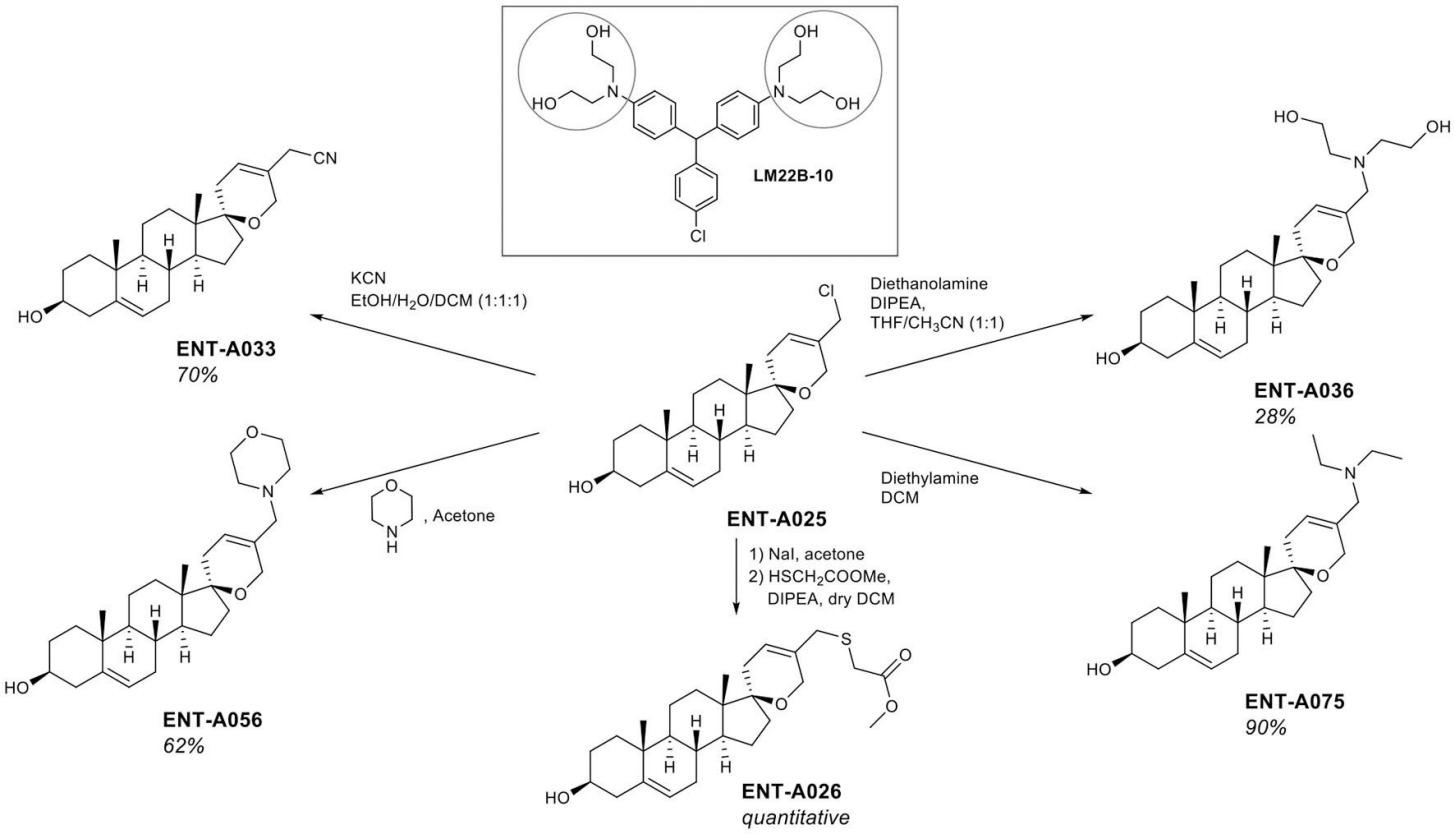
Synthesis of compounds **ENT-A026, ENT-A036**, **ENT-A056**, **ENT-A075**, and **ENT-A033** and structure of **LM22B-10**.

The thio analog **ENT-A026** was obtained in quantitative yield through a two-step reaction. Initially, the chloromethyl group in **ENT-A025** was transformed to iodomethyl using NaI in acetone that was reacted with methyl thioglycolate in dichloromethane to give **ENT-A026** (Figure 4). In our subsequent investigation, we incorporated nitrogen-bearing substituents at C5′. First, we successfully introduced a cyanomethyl group in 70% yield by stirring **ENT-A025** with KCN and catalytic KI. Then, the chloro group in **ENT-A025** was replaced by various dialkylamino groups. In particular, the reaction of **ENT-A025** with diethanolamine gave **ENT-A036** in 28% yield, while the reaction with morpholine afforded **ENT-A056** in 62% yield. Furthermore, the nucleophilic substitution of **ENT-A025** by diethylamine gave **ENT-A075** in 70% yield (Figure 4).

**Figure 5:**
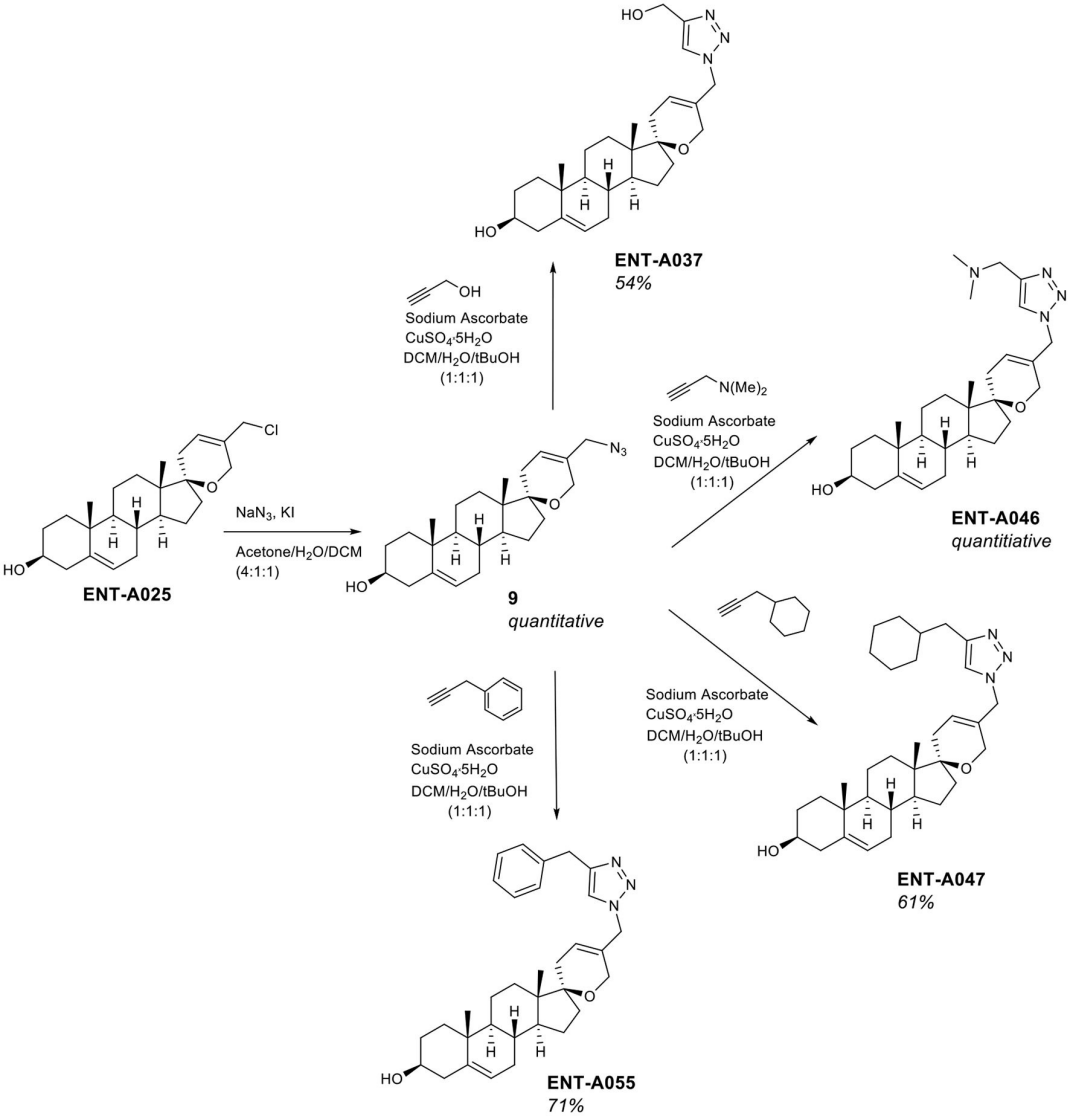
Synthesis of the 1,2,3-triazolyl-substituted derivatives **ENT-A037**, **ENT-A046**, **ENT-A047**, and **ENT-A055**.

To complement the nitrogen-containing derivatives, we introduced a 1,2,3-triazole moiety substituted by groups chosen for their ability to engage in hydrogen bonding or π-stacking interactions (Figure 5). Treatment of **ENT-A025** with NaN_3_ gave the azido derivative **9**. Click reaction of **9** with various substituted alkynes afforded the desired 1,2,3-triazolyl derivatives. Thus, the reaction of **9** with propargyl alcohol gave **ENT-A035** in 35% yield, with *N,N*-dimethyl-propargyl amine afforded **ENT-A046** in quantitative yield, with prop-2-yn-1-yl-cyclohexane yielded **ENT-A047** in 67% yield and with 3-phenyl-1-propyne analog **ENT-A055** in 71% yield (Figure 5).

**Figure 6:**
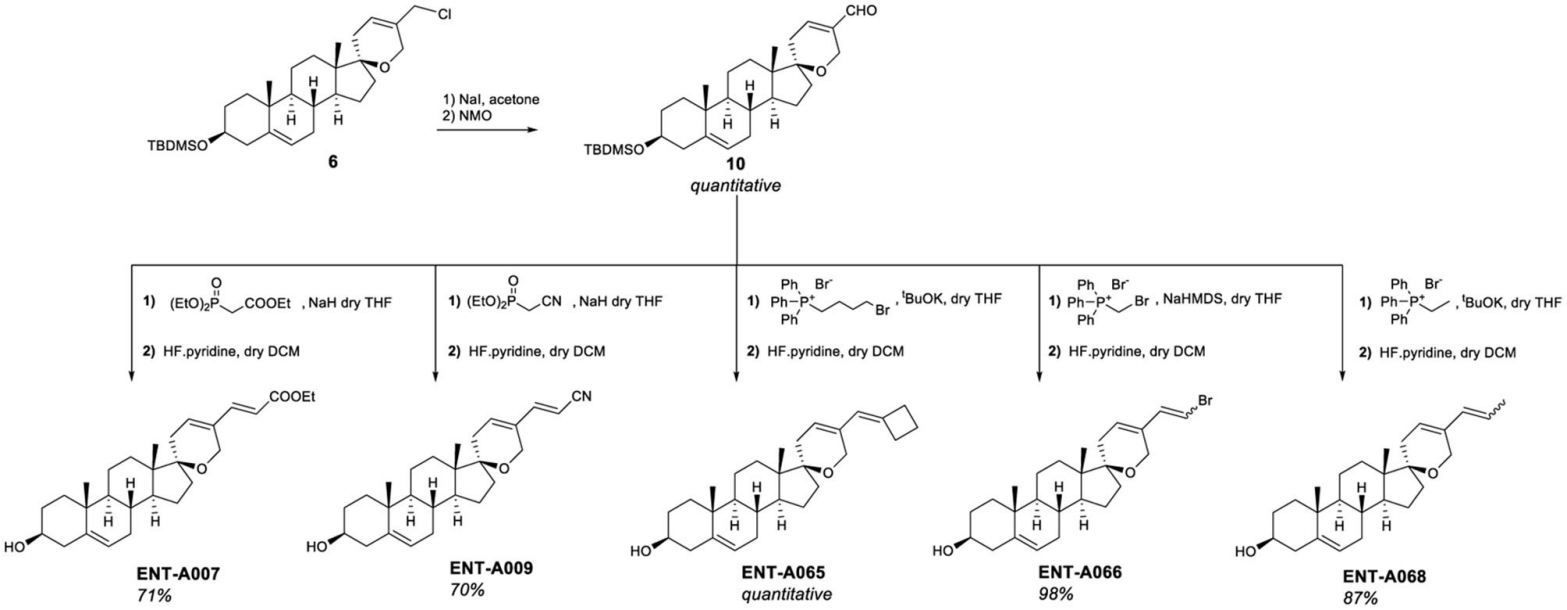
Synthesis of the unsaturated derivatives **ENT-A007**, **ENT-A009**, **ENT-A065**, **ENT-A066**, and **ENT-A068**.

To introduce unsaturated groups at C5′, the intermediate aldehyde derivative was required. Thus, compound **6** was fully converted to aldehyde **10** in a two-step one-pot reaction that involved the initial exchange of the chlorine atom with iodine, followed by oxidation using *N*-methyl-morpholine *N*-oxide (NMO) (Figure 6). Aldehyde **10** served as the starting material for generating a series of unsaturated derivatives through Horner-Emmons or Wittig reactions followed by deprotection of the C3-hydroxyl group (Figure 6). For the synthesis of **ENT-A007** and **ENT-A009**, aldehyde **10** was treated with NaH and triethyl phosphonoacetate or diethyl (cyanomethyl)-phosphonate, respectively. Furthermore, **ENT-A065** and **ENT-A068** were prepared from **10** by employing (4-bromobutyl)-triphenyl-phosphonium bromide or ethyl triphenyl-phosphonium bromide in the presence of potassium tert-butoxide (tBuOK) as the base, respectively. Similarly, the use of (bromomethyl)-triphenyl-phosphonium bromide with sodium bis(trimethylsilyl)amide (NaHMDS) led to the formation of the **ENT-A066** from **10**. The resulting C3-protected intermediates were directly subjected to deprotection with HF·pyridine complex to afford the corresponding final products **ENT-A007**, **ENT-A009**, **ENT-A065**, **ENT-A066**, and **ENT-A068** in 70 and 71% quantitative, 98 and 87% yield, respectively (Figure 6).

**Figure 7:**
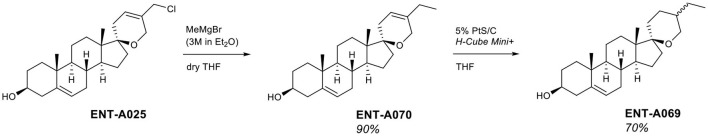
Synthesis of C5′-alkyl-substituted derivatives **ENT-A070** and **ENT-A069**.

Finally, our library of decorated C17-spiro-dihydropyran DHEA derivatives was complemented by two alkyl-substituted analogs. Grignard addition of methyl magnesium bromide to **ENT-A025** gave the C5′ ethyl-substituted analog **ENT-A070** in 90% yield. Finally, the double bond of the (2*H*)dihydropyran ring was selectively hydrogenated in **ENT-A070** in 70% yield, using a flow-through hydrogenator (H-Cube Mini Plus™) and 5% PtS/C as catalyst to afford **ENT-A069** (Figure 7).

#### 3.1.2. 17-spiro-cyclobutane derivatives

**Figure 8:**
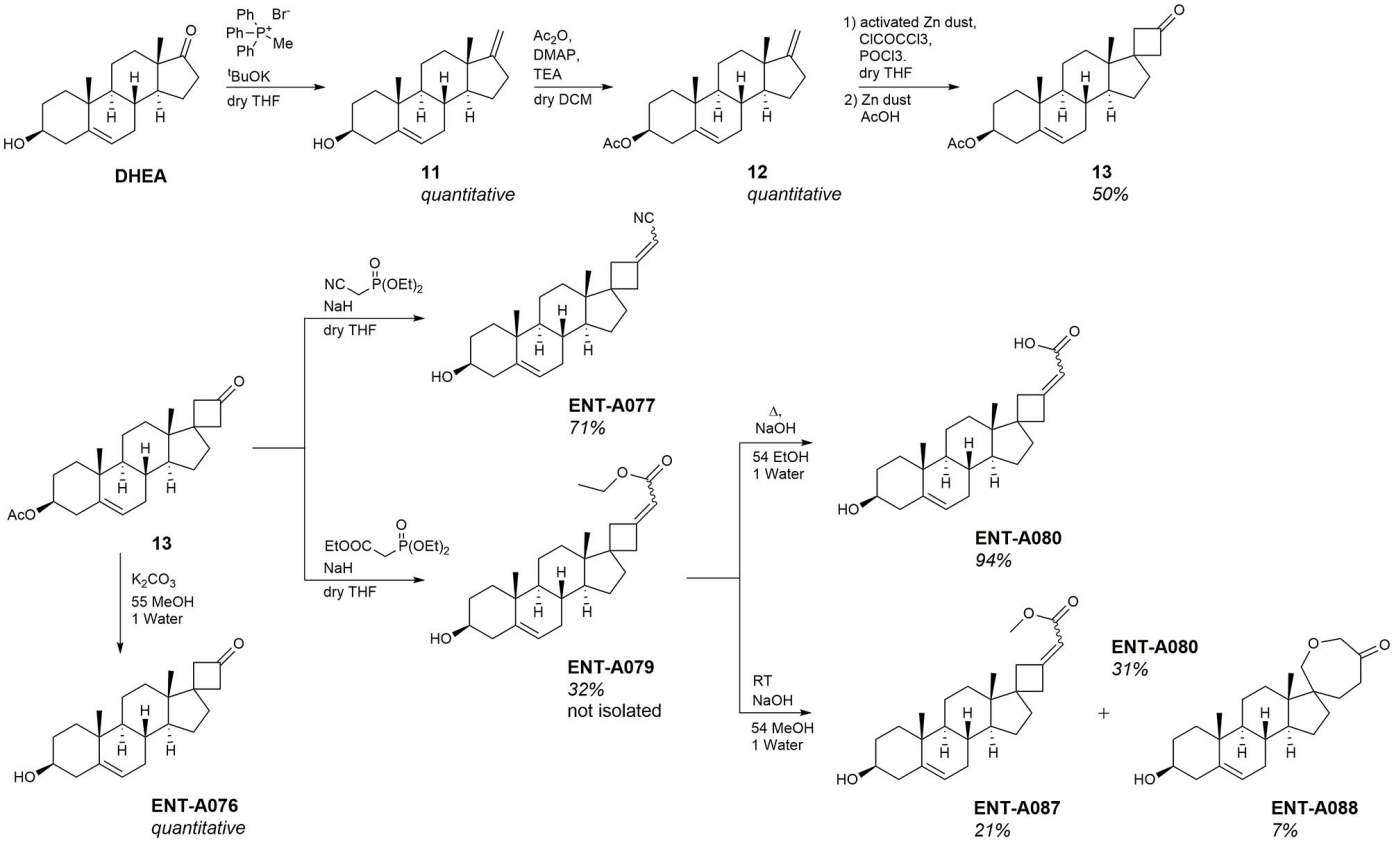
Synthesis of the key intermediate **13** and final compounds **ENT-A076**, **ENT-A077, ENT-A079, ENT-A080, ENT-A087**, and **ENT-A088**.

The second series of our study involves C17-spiro-cyclobutane DHEA derivatives (Figure 8). Initially, the C17-spiro-cyclobutanone DHEA analog was prepared using a reaction described by Zdzislaw and Blaszczyk (1990) (Figure 8). Wittig reaction of DHEA using methyltriphenylphosphonium bromide and tBuOK as base afforded quantitatively the C17-methylene derivative **11**. Acetylation of the C3-alcohol using acetic anhydride gave **12** in quantitative yield which was then subjected to a two-step reaction for the formation of the 17-spiro-cyclobutanone derivative **13**. This step proved to be particularly challenging, but we were able to increase the previously reported overall yield from 31.5 to 50% for the same substrate. The first step involved the dropwise addition of a solution of ClCOCCl_3_ and POCl_3_ to a mixture of activated zinc dust and compound **12**. Due to the instability of the resulting intermediate, the crude product was carried forward to the next step. Thus, the mixture was dissolved in acetic acid, supplemented with zinc dust, and was refluxed overnight to give **13** (Figure 8). Quantitative deprotection of the C3-alcohol in **13** was effected using potassium carbonate in methanol/water, affording **ENT-A076** (Figure 8). The Horner-Emmons reaction of **13** with cyanomethyl diethylphosphonate or carboxyethyl diethylphosphonate gave **ENT-A077** and **ENT-A079**, respectively (Figure 8). However, despite our extensive efforts, **ENT-A079** proved to be challenging. Not only it was obtained in low yield but also its purification was proven tedious. Thus, the ester group of **ENT-A079** was hydrolyzed using NaOH in different solvent mixtures to afford acid **ENT-A080** or the methyl ester **ENT-A087** or the rearrangement spiro-oxepan-3-one derivative **ENT-A088** (Figure 8). The respective yields were 94, 21, and 7%.

### 3.2. Screening of compounds that induce TrkA downstream kinases Akt and Erk1/2 activation and promote survival of PC12 cells

**Figure 9:**
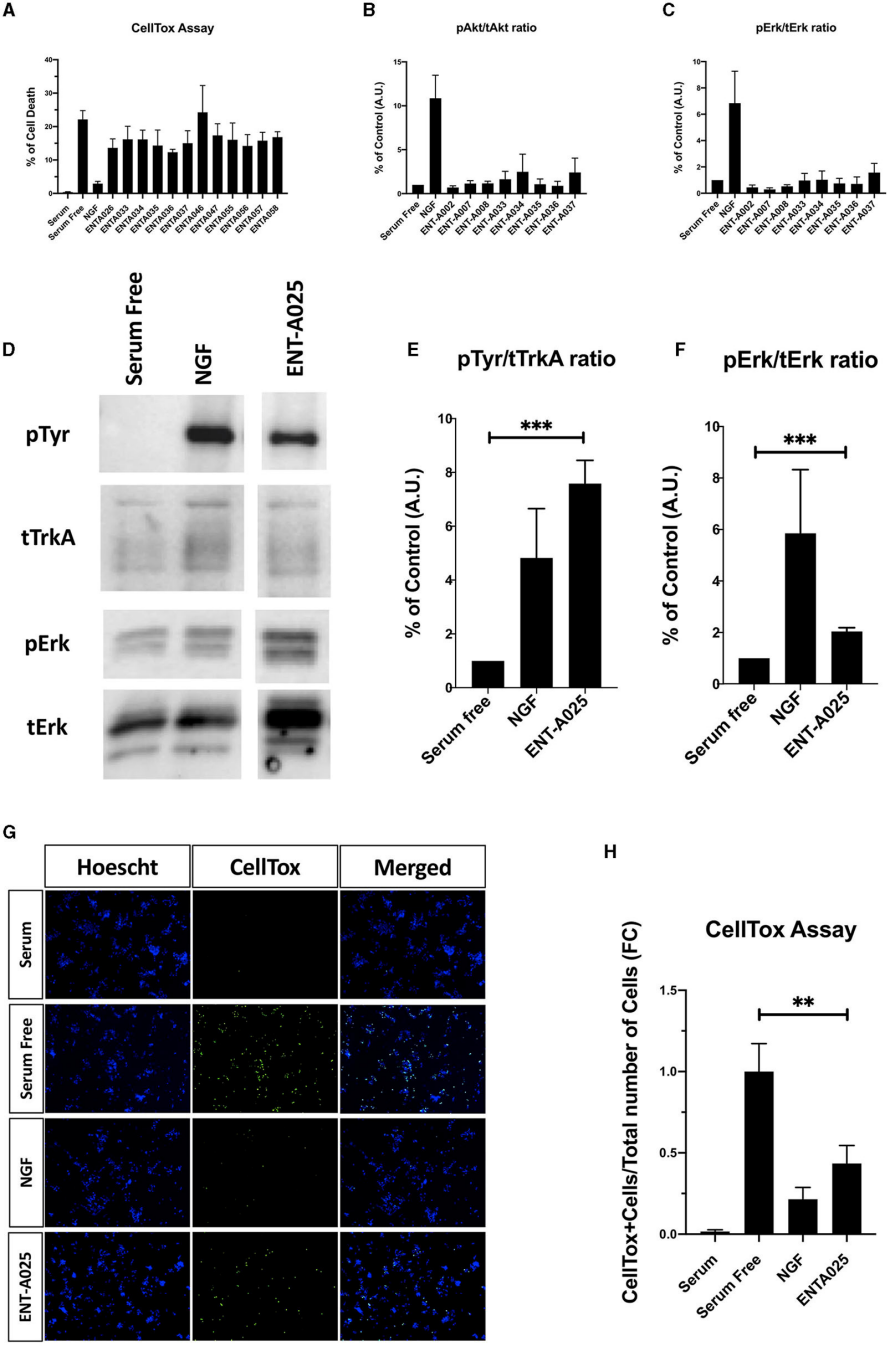
**(A–C)** Screening and identification of compounds for their ability to activate TrkA and its downstream signaling kinases as well as to promote PC12 cell survival. **(A)** Quantification of CellTox assay on PC12 cells starved from serum for 4 h and treated with compound (500 nM) or NGF (100 ng/mL) for 24 h, *n* = 3–6 independent experiments. **(B, C)** Quantification of phosphorylation of TrkA downstream signaling kinases Akt and ERK1/2 on PC12 cells treated with NGF (100 ng/mL) or compound (500 nM) for 30 min, *n* = 2–3 independent experiments for AKT, *n* = 3–4 independent experiments for ERK1/2. **(D–H) ENT-A025** strongly activates TrkA, its downstream signaling kinase ERK1/2, and protects PC12 cells from cell death. **(D–F)** Western Blot and quantification of TrkA and ERK1/2 phosphorylation on PC12 cells treated with **ENT-A025** (500 nM) or NGF (100 ng/mL). Data are shown as ±SEM, ***p < 0.001; Student's *t*-test against negative control, *n* = 4 independent experiments. **(G, H)** CellTox assay and quantification of PC12 cells starved from serum and treated with **ENT-A025** (500 nM) or NGF (100 ng/mL). Data are shown as ±SEM, **p < 0.01; One-way ANOVA, multiple comparisons, Turkey's test correction, *n* = 5 independent experiments.

**Figure 10:**
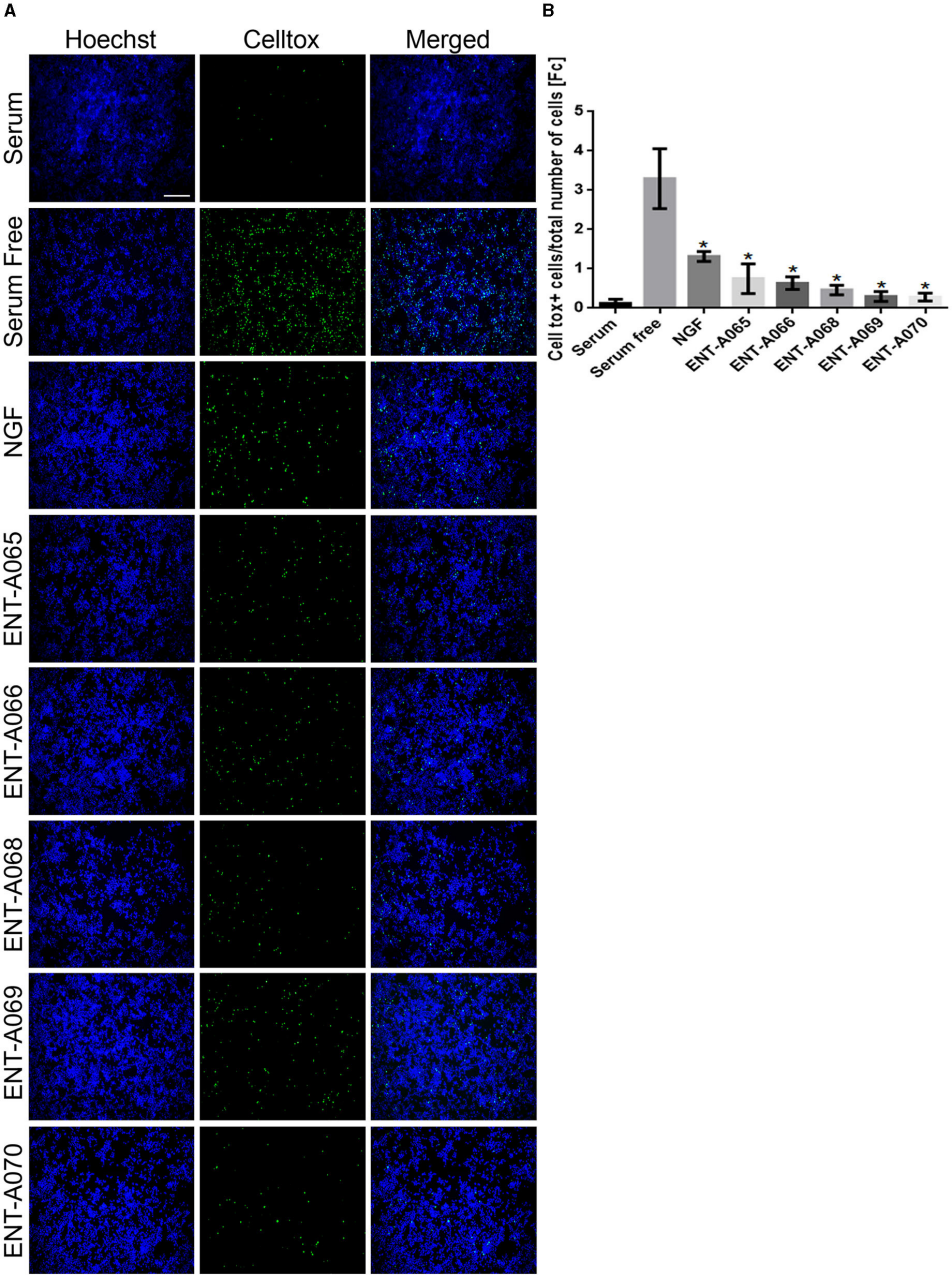
**ENT-A065, ENT-A066, ENT-A068, ENT-A069**, and **ENT-A070** protect PC12 cells from serum deprivation-induced death. **(A)** PC12 cells were starved of serum and treated with each compound (1 μM) or NGF (100 ng/mL). CellTox dye for dead cells (green) and Hoechst for the total number of cells (blue) were used. **(B)** Quantification of CellTox^+^ cells showed that compounds **ENT-A065, ENT-A066, ENT-A068, ENT-A069**, and **ENT-A070** promote cell survival as well as NGF does under stress conditions. Data are the average for 3 independent experiments, presented as mean ± SEM. Statistical analyses were performed by unpaired *t-*test; *p < 0.05.

Initially, we tested compounds **ENT-A002**, **ENT-A007, ENT-A009, ENTA025, ENT-A026, ENT-A033, ENT-A034, ENT-A035, ENT-A036, ENT-A037, ENT-A046, ENT-A047, ENT-A055**, and **ENT-A056** for their ability to protect PC12 cells from serum deprivation-induced cell death as well as for their ability to activate TrkA downstream pro-survival signaling kinases Akt and Erk1/2 (Figures 9A–C). We opted to employ PC12 cells, which express receptors TrkA and p75 and have been routinely used for investigating NGF-TrkA signaling. PC12 cells were subjected to a period of nutrient deprivation (without fetal bovine serum and horse serum in their culture medium) for 4 h. Following this, NGF (100 ng/mL) or one of the compounds (500 nM or 1 μM) was administered and allowed to interact with the cells for 24 h. Following that, CellTox and Hoescht dyes were added and cells were imaged to assess cell death. Control cases involved cells cultured in full medium (serum), cells under starvation conditions (serum-free), and cells under starvation conditions treated with DMSO (SF+DMSO). From this series, only the C5′-chloromethyl derivative **ENT-A025** was identified to strongly induce TrkA and Erk1/2 phosphorylation, comparable to NGF action (Figures 9D–F) and to protect PC12 cells against serum deprivation-induced cell death (Figures 9G, H). Subsequently, compounds **ENT-A065, ENT-A066, ENT-A068, ENT-A069**, and **ENT-A070** were evaluated, and derivatives **ENT-A066, ENT-A068, ENT-A069**, and **ENT-A070** were found to promote PC12 cell survival during stress conditions (Figures 10A, B).

### 3.3. Screening of compounds that promote survival of NIH-3T3-TrkB cells and induce phosphorylation/activation of TrkB

**Figure 11:**
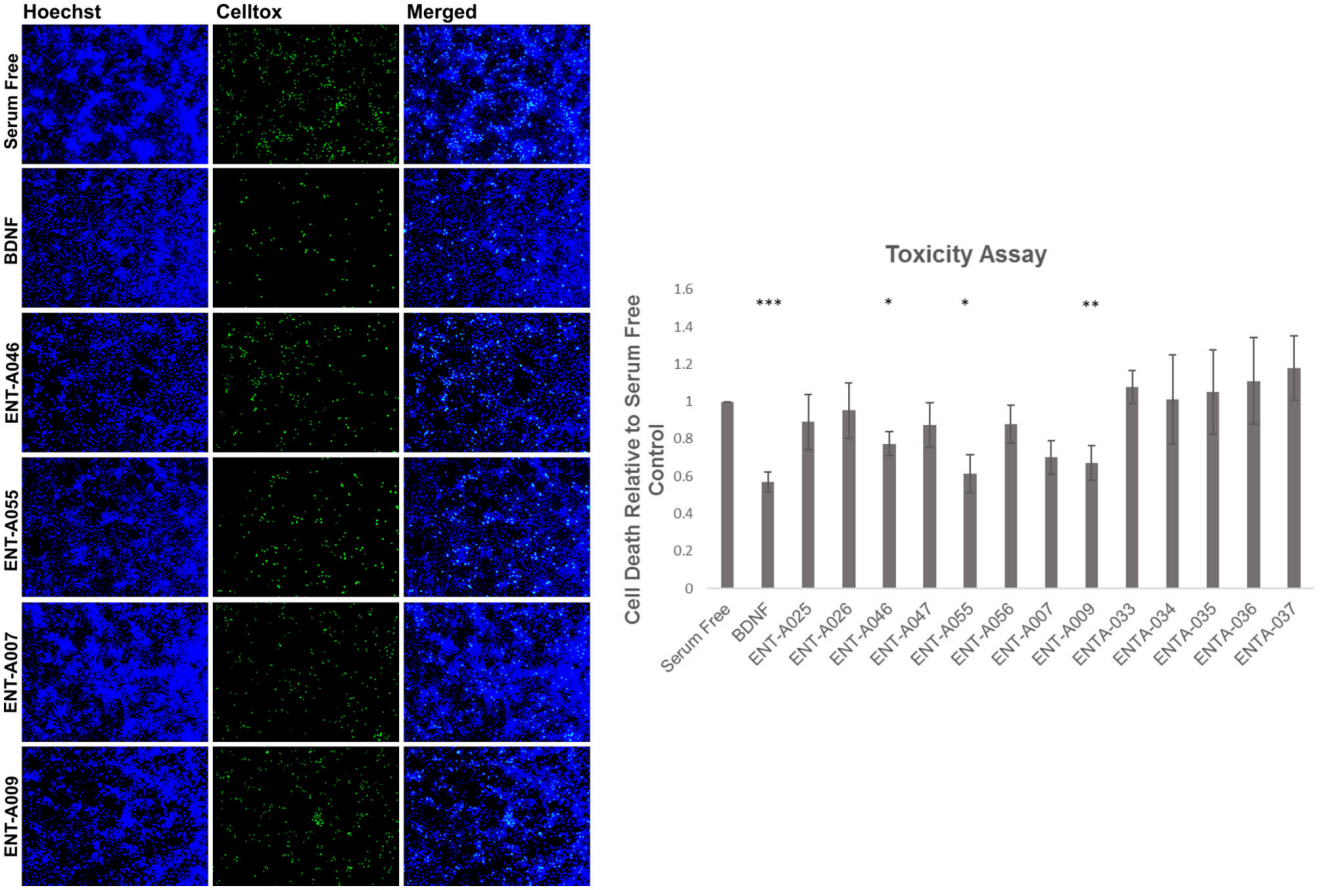
CellTox, Cell Toxicity Assay in the NIH-3T3 TrkB-expressing cell line. Quantification and representative images of NIH-3T3 cells, untreated and treated with BDNF (500 ng/mL) or compounds **ENT-A007**, **ENT-A009**, **ENT-A046**, and **ENT-A055** (1 μM) for 24 h, after 24 h of serum starvation, *n* = 3–5 independent experiments, error bars represent SEM. *p < 0.05, **p < 0.01, ***p < 0.001.

The NIH-3T3 TrkB cell line, which is stable and transfected to specifically express the TrkB receptor, was used for the screening of the new compounds for their ability to protect cells from serum deprivation-induced apoptosis through the activation of the TrkB receptor. Cell death was induced by serum deprivation for 24 h and then BDNF (500 ng/mL), the native neurotrophin ligand of the TrkB receptor, or compounds **ENT-A007, ENT-A009, ENTA025, ENT-A026, ENT-A033, ENT-A034, ENT-A035, ENT-A036, ENT-A037, ENT-A046, ENT-A047, ENT-A055**, and **ENT-A056** (1 μM) were added to the media for 24 h. The compounds **ENT-A007**, **ENT-A009**, **ENT-A046**, and **ENT-A055** showed the ability to significantly reduce cell death levels compared to the serum-free control, comparable to the BDNF action (Figure 11).

**Figure 12:**
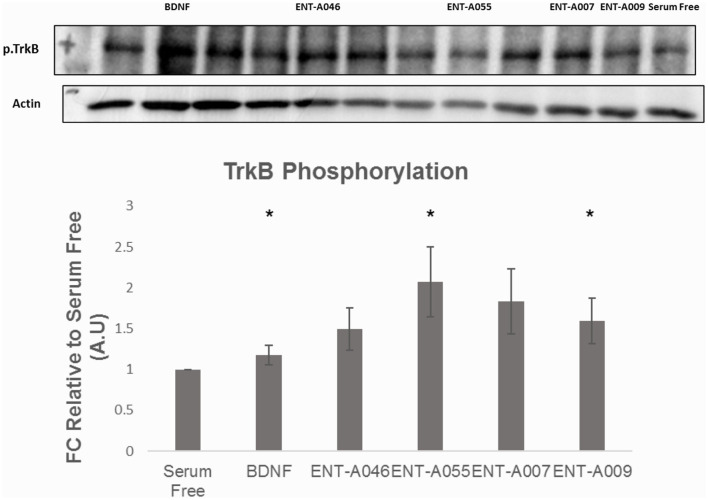
Phosphorylation assay in primary astrocytes. Quantification and representative images [**(top)**, phospho-TrkB, and β-Actin] for the Western Blot Assay, *n* = 3 independent experiments. Cells were treated with BDNF (500 ng/mL) or compounds (1 μM) for 20 min, after 6 h of serum starvation. Error bars represent SEM. *p < 0.05.

Subsequently, the compounds that protected NIH3T3-TrkB cells from cell death were evaluated for their ability to phosphorylate TrkB. Mouse astrocytes were employed as a primary neuronal population that endogenously expresses TrkB, to assess more physiologically if the compounds have similar effects in a primary cell line that is dependent on TrkB signaling. Among derivatives **ENT-A007**, **ENT-A009**, **ENT-A046**, and **ENT-A055** that showed cell protection, only **ENT-A009** and **ENT-A055** successfully increased phosphorylation levels of the TrkB receptor compared to the untreated control (Figure 12), in a comparable manner to BDNF.

**Figure 13:**
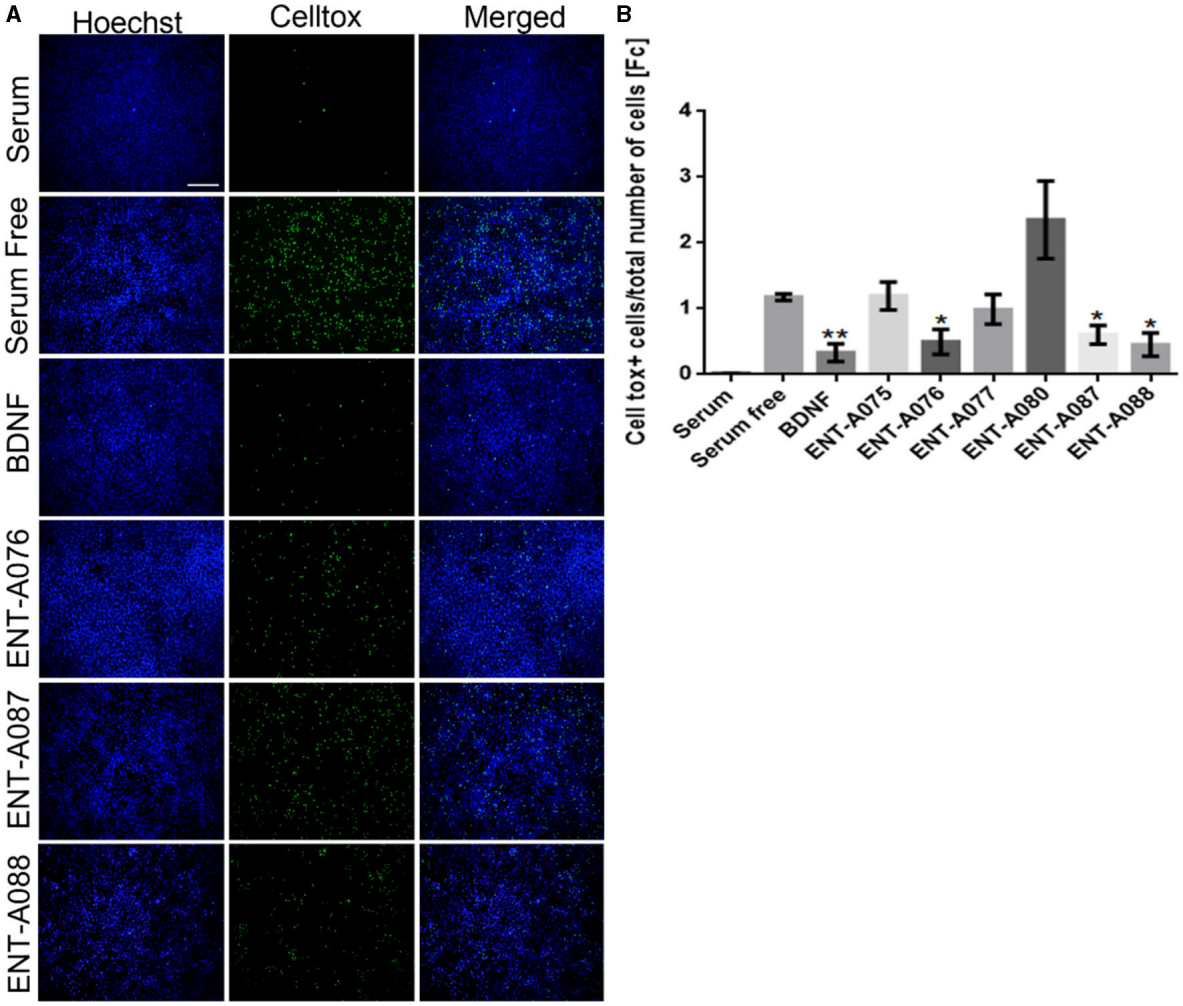
**ENT-A076, ENT-A087**, and **ENT-A088** protect NIH3T3 TrkB stable transfected cells from serum deprivation-induced death. **(A)** NIH3T3 TrkB stable transfected cells were starved of serum and treated with each compound (1 μM) or BDNF (500 ng/mL). CellTox dye for dead cells (green) and Hoechst for the total number of cells (blue) were used. **(B)** Quantification of CellTox^+^ cells showed that compounds **ENT-A076, ENT-A087**, and **ENT-A088** promote cell survival as well as BDNF under stress conditions. Data are the average for 3 independent experiments, presented as mean ± SEM. Statistical analyses were performed by unpaired t-test; *p < 0.05; **p < 0.01.

Subsequently, compounds **ENT-A075, ENT-A076, ENT-A077, ENT-A080, ENT-A087**, and **ENT-A088** were initially screened in the NIH3T3-TrkB stable transfected cells for their ability to promote cell survival under serum deprivation conditions as described above. Based on our findings, we can conclude that compounds **ENT-A076, ENT-A087**, and **ENT-A088** demonstrated the ability to promote cell survival during serum starvation as well as BDNF (Figure 13).

**Figure 14:**
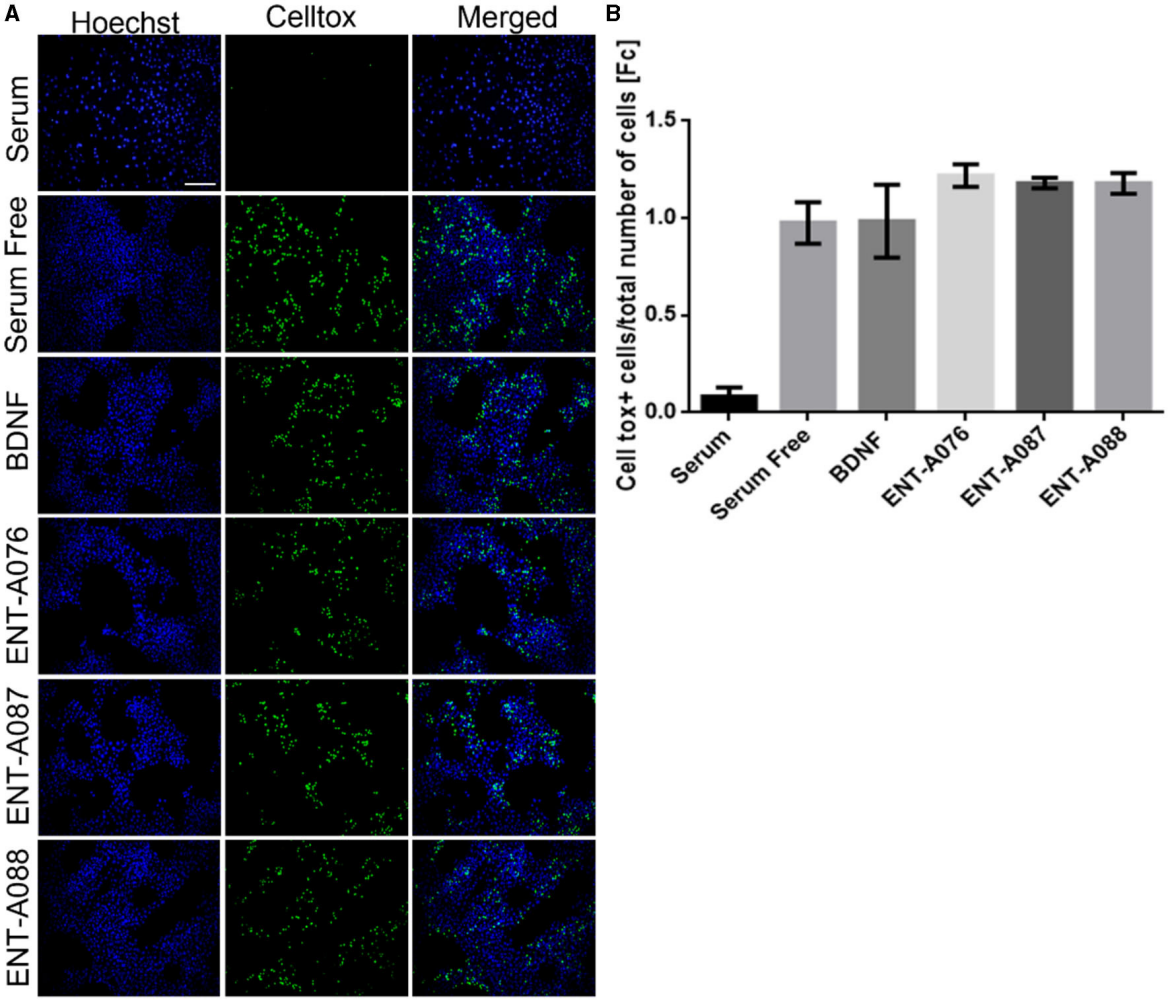
TrkB receptor is involved in the cell-protective effects of compounds **ENT-A076, ENT-A087**, and **ENT-A088**. **(A)** NIH3T3 naive cells were starved of serum and treated with each compound (1 μM) or BDNF (500 ng/mL). CellTox dye for dead cells (green) and Hoechst for the total number of cells (blue) were used. **(B)** Quantification of CellTox+ cells showed that neither BDNF nor compound administration was not capable of saving cells from death. Thus, we can assume that the cell survival effect is exclusively mediated through the activation of the TrkB receptor. Data are the average for 3 independent experiments, presented as mean ± SEM. Statistical analysis was performed by unpaired *t*-test.

To confirm the involvement of the TrkB receptor in the observed cell survival effects by compounds **ENT-A076**, **ENT-A087**, and **ENT-A088**, NIH3T3 cells that do not naturally express any neurotrophin receptors were employed. These cells were treated with the compounds, or BDNF, for 24 h under starvation conditions. Control conditions included cells cultured with serum, serum-free conditions, and serum-free conditions supplemented with DMSO. The cells were subjected to a period of 18 h of nutrient deprivation before the treatments. However, neither the compounds nor BDNF were able to rescue the cells from cell death. Therefore, we infer that the involvement of the TrkB receptor is indeed essential for the cell-protective effects of **ENT-A076**, **ENT-A087**, and **ENT-A088** (Figures 14A, B).

### 3.4. The new compounds possess drug-like properties

The predicted physicochemical profile and drug-like properties of the compounds were satisfactory, displaying in general values within the 95% range of the known drugs except the limited aqueous solubility for a number of compounds. Moreover, the compounds **ENT-A035, ENT-A0047, ENT-A065, and ENT-A055** exceed the lipophilicity range of values with potential impact in their distribution. The number of potential metabolites emerged slightly increased for the compounds **ENT-A035, ENT-A036, ENT-A046, and ENT-A056**. Regarding the potential reactivity of the compounds, the presence of unhindered ester functionality as in the compounds **ENT-A026** and **ENT-A088** or the carbonyl group in the 4-ring system has been highlighted (Supplementary Table S1).

### 3.5. Test compounds are metabolically stable in human liver microsomes

The susceptibility of a test compound to biotransformation is defined as metabolic stability. For the ranking of test compounds, several approaches can be followed including parent structure loss during metabolic reactions or their intrinsic clearance (CL_int_) and *in vitro* half-life (t_1/2_) values (Masimirembwa et al., 2003; Barter et al., 2007). Herein, we chose the former.

Parent structure loss is classified as very slow (<5%), slow (5–19%), moderate (20–50%), fast (50–80%), or very fast (>80%). Such categories have been defined according to set criteria, namely, high metabolism (t_1/2_ value of <30 min), moderate metabolism (30 min <t_1/2_ value of <60 min), and low metabolism (t_1/2_ value of >60 min) (EMA, 2012, 2013; FDA, 2020).

**Figure 15:**
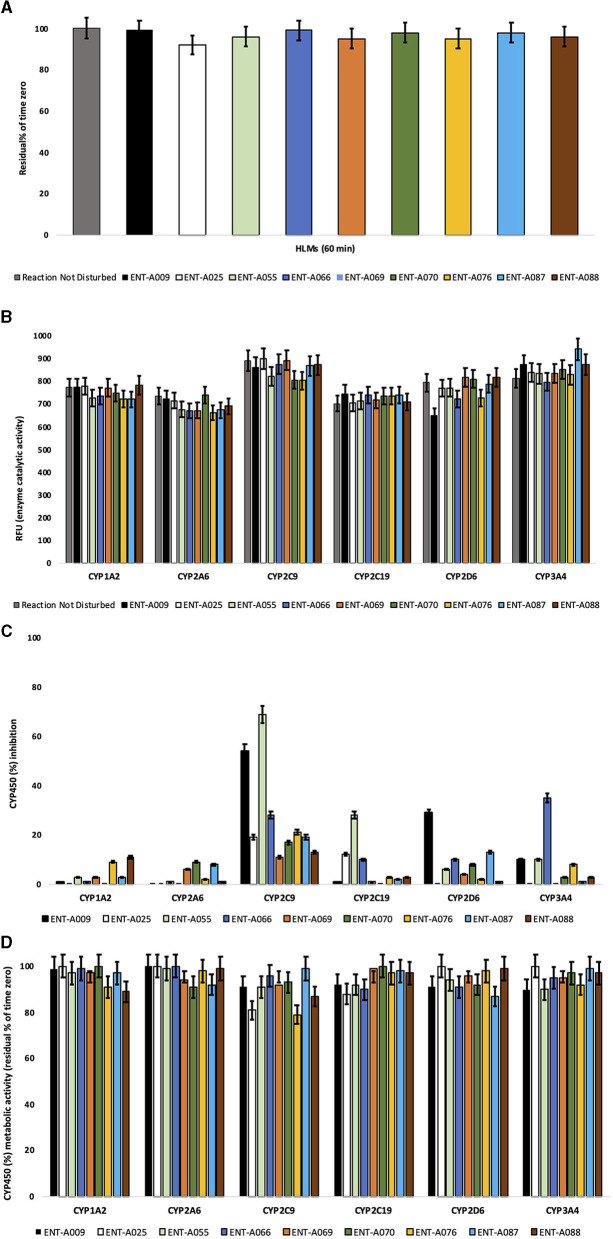
**(A)** Microsomal stability of test compounds at 1 μM upon incubation with pooled human liver microsomes (60 min). **(B)** Enzyme (catalytic) activity of the CYP1A2, CYP2A6, CYP2C9, CYP2C19, CYP2D6, and CYP3A4 isoenzymes upon the administration of test compounds at 1 μM (60 min). RFU, relative fluorescence units. Reaction Not Disturbed, reaction without test compounds. **(C)** CYP450 (%) inhibition of CYP1A2, CYP2A6, CYP2C9, CYP2C19, CYP2D6, and CYP3A4 isoenzymes upon the administration of test compounds at 1 μM (60 min). **(D)** CYP450 (%) metabolic activity of CYP1A2, CYP2A6, CYP2C9, CYP2C19, CYP2D6, and CYP3A4 isoenzymes upon the administration of test compounds at 1 μM (60 min).

**ENT-A055, ENT-A058, ENT-A066, ENT-A070, ENT-A087**, and **ENT-A088** were very slowly depleted showing >95% residual of time zero at t = 60 min, except for **ENT-A025** (92%), **ENT-A069** (95%), and **ENT-A076** (95%) that exhibit slow depletion (Figure 15A). Thus, all test compounds may correspond to low or medium intrinsic clearance classification bands.

### 3.6. Test compounds exhibit slow diverse isozyme-specific CYP450 metabolism

Human CYP450 enzymes are crucial for xenobiotic biodegradation, metabolism, and toxicity as well as xenobiotic–host and/or xenobiotic–xenobiotic interactions (McGinnity et al., 2000). Upon linear velocity conditions *in vitro*, the depletion rate of test compounds may be extrapolated to (a) *in vivo* hepatic clearance, (b) extraction ratio, and (c) the effect of hepatic first-pass metabolism on total oral bioavailability. Biodegradation, metabolic, and toxicity liabilities can be identified early on and thus inform structure–activity relationships (SAR) (EMA, 2012, 2013; FDA, 2020).

For this, the activity of CYP1A2, CYP2A6, CYP2C9, CYP2C19, CYP2D6, and CYP3A4 was assessed after the administration of test compounds at 1 μM to determine (a) the oxidative (CYP-mediated) metabolic stability profile in question and (b) the enzyme metabolizing isoforms responsible (the test system consists of recombinant human CYP450 and CYP450 reductase; cytochrome b_5_ may also be present). Herein, no concentration-dependent effects were reported. CYP450 substrates can alter enzyme activity by blocking the enzyme's active site, changing enzyme conformation, and disrupting enzyme structure and/or functioning (Pelkonen and Turpeinen, 2007; Zanger and Schwab, 2013). No test compound decreased the enzyme (catalytic) activity of the CYP450 system tested herein. CYP450 enzyme inhibition may lead to unexpectedly high exposure to co-administered xenobiotics and hence increase the risk for adverse effects. No product inhibition or mechanism-based inactivation of the CYP450 isoenzymes in question was obtained. Test compounds with poor solubility can show artificially low CYP450 inhibition and thus, chemical entities with potential drug–drug interaction toxicities may be overlooked. No solubility issues were observed. All test compounds were weak or moderate inhibitors of the CYP450 system. **ENT-A055** and **ENT-A009** showed moderate-to-strong inhibition for CYP2C9. Findings are summarized in Figures 15B–D.

## 4. Discussion

Neurotrophins belong to a family of secreted proteins, widely expressed in the peripheral and the central nervous system, that majorly support neuronal survival, synaptic plasticity, and neurogenesis. The predominant neurotrophins in adult humans are nerve growth factor (NGF) and brain-derived neurotrophic factor (BDNF) acting through their high-affinity receptors: TrkA and TrkB, respectively. Their fine regulation governs fundamental functions of the brain, such as memory and learning. Alterations of BDNF/TrkB signaling in the cortical and hippocampal area, in fact, were indicated as a hallmark of numerous neurodegenerative diseases, and a Trk agonist-mediated restoration holds the promise of a disease-modifying treatment (reviewed by Longo and Massa, 2013).

In accordance with the unmet need for neurotrophin receptor manipulation, we have now synthesized a library of 27 new C17-spiro DHEA derivatives in a quest to decipher the stereo-electronic requirements for selective activation of neurotrophin receptors TrkA or TrkB. Building on the discovery of the prototype BNN27, a selective TrkA agonist (Calogeropoulou et al., 2009), we expanded our seminal results with two series of derivatives substituted at C17 by six- or four-membered spirocyclic moieties. All the reported compounds showed increased metabolic stability as a result of the ring expansion of BNN27′s C17-spiro-oxirane moiety. The first series encompasses C17-spiro-dihydro(*2H*)pyran derivatives decorated at C5′ by a variety of pharmacophoric groups. In particular, our in-depth investigations began with the synthesis of the C5′-unsubstituted analog **ENT-A002**. Evaluation of the antiapoptotic activity in PC12 and NIH3T3-TrkB cells did not show any protective effects. Thus, we embarked on decorating the C5′ position of **ENT-A002** with different substituents aiming to restore the neuroprotective effect of BNN27. Thus, we initially introduced an electrophilic handle via a chloromethyl substituent (compound **ENT-A025**) that in turn could enable the further derivatization to multiple analogs, thus expanding the diversity of our compound library. Gratifyingly, **ENT-A025** strongly activates TrkA and its downstream signaling kinase ERK1/2 while protecting PC12 cells from cell death (Figure 9). Conversely, it cannot protect NIH3T3-TrkB cells from serum deprivation (Figure 11). Thus, the previous findings suggest **ENT-A025** is a selective TrkA agonist, capable of mimicking some of the actions of NGF. Aiming to map the structural requirements for neurotrophin mimetic activity, we further derivatized **ENT-A025** at the C5′ position and generated its fluoro congener compound **ENT-A034**, the hydroxymethyl analog **ENT-A008**, the (*p-*methoxybenzyloxy)methyl derivative compound **ENT-A035**, the C5′-methyl(thiomethylacetate)-substituted analog **ENT-A026**, and the cyanomethyl derivative **ENT-A033**. Unfortunately, none of these compounds provided any protective effects in PC12 (Figure 9) or NIH3T3-TrkB cells (data not shown). Interestingly, **ENT-A008** bears the hydroxymethyl substituent of our prototype compound BNN27, indicating that the larger six-membered C17-spiro substituent compromised the activity. Continuing our structure–activity studies, we designed a small group of analogs borrowing structural features from the neurotrophin mimetic compounds reported by the groups of Longo and Massa (2013), in particular, **LM22B-10** (Yang et al., 2016). This compound, characterized by three diethanolamino moieties, binds and activates TrkB and TrkC receptors to promote cell survival and induce neurite outgrowth. Thus, we incorporated the diethanolamino functionality on the C5′ position of **ENT-A025**, resulting in **ENT-A036** (Scheme 4). Inspired by the potential of this pharmacophore, we envisaged two additional analogs modulating the nature of the amino group. In the first (**ENT-A075**), the two alcohol groups were replaced by hydrogens, and in the second (**ENT-A056**), they were etherized into a morpholine. Interestingly, only the diethylamino derivative **ENT-A075** showed potent antiapoptotic activity in NIH3T3-TrkB cells (Figure 13). Subsequently, we introduced the well-known five-membered heterocyclic 1,2,3-triazole group at C5′ of **ENT-A025**. This pharmacophore moiety is considered an amide group bioisostere and has been employed in hit-to-lead optimization campaigns resulting in bioactive compounds encompassing an array of pharmacological actions (Kumar and Kavitha, 2013; Bonandi et al., 2017). Four 1,2,3-triazolyl derivatives were prepared substituted by hydroxymethyl (**ENT-A037**), *N,N*-dimethylaminomethyl (**ENT-A046**), or cyclohexylmethyl (**ENT-A047**) or benzyl (**ENT-A055**) groups on the heterocyclic ring. These carefully selected substituents could enable potential hydrogen bonding, π-stacking, or hydrophobic interactions with the neurotrophin receptors. We were very excited to identify **ENT-A046** and **ENT-A055** capable of protecting NIH3T3-TrkB cells from serum deprivation comparably to the natural ligand BDNF (Figure 11) without showing any antiapoptotic activity in PC12 cells (Figure 9A). However, when tested for their ability to phosphorylate TrkB in mouse astrocytes, only the benzyl-substituted 1,2,3-triazole containing derivative **ENT-A055** successfully increased phosphorylation levels of the TrkB receptor compared to the untreated control (Figure 12). Thus, **ENT-A055** is a selective TrkB agonist mimicking some of the protective effects of BDNF. These differential effects by **ENT-A046** and **ENT-A055** could reflect structurally induced differences in various signaling pathways and cellular phenotypes between the two molecules. The (*2H*)dihydropyran series was finally complemented by a group of compounds bearing a vinyl group at C5′. Compounds **ENT-A007** and **ENT-A009** were substituted by α,β-unsaturated moieties as nucleophilic warheads, **ENT-A066** a bromovinyl substituent, while **ENT-A065** and **ENT-A068** as olefinic groups (cyclobutylmethylene or allyl, respectively). From this group, compounds **ENT-A065, ENT-A066**, and **ENT-A068** were found to promote PC12 cell survival as well as NGF under stress conditions (Figure 10). In contrast, compound **ENT-A009** was found to be a selective TrkB agonist, protecting NIH3T3-TrkB cells from cell death (Figure 11) and inducing TrkB phosphorylation comparably to BDNF (Figure 12). Interestingly, derivative **ENT-A066** bears a C5′-bromovinyl substituent that is also present in our previously reported selective TrkA agonist **ENT-A013** which mimics NGF activity in cell lines and primary neuronal populations and exhibits neuroprotective and anti-amyloid properties (Rogdakis et al., 2022). Thus, this strongly indicates that the bromovinyl moiety confers TrkA selectivity, irrespective of the size of the C17-spirocyclic moiety on the DHEA steroidal scaffold. Since compound **ENT-A068** was a potent TrkA agonist, we synthesized two saturated derivatives replacing the chloro substituent in **ENT-A025** with a methyl group and subsequently reducing the endocyclic double bond of the (2*H*)dihydropyran group (compounds **ENT-A070** and **ENT-A069**, respectively). This would enable us to identify the contribution of the unsaturations on the neurotrophin mimetic activity. Both derivatives were able to protect PC12 cells from serum deprivation-induced death, albeit the (2*H*)-dihydropyran derivative (**ENT-A070**) being more potent (Figure 10). Thus, the presence of the endocyclic double bond is beneficial for the NGF mimetic activity.

Based on our previous results (Yilmaz et al., 2022) that we identified compound **ENT-A010** bearing a cyclopropyl group conjugated to an α,β-unsaturated ester as a dual agonist of TrkA and TrkB receptors, we set out to prepare a small series of cyclobutyl derivatives to clarify the optimum size of the C17-spirocyclic moiety. Thus, the C17-cyclobutanone analog (**ENT-A076**), the α,β-unsaturated cyano (**ENT-A077**), α,β-unsaturated ethyl ester (**ENT-A079**), α,β-unsaturated methyl ester (**ENT-A087**), and α,β-unsaturated acid (**ENT-A080**) derivatives were prepared. As a side product during the synthesis of **ENT-A087**, the spiro-oxepan-3-one derivative **ENT-A088** was formed via a rearrangement reaction. Excitingly, **ENT-A076**, **ENT-A087**, and **ENT-A088** were shown to possess antiapoptotic activity in NIH-3T3-TrkB cells exclusively mediated through the TrkB receptor (Figures 13, 14). To get an insight into the drug-likeness of the new compounds, we predicted the physicochemical profile of the new synthetic compounds and were found to display values within the 95% range of the known drugs. As a drawback, a number of derivatives were predicted to have limited aqueous solubility. Finally, we performed an isozyme-specific CYP450 study to delineate the interactions among the active compounds with human CYP450 isoenzymes that account for most xenobiotic biodegradation, metabolism, and toxicity, as well as xenobiotic–xenobiotic and/or xenobiotic–host interactions. All the tested compounds were weak or moderate inhibitors of the CYP450 system. However, **ENT-A007** and **ENT-A009** showed moderate-to-strong inhibition for CYP2C9. Furthermore, none of the compounds tested showed biodegradation or liver metabolism (≥95% residual of time zero at t = 60 min) or safety issues.

## 5. Conclusion

In conclusion, this study focused on designing and synthesizing novel small neurotrophin mimetics to target neurotrophin receptors and combat neurodegeneration. A total of two series of DHEA derivatives, comprising 27 compounds, were synthesized and evaluated *in vitro* for their ability to stimulate survival in neurotoxicity models involving TrkA or TrkB receptors.

Among the compounds tested, **ENT-A025** strongly induces TrkA and Erk1/2 phosphorylation, comparable to NGF, and can protect PC12 cells against serum deprivation-induced cell death. Furthermore, compounds **ENT-A065, ENT-A066**, **ENT-A068**, **ENT-A069**, and **ENT-A070** have shown promising pro-survival effects in the PC12 cell line. These C17-spiro-dihydropyran derivatives will provide valuable insights into the structure–activity relationship (SAR) and identification of the most potent steroidal-based derivative for TrkA activation. In contrast, the SAR of the spiro-dihydropyran compounds showing pro-survival effects on TrkB is more complex. Compound **ENT-A009**, bearing a C5′-α,β-unsaturated cyano moiety, **ENT-A075** with a C5′-diethylaminomethyl group, and **ENT-A055** featuring a larger decoration by a benzyl-substituted 1,2,3-triazole group at C5′ position suggest a higher tolerability for the TrkB binding site(s) for these mimetics. Interestingly, the C17-spirocyclobutyl derivatives **ENT-A076** and **ENT-A087** exhibited potent antiapoptotic activity mediated through TrkB despite their smaller spiro-ring and decorations. Lastly, **ENT-A088** holds promise in exploring even larger spirocyclic moieties for TrkB receptor activation.

Overall, these findings provide valuable insights into the optimal stereo-electronic requirements for neurotrophin receptor agonism, offering potential avenues for mapping and dissecting the structural properties of neurotrophin receptors with their agonists and thus selectively control their signaling, to develop effective therapeutic interventions against neurodegenerative diseases.

## Data availability statement

The original contributions presented in the study are included in the article/Supplementary material, further inquiries can be directed to the corresponding author.

## Ethics statement

Ethical approval was not required for the studies on animals in accordance with the local legislation and institutional requirements because only commercially available established cell lines were used.

## Author contributions

TC, IC, AG, and KP designed and supervised the study. DN performed the synthesis and characterization of the new compounds. DC, TR, and IZ performed the screening experiments. TK and VB performed the *in vitro* ADMET studies. MZ performed the prediction of the drug-like properties of the new compounds. DN, DC, TR, IZ, VB, TK, AG, KP, IC, and TC interpreted the data and wrote the manuscript. All authors have read and approved the manuscript.
