# Supplementary material for: A quest for the stereo-electronic requirements for selective agonism for the neurotrophin receptors TrkA and TrkB in 17-spirocyclic-dehydroepiandrosterone derivatives
Source: Front Mol Neurosci. 2023 Sep 28;16:1244133. doi: 10.3389/fnmol.2023.1244133 (PMC10568017; doi:10.3389/fnmol.2023.1244133)
Supplement: Supplementary file 1 [file Data_Sheet_1.docx]

Supplementary Material

A quest for the stereo-electronic requirements for selective agonism for the neurotrophin receptors TrkA and TrkB in 17-spirocyclic-dehydroepiandrosterone derivatives.

**Daniele Narducci^1^, Despoina Charou^2,3^, Thanasis Rogdakis^2,3^, Ioanna Zota^2,3^, Vivi Bafiti^1^, Maria Zervou^1^, Theodora Katsila^1^, Achille Gravanis^2,3^, Kyriakos C. Prousis^1^, Ioannis Charalampopoulos^2,3^, Theodora Calogeropoulou^1*^**

*** Correspondence:** Theodora Calogeropoulou: tcalog@eie.gr

**Table S1**: Representative descriptors and predicted properties of the compounds. In parenthesis the range of values corresponding to 95% of known drugs. Values exceeding those ranges are reported in bold.

|  | **ENT-A002** | **ENT-A007** | **ENT-A008** | **ENT-A009** | **ENT-A025** | **ENT-A026** | **ENT-A033** | **ENT-A034** | **ENT-A035** | **ENT-A036** | **ENT-A036 (protonated tertiary amine at pH=7.0±0.5)** | **ENT-A037** | **ENT-A046** | **ENT-A046 (protonated tertiary amine at pH=7.0±0.5)** | **ENT-A047** |
| --- | --- | --- | --- | --- | --- | --- | --- | --- | --- | --- | --- | --- | --- | --- | --- |
| MW | 342.52 | 440.62 | 372.55 | 393.57 | 390.99 | 460.67 | 381.56 | 374.54 | 492.70 | 459.67 | 459.67 | 453.63 | 480.70 | 480.70 | 519.77 |
| Donor HB (0.0 / 6.0) | 1.000 | 1.000 | 2.000 | 1.000 | 1.000 | 1.000 | 1.000 | 1.000 | 1.000 | 3.000 | 3.000 | 2.000 | 1.000 | 1.000 | 1.000 |
| Accpt HB (2.0 / 20.0) | 2.450 | 4.450 | 4.150 | 3.950 | 2.450 | 4.950 | 3.950 | 2.450 | 4.900 | 7.850 | 7.850 | 6.650 | 6.950 | 6.950 | 4.950 |
| No. of Primary Metabolites (1.0 / 8.0) | 6 | 6 | 7 | 6 | 7 | 8 | 7 | 7 | **10** | **10** | **10** | 8 | **9** | **9** | 8 |
| No. of reactive groups | 0 | 1^a^ | 0 | 0 | 0 | 1^b^ | 0 | 0 | 0 | 0 | 0 | 0 | 0 | 0 | 0 |
| logPo/w^1^ (-2.0 / 6.5) | 5,063 | 5,886 | 4.271 | 4,961 | 5.909 | 5.818 | 4.690 | 5.622 | **7.094** | 3.535 | 3.628 | 4.324 | 5.167 | 5.033 | **7.314** |
| logS^2^ (-6.5 / 0.5) | -5,97 | **-8,037** | -5.649 | **-7,558** | **-7.233** | **-7.726** | **-7.277** | **-6.828** | **-8.619** | -4.507 | -4.344 | -6.438 | **-7.252** | -6.477 | **-9.564** |
| logKhsa^3^ (-1.5 / 1.5) | 1,16 | 1,489 | 0.888 | 1,171 | 1.437 | 1.405 | 1.103 | 1.355 | **1.717** | 0.635 | 0.631 | 0.923 | 1.370 | 1.253 | **2.035** |
| logBB^4^ ( -3.0 / 1.2) | 0,084 | -0,895 | -0.514 | -0,825 | 0.228 | -0.748 | -0.691 | 0.171 | -0.321 | -0.997 | -0.790 | -1.218 | -0.732 | -0.376 | -0.694 |
| Predicted CNS Activity^5^  (-2 to +2) | 1 | -1 | 0 | -1 | 1 | -1 | 0 | 1 | 0 | -1 | -1 | -2 | 0 | 1 | 0 |
| HERG K^+^ Channel Blockage: log IC50^6^  (concern < -5) | -3,94 | -5,265 | -4.029 | -4,662 | -4.108 | -4.902 | -4.288 | -4.001 | -5.902 | -5.447 | -5.258 | -4.791 | -6.448 | -5.935 | -5.418 |
| Apparent Caco-2 Permeability(nm/sec)^7^  (<25 poor, >500 great) | 3404 | 910 | 1109 | 706 | 3405 | 975 | 776 | 3405 | 3404 | 110 | 164 | 371 | 164 | 288 | 1455 |
| Apparent MDCK Permeability(nm/sec)^8^ (<25 poor, >500 great) | 1859 | 446M | 553 | 339 | 4414 | 690M | 376 | 3158 | 1859M | 50M | 77M | 169M | 78M | 142M | 742M |
| % Human Oral Absorption in GI (+/-20%)^9^ <25% is poor | 100 | 100 | 100 | 100 | 100 | 100 | 100 | 100 | 100 | 84 | 88 | 100 | 84 | 87 | 100 |
| Lipinski Ro5 Violations (max=4) | 1 | 1 | 0 | 0 | 1 | 1 | 0 | 1 | 1 | 0 | 0 | 0 | 1 | 1 | 2 |
| Jorgensen Ro3 Violations (max=3) | 1 | 1 | 1 | 1 | 2 | 2 | 2 | 2 | 2 | 1 | 1 | 2 | 2 | 2 | 2 |
|  | **ENT-A056** | **ENT-A065** | **ENT-A055** | **ENT-A066** | **ENT-A068** | **ENT-A069** | **ENT-A070** | **ENT-A075** | **ENT-A075 (protonated tertiary amine at pH=7.0±0.5)** | **ENT-A076** | **ENT-A077** | **ENT-A080** | **ENT-A080 (deprotonated carboxylic acid at pH=7.0±0.5)** | **ENT-A087** | **ENT-A088** |
| MW | 441.65 | 408.62 | 513.72 | 447.45 | 382.59 | 372.59 | 370.57 | 427.67 | 427.67 | 328.49 | 351.53 | 370.53 | 370.53 | 384.56 | 372.55 |
| Donor HB (0.0 / 6.0) | 1.000 | 1.000 | 1.000 | 1.000 | 1.000 | 1.000 | 1.000 | 1.000 | 1.000 | 1.000 | 1.000 | 2.000 | 2.000 | 1.000 | 1.000 |
| Accpt HB (2.0 / 20.0) | 6.150 | 2.450 | 4.950 | 2.450 | 2.450 | 2.450 | 2.450 | 4.450 | 4.450 | 3.700 | 3.200 | 3.700 | 3.700 | 3.700 | 4.700 |
| No. of Primary Metabolites (1.0 / 8.0) | **9** | 8 | 8 | 6 | 7 | 4 | 7 | 8 | 8 | 3 | 5 | 5 | 5 | 5 | 4 |
| No. of reactive groups | 0 | 0 | 0 | 0 | 0 | 0 | 0 | 0 | 0 | 1^c^ | 0 | 1^d^ | 1^d^ | 1^d^ | 1^b^ |
| logPo/w ^1^ (-2.0 / 6.5) | 4.642 | **6.510** | **7.118** | 6.204 | 5.975 | 5.683 | 5.736 | 5.603 | 5.593 | 3.778 | 4.522 | 4.503 | 4.528 | 5.023 | 3.960 |
| logS^2^ (-6.5 / 0.5) | -5.275 | **-8.019** | **-9.207** | **-7.439** | **-7.166** | **-6.704** | **-6.899** | -6.106 | -5.962 | -5.086 | **-6.999** | -5.779 | -5.748 | **-6.958** | -5.474 |
| logKhsa^3^ (-1.5 / 1.5) | 1.070 | **1.760** | **1.908** | **1.510** | **1.528** | 1.426 | 1.434 | 1.434 | 1.431 | 0.798 | 1.097 | 0.811 | 0.818 | 1.262 | 0.867 |
| logBB^4^ ( -3.0 / 1.2) | 0.307 | -0.029 | -0.864 | 0.153 | -0.006 | 0.010 | -0.003 | 0.183 | 0.192 | -0.379 | -0.585 | -0.883 | -0.858 | -0.532 | -0.431 |
| Predicted CNS Activity  (-2 to +2) | 1 | 0 | -1 | 1 | 0 | 1 | 0 | 1 | 1 | 0 | 0 | -1 | -1 | 0 | 0 |
| HERG K^+^ Channel Blockage: log IC50 (concern < -5) | -5.063 | -4.634 | -6.227 | -4.405 | -4.287 | -3.806 | -4.139 | -5.323 | -5.194 | -3.351 | -3.915 | -2.020 | -1.960 | -4.431 | -3.539 |
| Apparent Caco-2 Permeability (nm/sec) (<25 poor, >500 great) | 817 | 3405 | 1015 | 3406 | 3404 | 3404 | 3405 | 849 | 848 | 975 | 777 | 94 | 97 | 1081 | 905 |
| Apparent MDCK Permeability (nm/sec) (<25 poor, >500 great) | 440M | 1860 | 503M | 4253M | 1859 | 1859 | 1860 | 458 | 458 | 481 | 377 | 48 | 50 | 538 | 444 |
| % Human Oral Absorption in GI (+20%)  <25% is poor | 100 | 100 | 97 | 100 | 100 | 100 | 100 | 100 | 100 | 100 | 100 | 89 | 89 | 100 | 100 |
| Lipinski Ro5 Violations (max=4) | 0 | 1 | 2 | 1 | 1 | 1 | 1 | 1 | 1 | 0 | 0 | 0 | 0 | 1 | 0 |
| Jorgensen Ro3 Violations (max=3) | 1 | 2 | 2 | 1 | 2 | 1 | 2 | 2 | 2 | 0 | 1 | 1 | 1 | 1 | 0 |

^1^ logPo/w: Partitioning coefficient octanol / water; ^2^ logS: aqueous solubility; ^3^ logKhsa: binding to human serum albumin; ^4^logBB: brain/blood partition coefficient; ^5^Predicted CNS Activity: applied also as a predictor for the BBB cross; ^6^HERG K^+^ Channel Blockage: prediction of potential cardiotoxicity; ^7^Apparent Caco-2 Permeability(nm/sec): prediction for the permeability through the gut-blood barrier; ^8^Apparent MDCK Permeability(nm/sec): prediction for Madin-Darby canine kidney (MDCK) monolayers, good mimic for the BBB (for non-active transport); ^9^ Human Oral Absorption in GI: systemic drug absorption from the gastrointestinal (GI) tract.

Reactive FG (The presence of these groups can lead to false positives in HTS assays and to decomposition, reactivity, or toxicity problems *in vivo*): ^a^acceptor carbonyl or derivative; ^b^unhindered ester; ^c^carbonyl in 3-ring; ^d^acceptor carbonyl or derivative;

# Materials and Methods

## Chemistry

**General information**

Commercial reagents and solvents were purchased from Acros Organics, Fluka, Fluorochem, Merck or Sigma-Aldrich and used without further purification. Reactions were run in flame-dried glassware under an atmosphere of argon or nitrogen. All solvents were of analytical grade quality and were dried and/or purified according to standard procedures prior to use. Melting points were obtained with an Electrothermal Digital Melting Point Apparatus, Cole-Palmer ET0001 / Version 1.0, and are uncorrected. Optical rotations were measured with a P3000 series, Krüss Optronic polarimeter. The specific rotation ${[a]}_{D}^{24}$ was calculated according to the formula$\left[ a \right]_{D}^{24}=\frac{a}{c\cdot l}$ . The concentration of the sample is expressed in g/mL. NMR spectra were recorded on Varian (Varian, Palo Alto, CA, USA) Bruker spectrometers (Bruker BioSpin GmbH, Rheinstetten, Germany). ^1^H NMR spectra were recorded at 300 MHz or 400 MHz or 600 MHz, ^13^C NMR spectra were recorded at 75 MHz or 150 MHz and were internally referenced to residual solvent peaks. Chemical shifts are reported in *δ* units, parts per million (ppm) and coupling constants (*J*) are given in Hz. Low resolution mass spectra were recorded on a LC-MSn Fleet, Thermo Scientific mass spectrometer using methanol as solvent. High resolution mass spectra (HRMS) HRMS spectra were recorded in the APCI mode, on UPLC-MSn Orbitrap Velos-Thermo Scientific mass spectrometer. Flash column chromatography (FCC) was performed on Merck silica gel 60 (230–400 mesh) and thin layer chromatography (TLC) on precoated glass plates Merck 60 F254 (0.2 mm). Spots were visualized with UV light at 254 nm and phosphomolybdic acid stain (PMA, 10% in absolute ethanol). The purity of the final compounds was determined by high pressure liquid chromatography (HPLC) (Cecil Instruments Ltd., Cambridge, GBC Scientific Equipment, Brae-side, VIC, Australia Refractive Index detector. The analytical columns and methods are specified for each compound in the corresponding experimental section.

**(3*S*)-(t-butyldiphenylsilyloxy)-5-androsten-17-one (1a).** To a solution of DHEA (1.0 g, 3.47 mmol) in anhydrous tetrahydrofuran (10.4 mL) cooled to 0°C, iodine (2.6 g, 10.4 mmol) and imidazole (0.706 g, 10.4 mmol) were subsequently added, and the resulting mixture was stirred at 0°C for 30 min. Then tert-butylchlorodiphenylsilane (1.49 mL, 5.73 mmol) was added and the mixture was stirred at 25°C for 12 h. The reaction mixture was evaporated under reduced pressure and the residue was solubilized in ethyl acetate and the organic layer was washed with saturated sodium thiosulfate aqueous solution and brine and dried over Na_2_SO_4_ and the solvent was removed *in vacuo*. The residue was purified by FCC (elution solvent: cyclohexane/ethyl acetate: 9/1) and recrystallized from ethanol to obtain compound **1** (1.7 g, 93% yield) as a white powder. **Rf:** 0.39 (cyclohexane/ethyl acetate: 9/1); **mp**: 117 – 120°C; **^1^H NMR (600 MHz, CDCl_3_):** *δ* 7.73 – 7.66 (m, 4H), 7.44 – 7.34 (m, 6H), 5.15 (d, *J* = 5.1 Hz, 1H), 3.58-3.47 (m, 1H, 2.48 – 1.16 (m, 19H), 1.06 (s, 9H), 1.01 (s, 3H), 0.86 (s, 3H); **^13^C NMR (75 MHz, CDCl_3_):** *δ* 218.9, 135.8, 133.4, 129.9, 128.1, 121.2, 71.2, 58.6, 44.0, 42.2, 39.5, 38.8, 36.8, 34.2, 32.2, 31.6, 29.7, 27.3, 20.5, 20.3, 20.1, 19.3, 15.9, 14.3.

**(3*S*,17*R*)-17-allyl-3-(t-butyldiphenylsilyloxy)-5-androsten-17-ol (2a).** To a solution of **1a** (0.5 g, 0.95 mmol) in dry tetrahydrofuran (3.1 mL) cooled to 0°C allylmagnesium bromide (1.7 M in diethyl ether, 5.5 mL, 9.5 mmol) was added dropwise and the resulting solution was stirred overnight at 25°C for 12 h. The reaction was cooled to 0°C and quenched with saturated aqueous ammonium chloride solution and extracted with ethyl acetate. The organic layer washed with water and brine and dried over anhydrous Na_2_SO_4_, filtered and the filtrate concentrated under reduced pressure. The residue was purified via FCC (elution solvent: hexane/acetone: 95/5) to afford **2** as a white crystalline powder (0.51 g, 93%). **Rf:** 0.31 (cyclohexane/ethyl acetate: 9/1); **^1^H NMR (600 MHz, CDCl_3_):** *δ* 7.72 – 7.67 (m, 4H), 7.42 – 7.34 (m, 6H), 6.00 – 5.93 (m, 1H), 5.18 – 5.12 (m, 3H), 3.55 – 3.50 (m, 1H), 2.35 – 2.25 (m, 2H), 2.17 – 2.10 (m, 2H), 1.95-1.89 (m, 2H), 1.72-1.40 (m, 10H), 1.31-1.10 (m, 3H), 1.06 (s, 9H), 1.01 (s, 3H), 0.87 (s, 3H), 0.85-0.78 (m, 2H). **^13^C NMR (151 MHz, CDCl_3_):** *δ* 141.3, 135.68, 135.67, 135.3, 134.8, 134.75, 134.7, 134.69, 129.4, 129.37, 129.35, 127.6, 127.5, 127.39, 127.37, 120.8, 118.9, 82.4, 73.1, 50.9, 49.9, 45.9, 42.4, 41.7, 37.2, 36.5, 34.8, 32.7, 31.8, 31.64, 31.6, 26.9, 26.5, 23.8, 20.9, 20.6, 19.4, 19.1, 18.9, 14.2.

**(3*S*,17*R*)-17-allyl-17-(allyloxy)-3-(t-butyldiphenylsilyloxy)-5-androstene (3).** To a suspension of NaH (60% dispersion in mineral oil, 50 mg, 1,25 mmol; washed with anhydrous pentane) in anhydrous tetrahydrofuran (1.5 mL) was added a solution of **2a** (145 mg, 0.25 mmol) in anhydrous tetrahydrofuran (2.0 mL) and the reaction mixture was stirred at 80°C for 30 min. Subsequently, the reaction mixture was cooled to 25°C and a solution of allyl bromide (0.1 mL, 1.15 mmol) was added and the reaction mixture was stirred at 80°C for 12 h. The reaction was cooled to 25°C and was added to a mixture of ice and saturated aqueous ammonium chloride solution and extracted with diethyl ether. The organic layer was washed with brine, dried over Na_2_SO_4_ and the solvent was removed *in vacuo*. The crude product was pure enough to be used without further purification in the next step (150 mg, 96%). **Rf:** 0.83 (Petroleum ether 40-60°C/diethyl ether 95:5); **^1^H NMR (600 MHz, CDCl_3_):** *δ* 7.74 – 7.69 (m, 4H), 7.44 – 7.36 (m, 6H), 5.94 – 5.86 (m, 2H), 5.27 (dd, *J* = 1.7 and 17.2 Hz, 1H), 5.14 – 5.10 (m, 4H), 3.95 (d *J* = 5.3 Hz, 2H), 3.58 – 3.53 (m, 1H), 2.75 (dd, *J* = 4.8 and 15.2 Hz, 1H), 2.36 (t, *J* = 12.1 Hz, 1H), 2.16 (ddd, *J* = 1.97, 4.7, 13.3, 1H), 2.10 (dd, *J* = 7.7, 15.1, 1H), 1.94-1.87 (m, 2H), 1.73-1.40 (m, 10H), 1.32-1.14 (m, 3H), 1.09 (s, 9H), 1.02 (s, 3H), 0.94 (s, 3H), 0.91-0.79 (m, 2H); **^13^C NMR (151 MHz, CDCl_3_):** *δ* 141.1, 135.7, 135.4, 135.3, 134.9, 134.74, 134.71, 134.7, 129.5, 129.4, 129.3, 127.7, 127.6, 127.4, 127.39, 120.8, 117.2, 115.6, 87.4, 73.1, 64.2, 52.2, 49.8, 46.5, 42.4, 37.2, 36.5, 36.2, 34.3, 33.9, 32.4, 31.8, 31.6, 26.9, 26.8, 26.5, 23.5, 20.9, 19.3, 19.1, 13.9.

**(3*S*,17*R*)-3-(t-butyldiphenylsilyloxy)-3’,6’-dihydrospiro[5-androstene-17,2’-pyran] (4)** To a degassed solution of compound **3** (150 mg, 0.24 mmol) in anhydrous benzene (5 mL) was added Grubbs’ 2^nd^ generation catalyst (10 mg, 0.012 mmol) and the reaction was refluxed for 12 h. Subsequently the reaction was cooled to 25°C and the solvent is evaporated *in vacuo*. The residue was purified by FCC (elution solvent: hexane/diethyl ether: 95/5) to afford compound **4** as a white solid (114 mg, 79% yield). **Rf:** 0.57 (petroleum ether 40-60°C/diethyl ether: 95/5); **mp:** 142-144°C; **^1^H NMR (600 MHz, CDCl_3_):** *δ* 7.68 – 7.66 (m, 4H), 7.42 – 7.34 (m, 6H), 5.75 – 5.73 (m, 1H), 5.68-5.67 (m, 1H), 5.11 (d, *J* = 5.2 Hz, 1H), 4.22-4.14 (m, 2H), 3.55 – 3.49 (m, 1H), 2.35-2.31 (m, 1H), 2.15-2.12 (m, 1Η), 1.94-1.81 (m, 3H), 1.72-1.39 (m, 10H), 1.32-1.11 (m, 3H), 1.06 (s, 9H), 1.00 (s, 3H), 0.87 (s, 3H), 0.84-0.77 (m, 2H); **^13^C NMR (151 MHz, CDCl_3_):** *δ* 141.3, 135.76, 135.75, 134.8, 134.7, 129.42, 129.4, 127.44, 127.4, 125.8, 123.4, 120.8, 83.1, 73.2, 63.3, 51.1, 50.0, 45.8, 42.4, 37.2, 36.5, 33.2, 33.0, 32.5, 31.8, 31.7, 31.2, 27.0, 23.3, 20.8, 19.4, 19.1, 13.6; **HR-MS (APCI^+^):** calcd for C_39_H_53_O_2_Si [M+H]^+^ 581.3809, found 581.3797, calcd for C_39_H_51_OSi [M-Η_2_Ο+Η]^+^ 563.3704, found 563.3694.

(**3*S*)-(t-butyldimethylsilyloxy)-5-androsten-17-one (1b).** To a solution of DHEA (2 g, 6.93 mmol) in anhydrous tetrahydrofuran (20 mL) at 0°C were sequentially added iodine (5.28 g, 20.80 mmol) and imidazole (1.4 g, 20.56 mmol) and the resulting mixture was stirred for 1h at the same temperature. Subsequently tert-butylchlorodimethylsilane (1.1 g, 7.43 mmol) was added and the reaction was stirred at 25°C overnight and the solvent was evaporated under reduced pressure. The residue was solubilized in ethyl acetate and the organic layer was washed with sodium thiosulfate and brine and dried over Na_2_SO_4_ and the solvent was removed *in vacuo*. The residue was was purified by FCC (elution solvent: ethyl acetate/hexane: 2/98) to obtain compound **1b** (2.76 g, yield quantitative) as a white powder. **R_F_:** 0.33 (petroleum ether 40-60° C/ethyl acetate: 90/10); **mp:** 147 – 149°C. **^1^H NMR (600 MHz, CDCl_3_):** *δ* 5.37 – 5.31 (m, 1H), 3.54 – 3.44 (m, 1H), 2.46 (dd, *J* = 19.3, 8.8 Hz, 1H), 2.31 – 2.17 (m, 2H), 2.13 – 2.04 (m, 2H), 1.98 – 1.91 (m, 1H), 1.88 – 1.79 (m, 2H), 1.76 – 1.60 (m, 4H), 1.58 – 1.42 (m, 2H), 1.32 – 1.24 (m, 2H), 1.10 – 0.96 (m, 2H), 1.03 (s, 3H), 0.89 (s, 9H), 0.88 (s, 3H), 0.06 (s, 6H). **^13^C NMR (75 MHz, CDCl_3_):** *δ* 221.3, 141.9, 120.5, 72.6, 51.9, 50.5, 47.7, 42.9, 37.4, 36.9, 36.0, 32.1, 31.7, 31.6, 31.0, 26.1, 22.0, 20.5, 19.6, 18.4, 13.7, -4.4; **HR-MS (APCI^+^):** m/z calcd for C_25_H_42_O_2_Si 425.2847 [M+Na]^+^, found 425.2846.

**(3*S*,17*R*)-17-allyl-3-(t-butyldimethylsilyloxy)-5-androsten-17-ol (2b).** To a solution of **1b** (5.00 g, 12.42 mmol) in dry tetrahydrofuran (12.4 mL) at 0°C was added allyl-magnesium bromide (1M in diethyl ether, 8.6 mL, 18.63 mmol) portion wise over 4 min and the resulting solution was stirred overnight at 25°C. The reaction was quenched with saturated aqueous ammonium chloride solution and the solvent was evaporated under reduced pressure. To the residue was added ethyl acetate and the organic layer washed with brine and dried dried over Na_2_SO_4_ and the solvent was removed *in vacuo*. The residue was purified via FCC (elution solvent: petroleum ether 35-60°C/ethyl acetate: 97/3) to obtain **2b** as white solid (5.32 g, 97%). **mp:** 102 – 104°C. **R_F_:** 0.22 (PE 40-60°C / EA 95:5). **^1^H NMR (600 MHz, CDCl_3_):** *δ* 5.99 (ddt, *J* = 17.2, 10.1, 7.2 Hz, 1H), 5.32 – 5.30 (m, 1H), 5.22 – 5.11 (m, 2H), 3.54 – 3.40 (m, 1H), 2.33 – 2.23 (m, 2H), 2.23 – 2.14 (m, 2H), 2.04 – 1.90 (m, 2H), 1.85 – 1.78 (m, 1H), 1.76 – 1.68 (m, 1H), 1.63 – 1.44 (m, 6H), 1.36 – 1.15 (m, 4H), 1.08 – 1.03 (m, 1H), 0.95 – 0.85 (m, 3H), 1.02 (s, 3H), 0.90 (s, 3H), 0.89 (s, 9H), 0.06 (s, 6H); **^13^C NMR (151 MHz, CDCl_3_):** *δ* 143.0, 141.7, 135.1, 121.0, 117.4, 116.0, 87.8, 72.7, 63.4, 52.3, 50.1, 46.9, 45.8, 42.9, 37.6, 36.8, 36.7, 34.4, 34.1, 32.8, 32.2, 31.86, 26.1, 21.1, 19.6, 18.4, 14.1, -4.4; **HR-MS (APCI^+^):** m/z calcd for C_28_H_48_O_2_SiNa 467.3316 [M+Na]^+^, found 467.3315.

(**3*S*,17*R*)-3-(t-butyldimethylsilyloxy)-17-allyl-17-((2-(chloromethyl)allyl)oxy)-5-androstene (5).** To a flamed dried round-bottom flask at 0°C were added dry tetrahydrofuran (15.6 mL), **2b** (1.35 g, 3.12 mmol), NaH (60% in oil, 1.25 g, 31.2 mmol), 3-chloro-2-chloromethyl-propene (1.65 mL, 15.6 mmol) and dry tetrahydrofuran (15.6 mL). The mixture was refluxed for 12h and the reaction was cooled to 0°C and was quenched with saturated aqueous NH_4_Cl solution and THF was evaporated under reduced pressure. The residue was dissolved in ethyl acetate and the organic layer washed with brine, dried over Na_2_SO_4_ and concentrated *in vacuo*. The residue was purified by FCC (elution solvent: petroleum ether 35-60°C/ethyl acetate: 98/2) to obtain **2b** as white solid (5.32 g, 97%). **Rf:** 0.40 (petroleum ether 35-60°C/ethyl acetate: 99/1);$\left[ a \right]_{D}^{24}=- 16.45^{\circ}$(*c* = 1.26 g/mL, CHCl_3_); **^1^H NMR (600 MHz, CDCl_3_):** *δ* 5.94 – 5.85 (m, 1H), 5.33 – 5.28 (m, 1H), 5.22 (d, *J* = 8.2 Hz, 2H), 5.16 – 5.09 (m, 2H), 4.11 (s, 2H), 4.03 (ABq, *J_AB_* = 6.6 Hz, 2H), 3.52 – 3.43 (m, 1H), 2.80 – 2.74 (m, 1H), 2.31 – 2.22 (m, 1H), 2.22 – 2.13 (m, 2H), 2.02 – 1.68 (m, 6H), 1.62 – 1.46 (m, 7H), 1.34 – 1.17 (m, 3H), 1.07 – 1.03 (m, 1H), 1.01 (s, 3H), 0.94 (s, 3H), 0.89 (s, 9H), 0.06 (s, 6H). **^13^C NMR (151 MHz, CDCl_3_):** *δ* 143.0, 141.7, 135.1, 121.0, 117.4, 116.1, 87.8, 72.7, 63.4, 52.6, 50.2, 46.9, 45.8, 42.9, 37.6, 36. 8, 36.7, 34.4, 34.1, 32.8, 32.2, 31.9, 26.1, 21.1, 19.6, 18.4, 14.1, -4.4; **HR-MS (APCI^+^):** m/z calcd for C_32_H_54_^35^ClO_2_Si: 532.3576 [M+H]^+^, found 533.3573.

**(3*S*,17*R*)-3-(t-butyldimethylsilyloxy)-5'-(chloromethyl)-3’,6’-dihydrospiro[5-androstene-17,2’-pyran] (6)** To a degassed solution of **5** (2.97 g, 5.57 mmol) in dichloromethane (55.7 mL) was added Hoveyda-Grubbs 2^nd^ Generation Catalyst (106.5 mg, 0.17 mmol). The solution was stirred for 20’ at 25°C and for 4.5h at reflux temperature. The solvent was evaporated under reduced pressure and the residue was purified by FCC (elution solvent: petroleum ether 35-60°C/diethyl ether: 96/4) to afford **6**, as a white solid in quantitative yield (2.81 g). **mp:** 136 – 138°C; **R_F_:** 0.46 (petroleum ether 35-60°C/diethyl ether: 96/4); $\left[ a \right]_{D}^{24}=-16.48^{\circ}$(*c* = 1.82 g/mL, CHCl_3_); **^1^H NMR (300 MHz, CDCl_3_):** *δ* 5.88 (s, 1H), 5.33 – 5.28 (m, 1H), 4.29 and 4.21 (two d, ABq, *J_AB_* = 16.6 Hz, 2H), 3.99 (s, 2H), 3.51 – 3.43 (m, 1H), 1.01 (s, 3H), 2.43 – 0.82 (m, 11H), 0.91 (s, 3H), 0.89 (s, 9H), 0.05 (s, 6H). **^13^C NMR (151 MHz, CDCl_3_):** *δ* 141.8, 133.0, 124.4, 120.9, 83.2, 72.7, 63.8, 51.6, 50.3, 45.9, 45.8, 43.0, 37.6, 36.8, 33.8, 33.3, 32.7, 32.2, 31. 9, 31.53, 26.1, 23.5, 21.0, 19.6, 18.4, 13.7, -4.4; **HR-MS (APCI^+^):** m/z calcd for C_30_H_50_^35^ClO_2_Si 504.3159 [M+H]^+^, found m/z 505.3258.

**(3*S*,17*R*)-3-(t-butyldimethylsilyloxy)-5’-(p-methoxybenzylmethyloxy)-3’,6’-dihydrospiro[5-androsten-17,2’-pyran] (7).** To a suspension of sodium hydride (60% in oil, 53.6 mg, 13.40 mmol) in dry tetrahydrofuran (1.0 mL) at 0°C was added compound **6** (68 mg, 0.13 mmol) and 4-methoxybenzylalcohol (89.8 mg, 0.65 mmol) and the reaction was refluxed for 12 h. The reaction was quenched with saturated aqueous ammonium chloride solution and the solvent was evaporated under reduced pressure. The residue was dissolved in ethyl acetate and the organic layer washed with brine, dried over Na_2_SO_4_ and concentrated *in vacuo*. The residue was purified by FCC (elution solvent: ethyl acetate/petroleum ether 35-60°C: 4/96) to afford **7**, as a white solid (79 mg, 97% yield). **Rf:** 0.47 (ethyl acetate/petroleum ether 35-60°C: 1/9); **mp:** 95 – 97°C; $\left[ a \right]_{D}^{24}=-108.4^{\circ}$(*c* = 0.08 g/mL, acetone); **^1^H NMR (600 MHz, CDCl_3_):** *δ* 7.24 (d, *J* = 8.6 Hz, 2H), 6.87 (d, *J* = 8.5 Hz, 2H), 5.75 (s, 1H), 5.40 – 5.24 (m, 1H), 4.38 (s, 2H), ), 4.23 and 4.18 (two d, ABq, *J_AB_* = 16.3 Hz, 2H), 3.89 (s, 2H), 3.80 (s, 3H), 3.58 – 3.45 (m, 1H), 2.42 – 0.86 (m, 21H), 1.02 (s, 3H), 0.91 (s, 3H), 0.89 (s, 9H), 0.06 (s, 6H). **^13^C NMR (75 MHz, CDCl_3_):** *δ* 159.3, 141.8, 133.8, 130.5, 129.4, 121.9, 120.9, 113.9, 83.3, 72.8, 71.3, 71.0, 64.2, 51.4, 50.3, 46.0, 42.9, 37.6, 36.8, 33.5, 33.2, 32.7, 32.2, 31.9, 31.2, 29.8, 26.1, 23.5, 22.8, 21.0, 19.6, 18.4, 14.3, 13.8, -4.4; **HR-MS (ESI+):** m/z calcd for C_38_H_59_O_4_Si [M+H]^+^ 607.4177, found 607.4189; m/z calcd for C_38_H_57_O_2_SiNa C_38_H_58_O_4_Si [M+Na]^+^ 629.3997, found 629.4006.

**(3*S*,17*R*)-3-(t-butyldimethylsilyloxy)-3’,6’-dihydrospiro[5-androstene-17,2’-pyran]-5’-yl-methanol (8).** To a solution of compound **7** (190 mg, 0.21 mmol) in dichloromethane (60 mL) and phosphate buffered saline pH=7.0 (1.8 mL) was added 2,3-dichloro-5,6-dicyano-1,4-benzoquinone (DDQ) (77.0 mg, 0.34 mmol). The reaction mixture was stirred at 25°C for 2 h and was quenched with saturated aqueous sodium bicarbonate and extracted with dichloromethane. The organic layer was washed with brine, dried over Na_2_SO_4_, and concentrated *in vacuo*. The residue was purified by FCC (elution solvent: ethyl acetate/petroleum ether 35-60°C: 2/8) to obtain **9**, as a white solid (106 mg, 70% yield). **Rf:** 0.17% (ethyl acetate/petroleum ether 35-60°C: 17/83); **mp**: 176 – 178°C; $\left[ a \right]_{D}^{24}=-80.0^{\circ}$(*c* = 0.10 g/mL, acetone); **^1^H NMR (300 MHz, CDCl_3_):** *δ* 5.75 (s, 1H), 5.35 – 5.27 (m, 1H), 4.26 and 4.18 (two d, ABq, *J_AB_* = 16.4 Hz, 2H), 4.03 (s, 2H), 3.47 (s, 1H), 2.44 – 0.84 (m, 8H), 1.01 (s, 3H), 0.91 (s, 3H), 0.89 (s, 9H), 0.06 (s, 6H); **^13^C NMR (75 MHz, CDCl_3_):** *δ* 141.8, 136.4, 120.9, 120.4, 83.4, 72.7, 64.3, 63.8, 51.5, 50.3, 45.9, 42.9, 37.6, 36.8, 33.6, 33.3, 32.7, 32.2, 31.9, 31.2, 29.9, 26.1, 23.5, 21.0, 19.6, 18.4, 13.7, -4.4; HR-MS (ESI^+^): m/z calcd for C_30_H_51_O_3_Si [M+H]^+^ 487.3602, found m/z 487.3600; m/z calcd for C_30_H_50_O_3_SiNa [M+Na]^+^ 509.3422, found m/z 509.3421.

**(3*S*,17*R*)-5'-(azidomethyl)-3’,6’-dihydrospiro[5-androstene-17,2’-pyran]-3-ol (9).** To a solution of **ENT-A025** (96.0 mg, 0.25 mmol) in a mixture of water/acetone (1:4, 11.8 mL) was added sodium azide (32.5 mg, 0.50 mmol) and potassium iodide (20.8 mg, 0.13 mmol) and the resulting mixture was stirred at 25°C for 12h. The reaction mixture was concentrated *in vacuo* and the residue partitioned between dichloromethane and water. The organic layers were washed with brine, dried over anhydrous Na_2_SO_4_, and concentrated under reduced pressure to afford compound **9** which was used in the next step without further purification (99 mg, quantitative). **Rf:** 0.30 (ethyl acetate/petroleum ether 35 – 60°C: 40/60); **^1^H NMR (300 MHz, CDCl_3_):** *δ* 5.81 (bs, 1H), 5.40 – 5.31 (m, 1H), 4.28 and 4.20 (two d, ABq, *J_AB_* = 16.7 Hz, 2H), 3.68 and 3.63 (two d, ABq, *J_AB_* = 14.4 Hz, 2H),3.58 – 3.44 (m, 1H), 2.47 – 0.84 (m, 21H), 1.02 (s, 3H), 0.92 (s, 3H).

**(3*S*,17*R*)-3-(t-butyldimethylsilyloxy)-3’,6’-dihydrospiro[5-androstene-17,2’-pyran]-5’-carbaldehyde (10).** To a solution of compound **6** (1.09 g, 2.16 mmol) in anhydrous acetone/dicholoromethane (3/1, 21.6 mL) was added NaI (0.81 g, 5.4 mmol) and the resulting mixture was heated to 75°C for 3h. After cooling down the reaction mixture to room temperature, *N*-methyl morpholine-*N*-oxide (1.01 g, 8.64 mmol) was added, and the mixture was stirred at 25°C for 14h. The reaction was quenched with a sodium thiosulphate saturated aqueous solution and extracted with dichloromethane. The organic layer was washed with brine, dried over anhydrous Na_2_SO_4_, and concentrated under reduced pressure. The residue was purified by FCC (elution solvent: petroleum ether 35-60°C/ethyl acetate: 94/6) to aldehyde **10**, as a white solid (0.754 g, 72% yield). **Rf:** 0.63 (petroleum ether 35-60°C/ethyl acetate: 85/15); **mp:** 155 - 157°C; $\left[ a \right]_{D}^{24}=-32.7^{\circ}$(*c* = 0.28 g/mL, acetone); **^1^H NMR (600 MHz, CDCl_3_):** *δ* 9.40 (s, 1H), 6.89 (bs, 1H), 5.32 – 5.28 (m, 1H), 4.45 and 4.35 (two d, ABq, *J_AB_* = 17.2 Hz, 2H), 3.51 – 3.42 (m, 1H), 2.66 – 2.62 (m, 1H), 2.28 – 0.86 (m, 20H), 1.01 (s, 3H), 0.92 (s, 3H), 0.88 (s, 9H), 0.05 (s, 6H); **^13^C NMR (75 MHz, CDCl_3_):** *δ* 191.7, 147.1, 141.9, 140.0, 120.8, 83.7, 72.7, 60.9, 51.7, 50.3, 46.1, 42.9, 37.6, 36.8, 34.1, 33.4, 32.9, 32.7, 32.2, 31.9, 29.9, 26.1, 23.5, 20.9, 19.6, 18.4, 14.3, 13.6, -4.4; **HR-MS (ESI):** m/z calcd for C_30_H_48_O_3_SiNa 507.3265 [M+Na]^+^, found m/z 507.3272.

**(3*S*)-17-methylene-5-androsten-3-ol (11).** To a solution of dry methyl-triphenyl-phosphonium bromide (14.86 g, 41.60 mmol) in anhydrous tetrahydrofuran (74.9 mL), at 0°C was added potassium tert-butoxide (4.67 g, 41.60 mmol) and the resulting mixture was stirred for 30min at 0°C. Subsequently, a solution of **DHEA** (3.00 g, 10.40 mmol) in anhydrous tetrahydrofuran (14.6 mL) was added and the mixture was stirred at 25°C for 16h. The reaction was quenched at 0°C by the slow addition of ice, which led to the formation of a precipitate that was filtered under vacuum using a sintered funnel. The solid was washed with 10% hydrochloric acid aqueous solution, water, and hexane, and was dissolved in dichloromethane. The organic layer was dried over anhydrous Na_2_SO_4_ and concentrated under reduced pressure and dried under high vacuum to afford compound **11** as a white solid (2.98 g, yield quantitative) which was used for the synthesis of compound **12** without any further purification. **Rf:** 0.20 (hexane/ethyl acetate: 7/3); **mp:** 202 – 204°C; $\left[ a \right]_{D}^{24}=-64.94^{\circ}$(*c* = 1.54 g/mL, CHCl_3_); **^1^H NMR (600 MHz, CDCl_3_):** *δ* 5.36 – 5.32 (m, 1H), 4.63 (s, 1H), 4.62 (s, 1H), 3.54 – 3.46 (m, 1H), 2.51 – 0.85 (m, 20H), 1.01 (s, 3H), 0.78 (s, 3H); **^13^C NMR (151 MHz, CDCl_3_):** *δ* 161.8, 141.0, 121.5, 101.0, 71.7, 54.8, 50.5, 44.0, 42.4, 37.4, 36.7, 35.6, 31.9, 31.8, 31.7, 29.5, 24.4, 21.1, 19.5, 18.4; **HR-MS (APCI^+^):** m/z calcd for C_20_H_31_O 287.2369 [M+H]^+^, found 287.2373.

**(3*S*)-17-methylene-5-androsten-3-yl acetate (12).** To a solution of compound **11** (2.98 g, 10.40 mmol) in dry dichloromethane (112.7 mL) were sequentially added DMAP (25.7 mg, 0.21 mmol), triethylamine (1.5 mL, 20.80 mmol) and acetic anhydride (1.1 ml, 11.44 mmol). The resulting colourless solution was stirred at 25°C for 5h. The reaction was quenched with the slow addition of water. Dichloromethane was added and the organic layer was washed with brine, dried over anhydrous Na_2_SO_4_, and concentrated under reduced pressure. The residue was purified by FCC (elution solvent: hexane/ethyl acetate: 85/15) to afford compound **12,** as a white solid (3.42 g, quantitative yield). **Rf:** 0.70 (petroleum ether 35-60°C/ethyl acetate: 7/3); **mp:** 101 – 103°C; $\left[ a \right]_{D}^{24}=-71.94^{\circ}$(*c* = 1.39 g/mL, CHCl_3_); **^1^H NMR (600 MHz, CDCl_3_):** *δ* 5.40 – 5.35 (m, 1H), 4.63 (s, 1H), 4.62 (s, 1H), 4.62 – 4.55 (m, 1H), 2.51 – 0.95 (m, 19H), 2.02 (s, 3H), 1.03 (s, 3H), 0.79 (s, 3H); **^13^C NMR (151 MHz, CDCl_3_):** *δ* 170.6, 161.7, 139.9, 122.5, 101.1, 74.0, 54.8, 50.4, 44.0, 38.2, 37.1, 36.8, 35.6, 31.9, 31.8, 29.5, 27.9, 24.4, 21.5, 21.0, 19.5, 18.4; **HR-MS (APCI^+^):** m/z calcd for C_22_H_33_O_2_ 329.2475 [M+H]^+^, found 329.2475.

**(3*S*)-3’-oxo-spiro[5-androsten-17-cyclobutan]-3-yl acetate (13).** To a solution of compound **12** (1.28 g, 3.91 mmol) in anhydrous tetrahydrofuran (7.8 mL) was added activated Zinc dust (1.28 g, 19.55 mmol). The resulting grey suspension was stirred at 25°C for 5min and was added dropwise over 30min to a solution of trichloro acetyl chloride (1.8 mL, 15.64 mmol) and phosphoryl chloride (1.46 mL, 15.64 mmol) in anhydrous tetrahydrofuran (7.8 mL). The resulting suspension was stirred at 30°C for 3 h. The reaction mixture was filtered through celite, and the cake was washed with tetrahydrofuran. The filtrate was then concentrated under reduced pressure and the residue was dissolved in a solution of hexane/diethyl ether (97.5/2.5; 80 mL). The organic layer was washed with saturated aqueous sodium bicarbonate solution and brine dried over anhydrous Na_2_SO_4_ and concentrated under reduced pressure. The residue was dissolved in acetic acid (46.9 mL) and added of activated Zinc dust (2.56 g, 39.10 mmol). The resulting suspension was equipped with a condenser and stirred at 120°C for 24 h. Upon completion of the reaction the mixture was filtered through celite, and the cake was washed with a mixture of hexane/diethyl ether (97.5/2.5). The filtrate was then neutralized with 1M potassium hydroxide, and the two layers were separated. The organic layer was washed with brine and dried over anhydrous Na_2_SO_4_ and concentrated under reduced pressure. The residue was purified by FCC (elution solvent: petroleum ether 35-60°C/diethyl ether: 9/1) to obtain compound **13**, a white solid (725.0 mg, 50% yield over two steps). **Rf:** 0.42 (petroleum ether 35-60°C/ethyl acetate: 8/2); **mp:** 105 – 107°C; $\left[ a \right]_{D}^{24}=-110.47^{\circ}$(*c* = 1.72 g/mL, CHCl_3_); **^1^H NMR (400 MHz, CDCl_3_):** *δ* 5.40 – 5.33 (m, 1H), 4.68 – 4.52 (m, 1H), 3.11 (ddd, *J* = 17.2, 4.1, 2.6 Hz, 1H), 3.01 (ddd, *J* = 17.1, 4.0, 2.4 Hz, 1H), 2.65 (ddd, *J* = 17.2, 5.9, 2.5 Hz, 1H), 2.45 (ddd, *J* = 17.1, 6.0, 2.5 Hz, 1H), 2.35 – 0.77 (m, 19 H), 2.02 (s, 3H), 1.04 (s, 3H), 0.78 (s, 3H). **^13^C NMR (151 MHz, CDCl_3_):** *δ* 208.1, 170.6, 139.8, 122.4, 73.9, 54.3, 52.6, 51.4, 50.0, 43.4, 42.8, 38.2, 37.5, 37.2, 36.8, 33.0, 32.0, 31.9, 27.8, 24.8, 21.5, 20.6, 19.5, 14.6; **HR-MS (APCI^+^):** m/z calcd for C_24_H_35_O_3_ 371.2581 [M+H]^+^, found 371.2578.

**(3*S*,17*R*)- 5’-(hydroxymethyl)-3’,6’-dihydrospiro[5-androstene-17,2’-pyran]-3-ol (ENT-A008).** To a solution of compound **8** (14.0 mg, 0.03 mmol) in anhydrous dichloromethane (0.9 mL) at 0°C was added HF·pyridine complex (5.0 μL, 0.3 mmol) and the reaction mixture was stirred at 25°C for 6 h. The reaction was quenched with water and extracted with dichloromethane. The organic layer was washed with brine, dried over Na_2_SO_4_, and concentrated *in vacuo*. The residue was purified by FCC (elution solvent: ethyl acetate/petroleum ether 35-60°C: 4/6) to afford **ENT-A008**, as a white solid (8 mg, 73% yield). **Rf:** 0.03 ( ethyl acetate/petroleum ether 35-60°C: 25/75); **mp:** 195 – 197°C; $\left[ a \right]_{D}^{24}=-80.0^{\circ}$(*c* = 0.08 g/mL, acetone);**^1^H NMR (600 MHz, CDCl_3_):** *δ* 5.75 (s, 1H), 5.37 – 5.32 (m, 1H), 4.26 and 4.19 (two d, ABq, *J_AB_* = 14.7 Hz, 2H), 4.02 (s, 2H), 3.52 (s, 1H), 2.42 – 0.86 (m, 23H), 1.02 (s, 3H), 0.92 (s, 3H); **^13^C NMR (75 MHz, CDCl_3_):** *δ* 141.0, 136.4, 121.5, 120.4, 83.3, 71.9, 64.2, 63.8, 51.4, 50.2, 45.9, 42.4, 37.4, 36.7, 33.6, 33.9, 32.7, 31.9, 31.2, 29.8, 23.5, 21.0, 19.5, 13.7. **HR-MS (APCI^+^):** m/z calculated for C_24_H_37_O_3_ m/z 373.2737 [M+H]^+^, found m/z 373.2734; **HPLC:** 100% purity, RT = 37.64 min. Column: Kromasil 100-10-SIL (10 x 250 mm, 10 μm), Method: eluting with 35% ethyl acetate – 65% toluene, isocratic, flow rate 2.1 mL/min at 25°C, injection volume 20 μL.

**Methyl (3*S*,17*R*)-3-hydroxy-3’,6’-dihydrospiro[5-androstene-17,2’-pyran]-5’-yl-methylthio acetate (ENT-A026).**

**Step 1.** To a solution of **ENT-A025** (15.0 mg, 0.04 mmol), in anhydrous acetone (0.3 mL) was added NaI (12 mg, 0.08 mmol) and the resulting solution was stirred overnight at 25°C. The solvent was evaporated under reduced pressure and the residue partitioned between ethyl acetate and water. The organic layer was washed with brine, dried over Na_2_SO_4_, and concentrated under reduced pressure to afford the crude iodo congener of **ENT-A025**.

**Step 2.** A solution of methyl thioglycolate (4.3 µL, 0.05 mmol) and DIPEA (21.0 µL, 0.12 mmol) in anhydrous dichloromethane (0.1 mL) was stirred at 25°C for 5 min and then was cooled to 0°C and a solution of the crude product from step 1 above in dichloromethane (0.3 mL) was added. The reaction was stirred for 12h and then diluted with dichloromethane and the organic phase washed with aq. K_2_CO_3_ 1M, brine, dried over Na_2_SO_4_, and concentrated under reduced pressure. The residue was purified by FCC (elution solvent: ethyl acetate/petroleum ether 35-60°C: 3/7) to afford **ENT-A026**, as a white solid (18.4 mg, quantitative yield). **Rf:** 0.24 (ethyl acetate/petroleum ether 35-60°C: 3/7); **mp:** 97 – 99°C; $\left[ a \right]_{D}^{24}=-44.78^{\circ}$(*c* = 0.00134 g/mL, CHCl_3_); **^1^H NMR (300 MHz, CDCl_3_):** *δ* 5.69 (bs, 1H), 5.38 – 5.30 (m, 1H), 4.24 and 4.16 (two d, ABq, *J_AB_* = 15.5 Hz, 2H), 3.73 (s, 3H), 3.52 – 3.50 (m, 1H), 3.16 (s, 4H), 2.44 – 0.82 (m, 22H), 1.01 (s, 3H), 0.90 (s, 3H); **^13^C NMR (75 MHz, CDCl_3_):** *δ* 171.1, 141.0, 131.1, 123.1, 121.5, 83.3, 71.9, 64.3, 51.4, 50.2, 45.9, 42.4, 37.4, 36.7, 34.9, 33.5, 33.2, 32.6, 32.0, 31.8, 31.7, 31.5, 29.8, 23.5, 21.0, 19.5, 13.8; **HR-MS (APCI^+^):** m/z calculated for C_27_H_41_O_4_S 461.2720 [M+H]^+^, found 461.2718. **HPLC:** 100% purity, RT = 34.86 min. Column: Kromasil 100-10-SIL (10 x 250 mm, 10 μm), Method: eluting with 30% ethyl acetate – 70% cyclohexane, isocratic, flow rate 1.5 mL/min at 25°C, injection volume 20 μL.

**(3*S*,17*R*)-5'-(fluoromethyl)-3’,6’-dihydrospiro[5-androstene-17,2’-pyran]-3-ol (ENT-A034).** To a solution of compound **6** (40.4 mg, 0.08 mmol) in anhydrous tetrahydrofuran (2.4 mL) at 0°C was added TBAF (237.8 μL, 0.24 mmol). The reaction mixture was stirred overnight at 25°C. The reaction was quenched with brine and extracted with ethyl acetate. The organic layer was washed with brine, dried over Na_2_SO_4,_ and concentrated *in vacuo*. The residue was purified by FCC (elution solvent: ethyl acetate/petroleum ether 35-60°C:4/6) to afford **ENT-A034**, as a white solid (32 mg, 100%). **Rf:** 0.30 (ethyl acetate/petroleum ether 35-60°C: 4/6); **mp:** 115 – 117°C; $\left[ a \right]_{D}^{24}=+15.87^{\circ}$(*c* = 0.00126 g/mL, CHCl_3_); **^1^H NMR (600 MHz, CDCl_3_):** *δ* 5.88 (bs, 1H), 5.36 – 5.32 (m, 1H), 4.73 (d, *J_HF_* = 48.0 Hz, 2H), 4.28 and 4.22 (2d, ABq, *J* = 16.6 Hz, 2H), 3.55 – 3.48 (m, 1H), 0.85 – 2.44 (m, 21H), 1.02 (s, 3H), 0.92 (s, 3H). **^13^C NMR (151 MHz, CDCl_3_):** *δ* 141.0, 132.8 (d, *J_CF_* = 15.9 Hz), 124.8 (d, *J_CF_* = 10.0 Hz), 121.5, 84.6, 83.3 (d, *J_CF_* = 45.9 Hz), 71.9, 63.4, 51.5, 50.2, 46.0, 42.4, 37.4, 36.7, 33.6, 33.3, 32.7, 31.9, 31.8, 31.2, 23.5, 21.0, 19.5, 13.7; **^19^F NMR (282 MHz, CDCl_3_):** *δ* -212.37 (t, *J* = 48.0 Hz). **HR-MS (APCI^+^):** m/z calculated for C_24_H_36_FO_2_ m/z 375.2694 [M+H]^+^, found m/z 375.2691; **HPLC:** 100% purity, RT = 34.90 min. Column: Kromasil 100-10-SIL (10 x 250 mm, 10 μm), Method: eluting with 25% ethyl acetate – 75% cyclohexane, isocratic, flow rate 1.5 mL/min at 25°C, injection volume 20 μL.

**(3*S*,17*R*)-5’-(p-methoxybenzylmethyloxy)-3’,6’-dihydrospiro[5-androsten-17,2’-pyran]-3-ol (ENT-A035).** To a solution of compound **7** (79 mg, 0.13 mmol) in anhydrous tetrahydrofuran (1.0 mL) at 0°C was added tetra-butyl ammonium fluoride solution (1M in tetrahydrofuran; 0.5 mL, 0.5 mmol) and the reaction mixture was stirred at 25°C for 6 h. The reaction was quenched with water and extracted with ethyl acetate. The organic layer was washed with brine, dried over Na_2_SO_4_, and concentrated *in vacuo*. The residue was purified by FCC (elution solvent: ethyl acetate/petroleum ether 35-60°C: 4/96) to afford **ENT-A035**, as a white solid (67 mg, quantitative yield). **Rf:** 0.3 (ethyl acetate/petroleum ether 35-60°C: 3/7); **mp:** 68 – 70°C; $\left[ a \right]_{D}^{24}=+46.48^{\circ}$(*c* = 0.00128 g/mL, CHCl_3_); **^1^H NMR (300 MHz, CDCl_3_):** *δ* 7.24 (d, *J* = 8.6 Hz, 2H), 6.87 (d, *J* = 8.6 Hz, 2H), 5.75 (bs, 1H), 5.36 - 5.32 (m, 1H), 4.38 (s, 2H), 4.24 and 4.18 (two d, ABq, *J_AB_* = 18.3 Hz, 2H), 3.89 (s, 2H), 3.80 (s, 3H), 3.57 - 3.45 (m, 1H), 1.02 (s, 3H), 0.91 (s, 3H). **^13^C NMR (75 MHz, CDCl_3_):** *δ* 159.3, 141.0, 133.8, 129.4, 122.0, 121.5, 113.9, 83.3, 71.9, 71.3, 71.0, 64.2, 55.4, 51.4, 50.2, 45.9, 42.4, 37.4, 36.7, 33.4, 33.2, 32.7, 31.8, 31.8, 31.2, 29.8, 23.5, 21.0, 19.5, 13.8. **HR-MS (APCI^+^):** m/z calculated for C_32_H_45_O_4_ 493.3312 [M+H]^+^, found m/z 493.3307. **HPLC:** 100% purity, RT = 30.73 min. Column: Kromasil 100-10-SIL (10 x 250 mm, 10 μm), Method: eluting with 30% ethyl acetate – 70% cyclohexane, isocratic, flow rate 1.5 mL/min at 25°C, injection volume 20 μL.

**(3*S*,17*R*)-3-hydroxy-3’,6’-dihydrospiro[5-androstene-17,2’-pyran]-5’-yl-[(methyl-azanediyl)bis (ethan-1-ol)] (ENT-A036).** To a solution of **ENT-A025** (40.0 mg, 0.10 mmol), in a mixture of anhydrous tetrahydrofuran/acetonitrile (1/1, 1.0 mL) diethanolamine (20.0 µL, 0.20 mmol) and *N,N*-diisopropylethylamine (52.2 µL, 0.30 mmol) were added and the reaction was stirred at 25°C for 48 h. To the reaction mixture water was added and the organic solvents were evaporated under reduced pressure. The residue was extracted with ethyl acetate and the organic phase washed with brine, dried over Na_2_SO_4_, and concentrated under reduced pressure. The residue was purified by FCC (elution solvent: dichloromethane/MeOH: 95/5) to afford **ENT-A036**, which was recrystallized from MeOH/diethyl ether to obtain pure **ENT-A036** as a white solid (12 mg, 26%). **Rf:** 0.26 (dichloromethane/MeOH: 95/5); **mp:** 151°C, decomposition; $\left[ a \right]_{D}^{24}=+42.86^{\circ}$(*c* = 0.0070 g/mL, CHCl_3_); **^1^H NMR (300 MHz, CD_3_OD):** *δ* 6.21 (bs, 1H), 5.40 – 5.33 (m, 1H), 4.40 (s, 2H), 4.17 (s, 2H), 4.04 (s, 4H), 3.78 – 3.57 (m, 4H), 3.47 – 3.36 (m, 1H), 2.53 (d, *J* = 17.7 Hz, 1H), 2.29 - 0.92 (m, 22H), 1.06 (s, 3H), 0.95 (s, 3H); **^13^C NMR (75 MHz, CD_3_OD):** *δ* 142.3, 135.1, 129.1, 122.2, 84.0, 72.4, 71.8, 67.7, 67.5, 67.0, 57.2, 52.4, 51.6, 47.0, 43.0, 38.6, 37.8, 34.2, 34.0, 33.9, 32.8, 32.3, 24.3, 22.0, 19.9, 14.1. **HR-MS (APCI^+^):** m/z calculated for C_28_H_46_O_4_N 460.3421 [M+H]^+^, found 460.3415; **HPLC:** 100% purity, RT = 45.82 min. Column: Fortis C18 (10 x 250 mm, 10 μm), Method: eluting with 10% water - 90% methanol, isocratic, flow rate 1.5 mL/min at 25°C, injection volume 20 μL.

**(3*S*,17*R*)-5'-(morpholinomethyl)-3’,6’-dihydrospiro[5-androstene-17,2’-pyran]-3-ol (ENT-A056).** A solution of **ENT-A025** (20.0 mg, 0.04 mmol) and morpholine (7.0 µL, 0.08 mmol) in anhydrous acetone (0.3 mL) was stirred at 25°C for 12h. The reaction mixture was evaporated *in vacuo*, and the residue partitioned between dichloromethane and water. The organic layer was washed with brine, dried over Na_2_SO_4_, and concentrated under reduced pressure. The residue was purified by FCC (elution solvent: ethyl acetate) to afford **ENT-A056**, as a white solid (11 mg, 62% yield). **Rf:** 0.40 (ethyl acetate); **mp:** 147°C, decomposition; $\left[ a \right]_{D}^{24}-7.30^{\circ}$(*c* = 0.00137 g/mL, CHCl_3_); **^1^H NMR (600 MHz, CDCl_3_):** *δ* 5.64 (bs, 1H), 5.35 – 5.32 (m, 1H), 4.17 and 4.11 (two d, ABq, *J_AB_* = 15.5 Hz, 2H), 3.68 -3.63 (m, 4H), 3.54 – 3.47 (m, 1H), 2.84 and 2.81 (two d, ABq, *J_AB_* = 12.3 Hz, 2H), 2.36 – 2.31 (m, 4H), 2.31 – 0.85 (m, 21H), 1.01 (s, 3H), 0.89 (s, 3H); **^13^C NMR (151 MHz, CDCl_3_):** *δ* 141.0, 133.3, 121.9, 121.5, 83.4, 71.9, 67.2, 65.1, 62.4, 53.7, 51.4, 50.3, 45.9, 42.4, 37.4, 36.7, 33.3, 33.1, 32.7, 31.9, 31.8, 31.3, 23.5, 21.0, 19.6, 13.8. **HR-MS (APCI^+^):** m/z calculated for C_28_H_44_NO_3_ 442.3316 [M+H]^+^, found 442.3318; **HPLC:** 100% purity, RT = 30.69 min. Column: Kromasil 100-10-SIL (10 x 250 mm, 10 μm), Method: eluting with 20% ethyl acetate – 80% toluene, isocratic, flow rate 2.1 mL/min at 25°C, injection volume 20 μL.

# NMR and HR-MS spectra


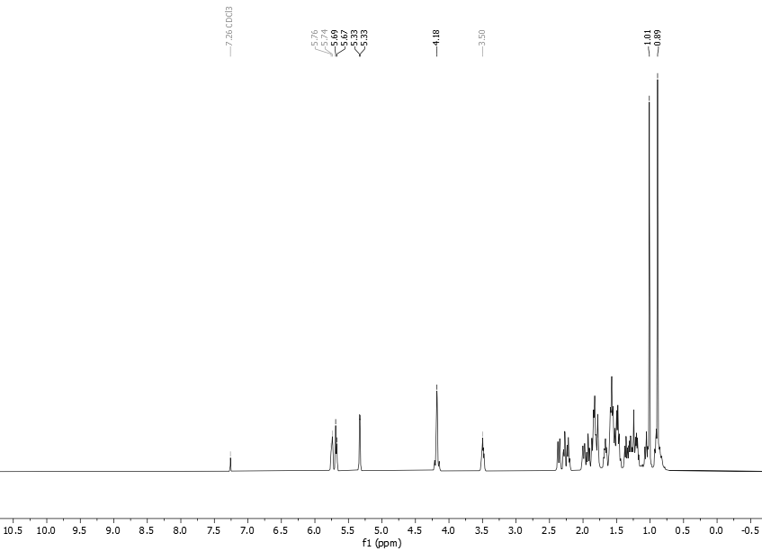


Supplementary Figure S1. ^1^H NMR spectrum (400 MHz, CDCl_3_) of ENT-A002.


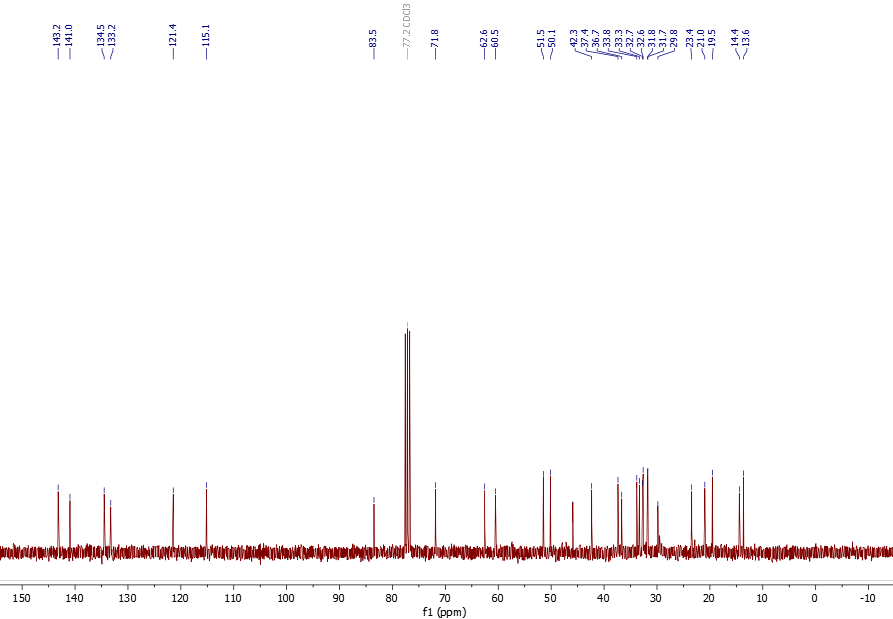


Supplementary Figure S2. ^13^C NMR spectrum (151 MHz, CDCl_3_) of ENT-A002.


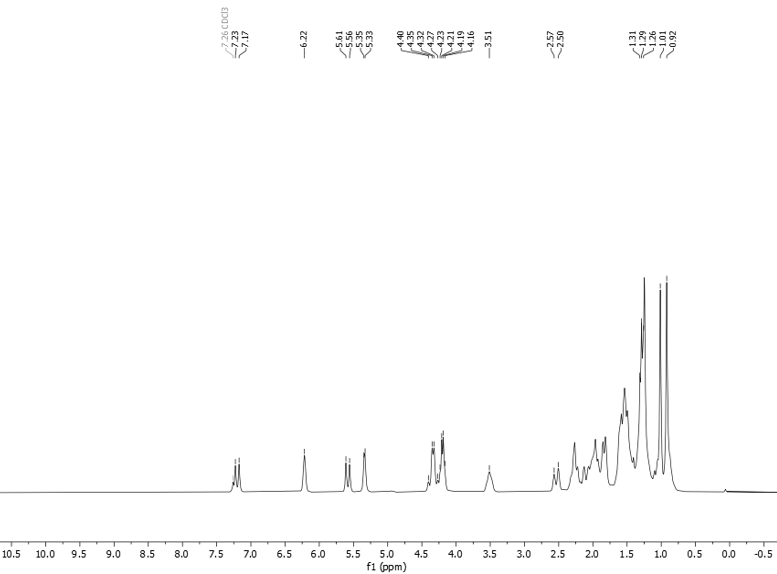


Supplementary Figure S3. ^1^H NMR spectrum (600 MHz, CDCl_3_) of ENT-A007.


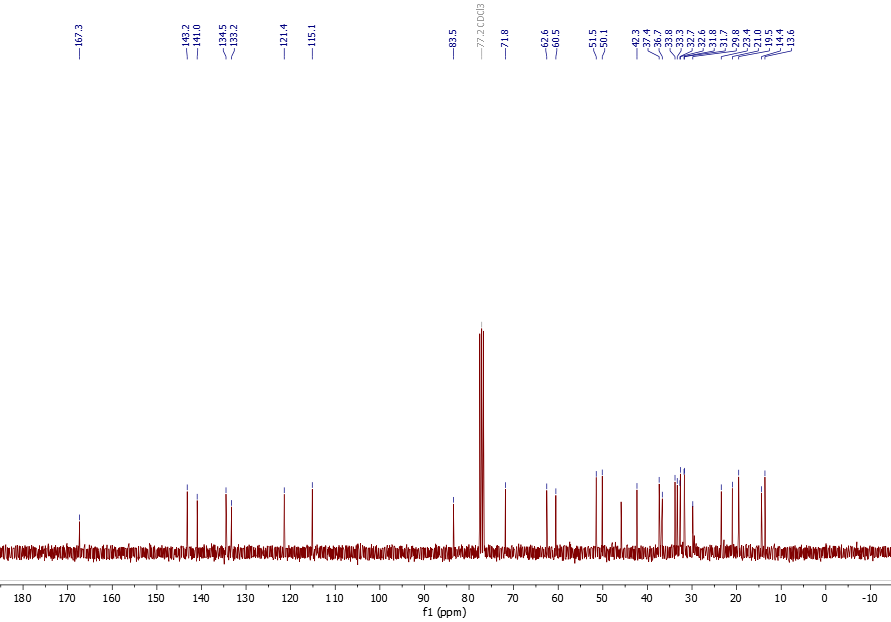


Supplementary Figure S4. ^13^C NMR såpectrum (75 MHz, CDCl_3_) of ENT-A007.

Supplementary Figure S5. APCI-HR-MS spectrum of ENT-A007.


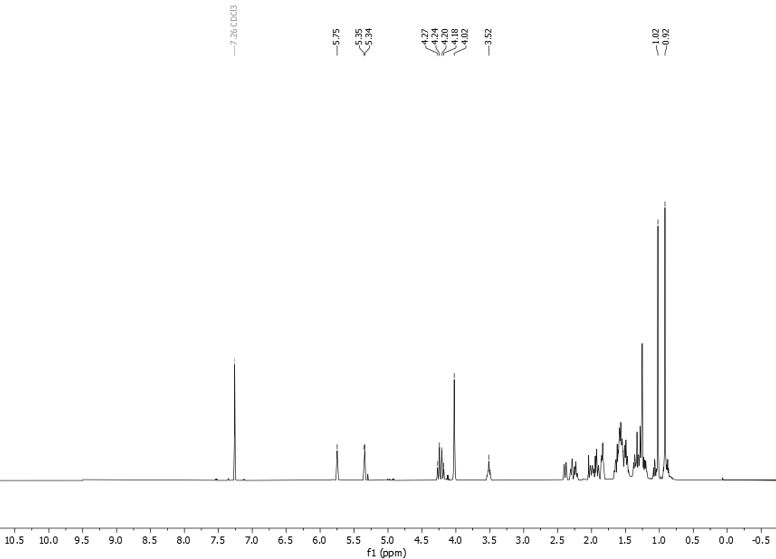


Supplementary Figure S6. ^1^H NMR spectrum (600 MHz, CDCl_3_) of ENT-A008.


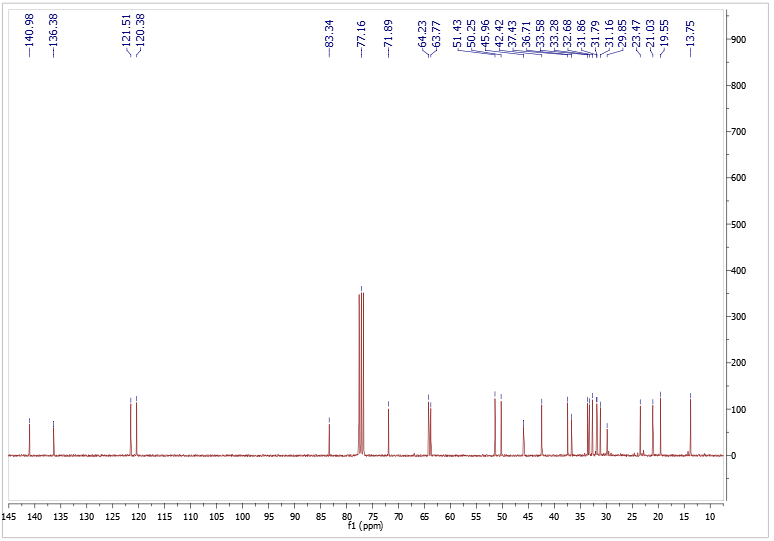


Supplementary Figure S7. ^13^C NMR spectrum (75 MHz, CDCl_3_) of ENT-A008.


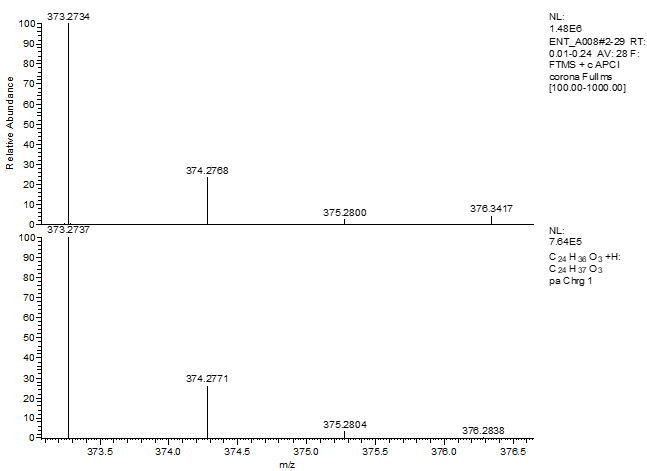


Supplementary Figure S8. APCI-HR-MS spectrum of ENT-A008.


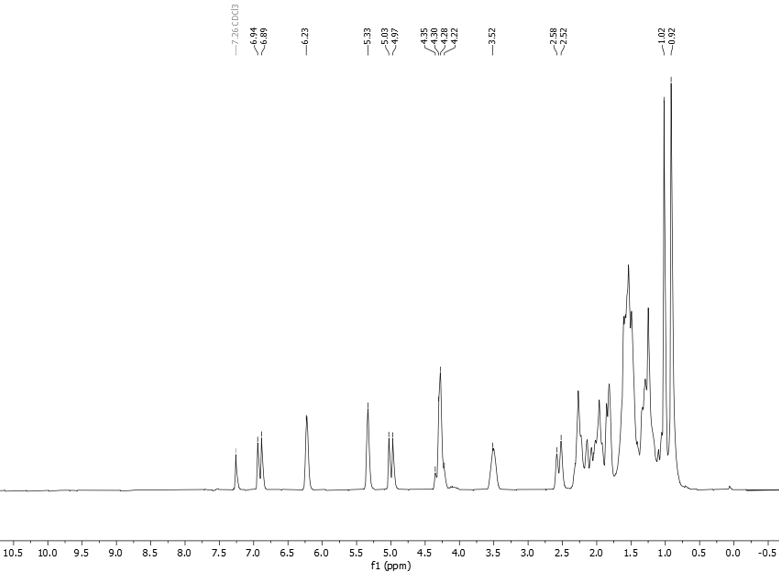


Supplementary Figure S9. ^1^H NMR spectrum (600 MHz, CDCl_3_) of ENT-A009.


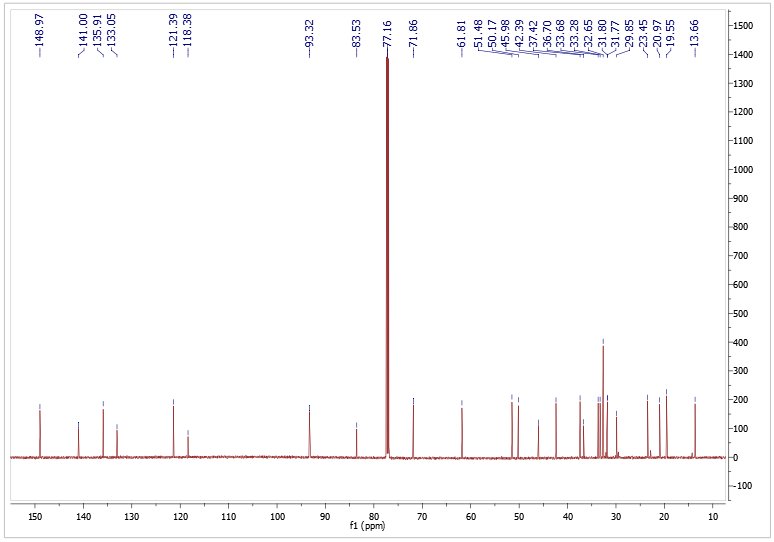


Supplementary Figure S10. ^13^C NMR spectrum (151 MHz, CDCl_3_) of ENT-A009.

Supplementary Figure S11. APCI-HR-MS spectrum of ENT-A009.


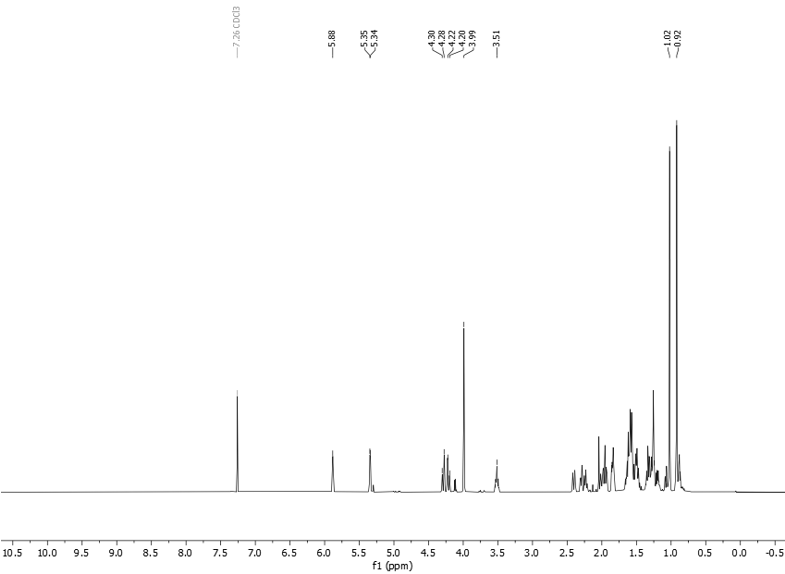


Supplementary Figure S12. ^1^H NMR spectrum (600 MHz, CDCl_3_) of ENT-A025.


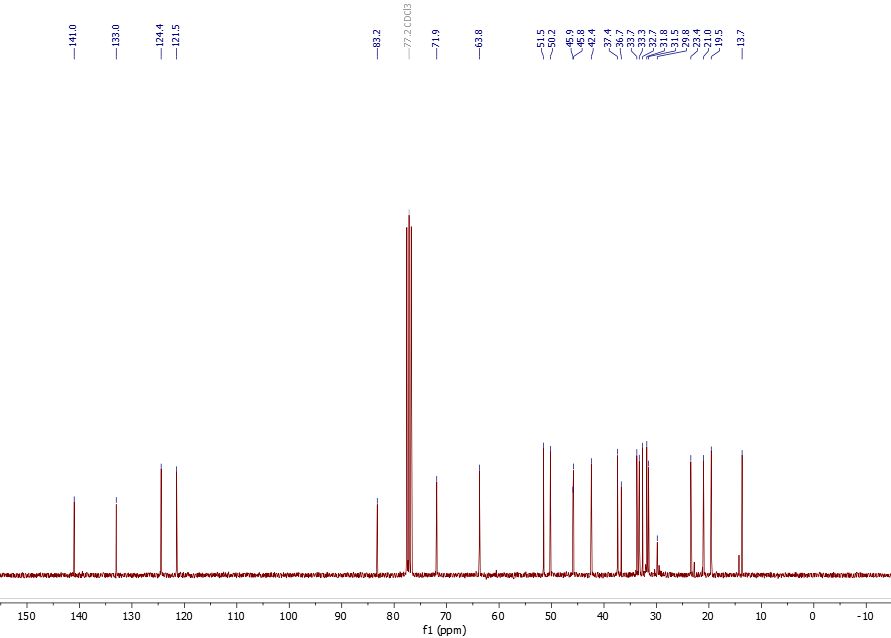


Supplementary Figure S13. ^13^C NMR spectrum (75 MHz, CDCl_3_) of ENT-A025.

Supplementary Figure S14. APCI-HR-MS spectrum (MeOH) of ENT-A025.


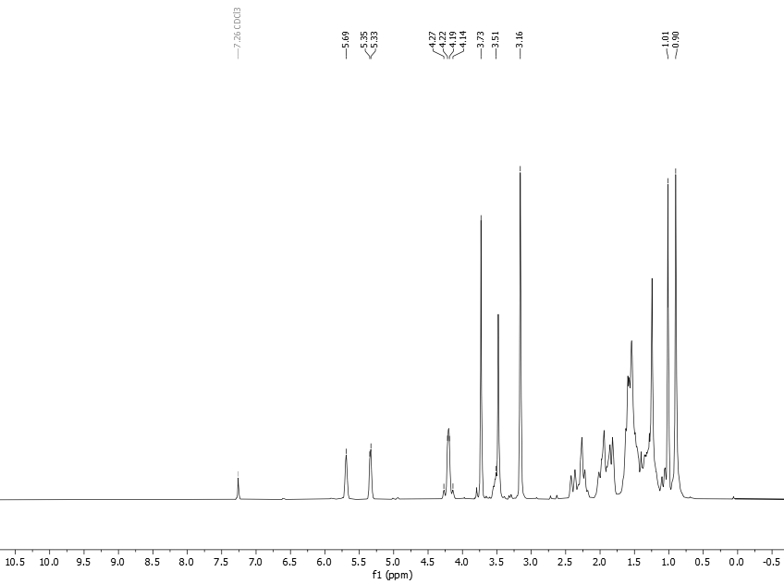


Supplementary Figure S15. ^1^H NMR spectrum (300 MHz, CDCl_3_) of ENT-A026.


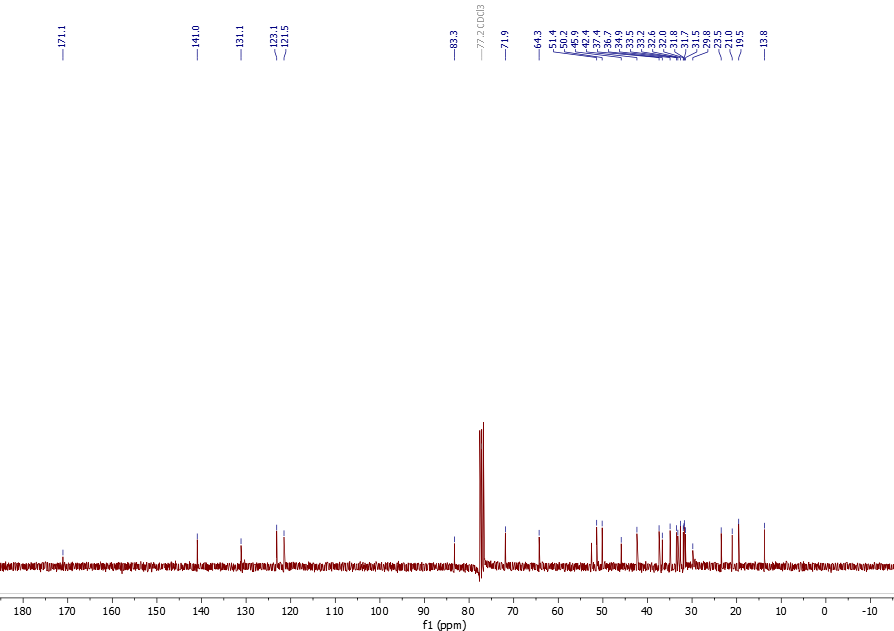


Supplementary Figure S16. ^13^C NMR spectrum (75 MHz, CDCl_3_) of ENT-A026.

Supplementary Figure S17. APCI-HR-MS spectrum of ENT-A026.


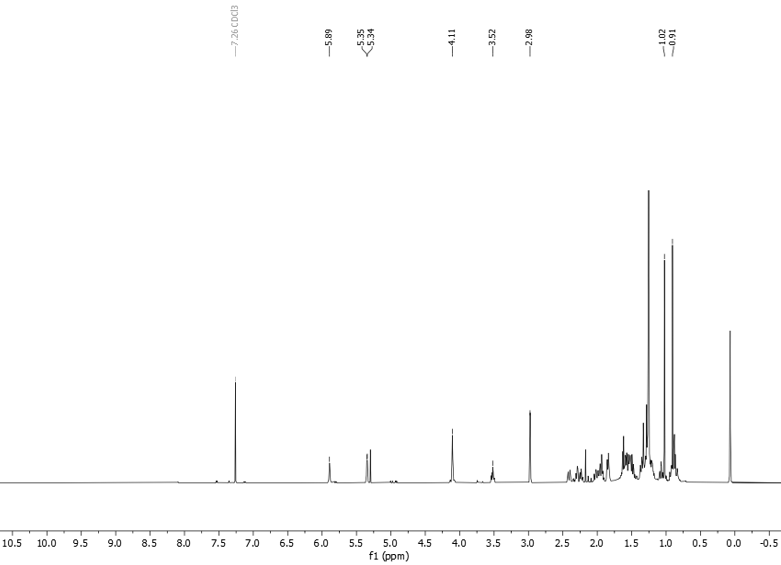


Supplementary Figure S18. ^1^H NMR spectrum (600 MHz, CDCl_3_) of ENT-A033.


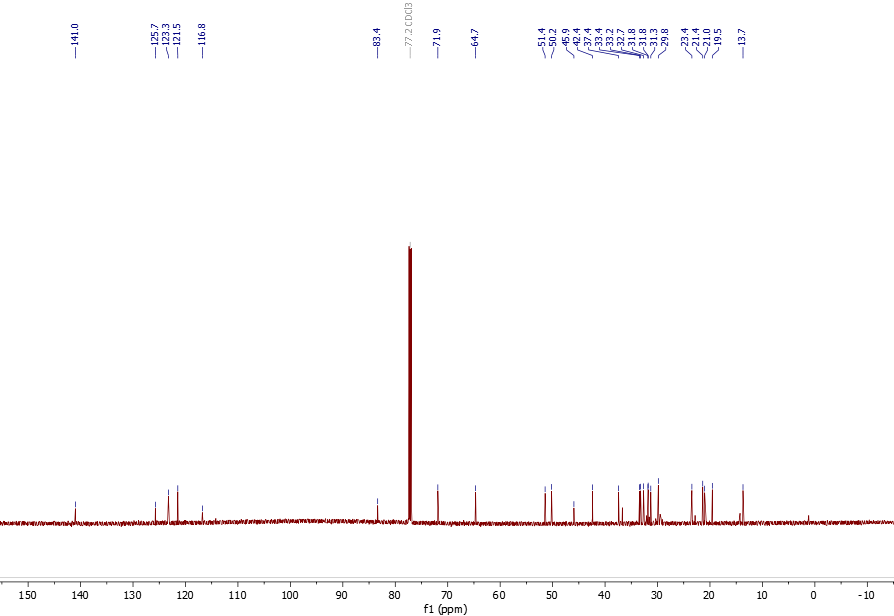


Supplementary Figure S19. ^13^C NMR spectrum (151 MHz, CDCl_3_) of ENT-A033.

Supplementary Figure S20. APCI-HR-MS spectrum of ENT-A033.


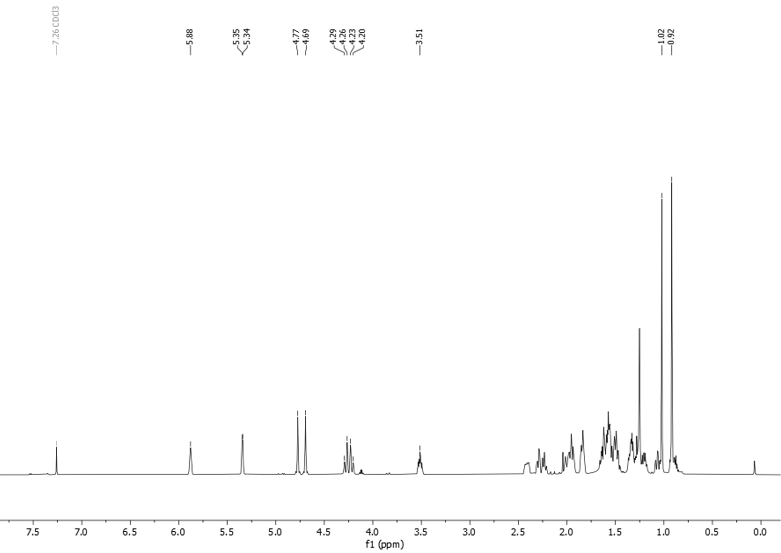


Supplementary Figure S21. ^1^H NMR spectrum (600 MHz, CDCl_3_) of ENT-A034.


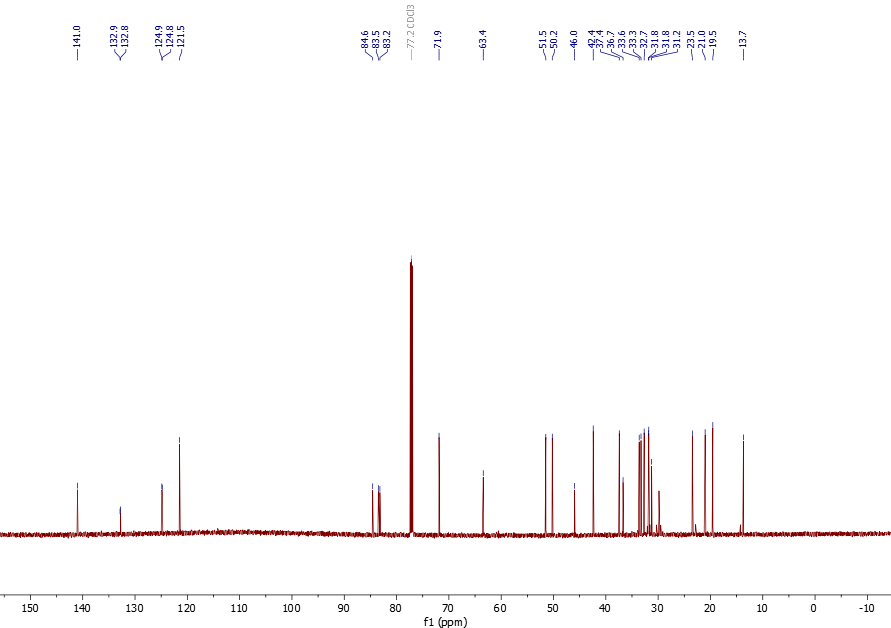


Supplementary Figure S22. ^13^C NMR spectrum (151 MHz, CDCl_3_) of ENT-A034.


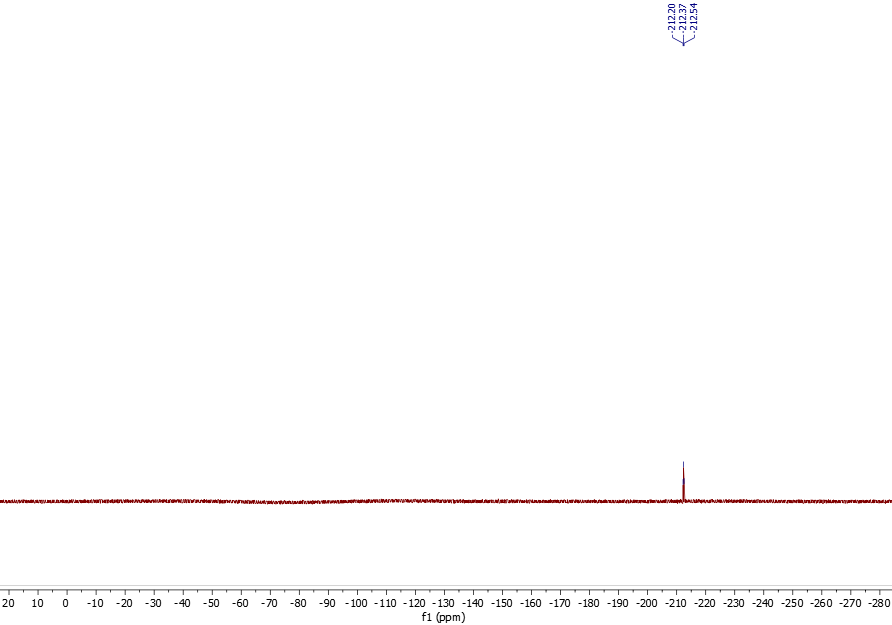


Supplementary Figure S23. ^19^F NMR spectrum (282 MHz, CDCl_3_) of ENT-A034.

Supplementary Figure S24. APCI-HR-MS spectrum of ENT-A034.


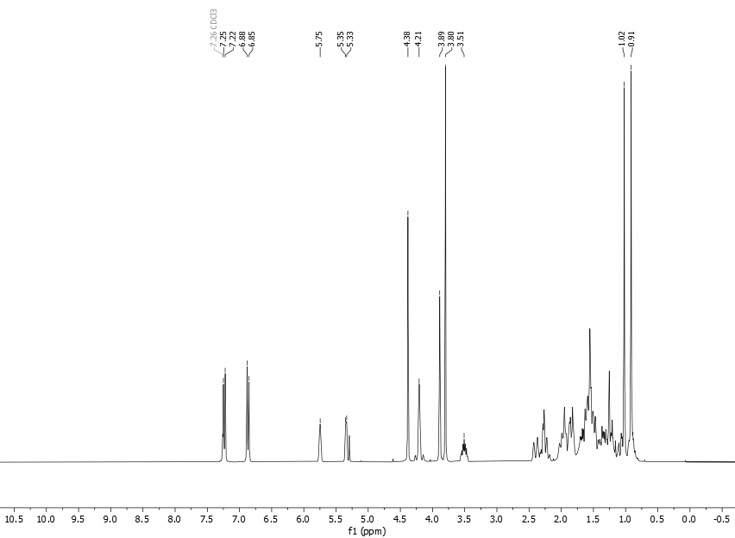


Supplementary Figure S25. ^1^H NMR spectrum (300 MHz, CDCl_3_) of ENT-A035.


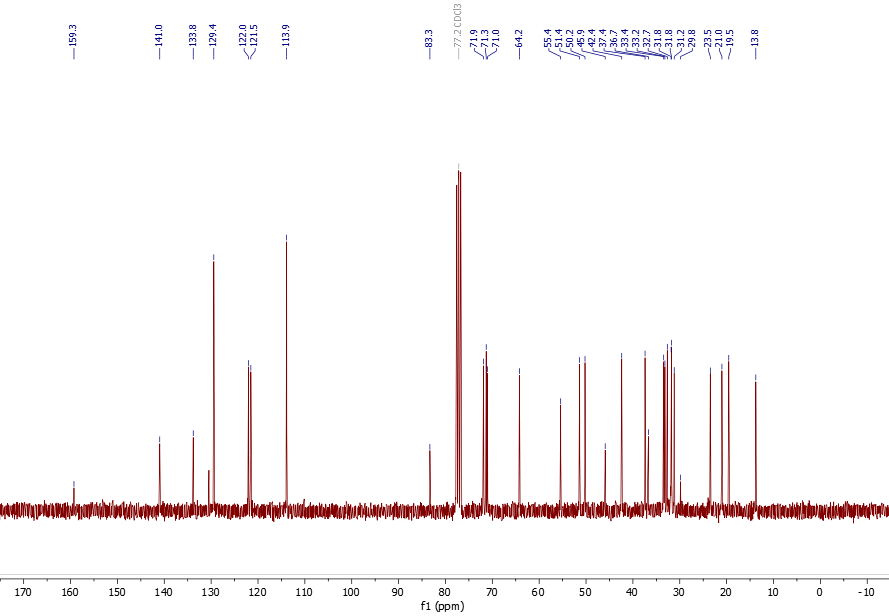


Supplementary Figure S26. ^13^C NMR Spectrum (75 MHz, CDCl_3_) of ENT-A035.


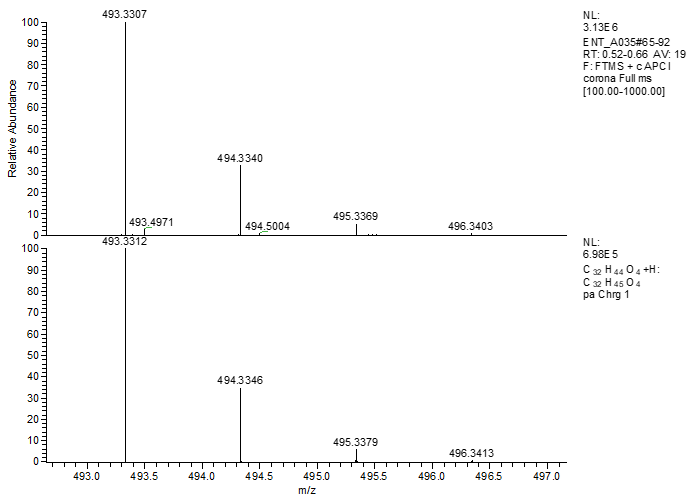


Supplementary Figure S27. APCI-HR-MS spectrum of ENT-A035.


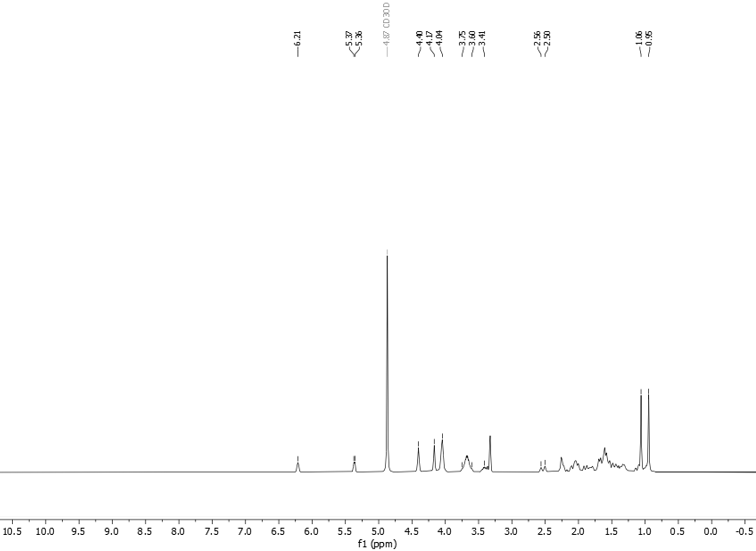


Supplementary Figure S28. ^1^H NMR spectrum (300 MHz, CD_3_OD) of ENT-A036.


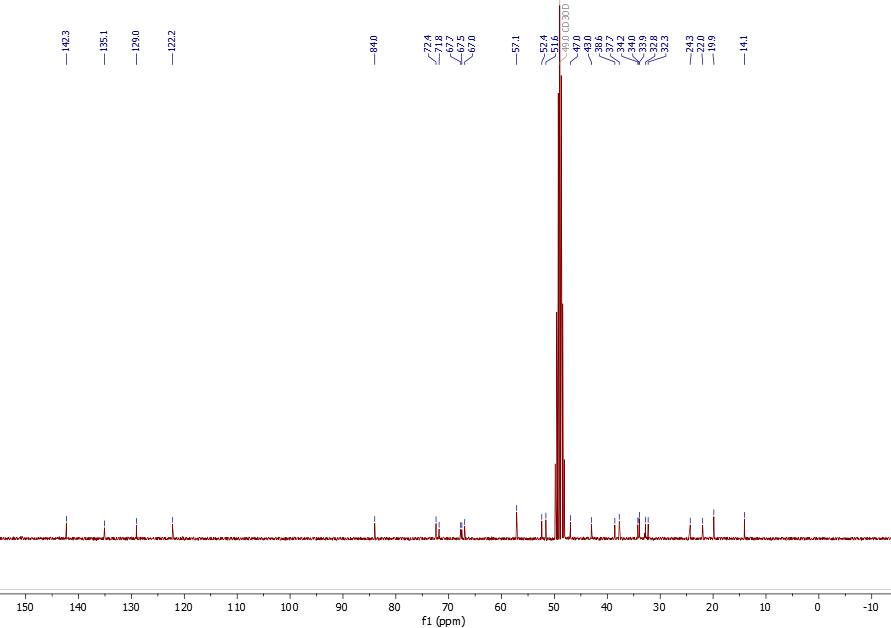


Supplementary Figure S29. ^13^C NMR spectrum (75 MHz, CD_3_OD) of ENT-A036.

Supplementary Figure S30. APCI-HR-MS spectrum of ENT-A036.


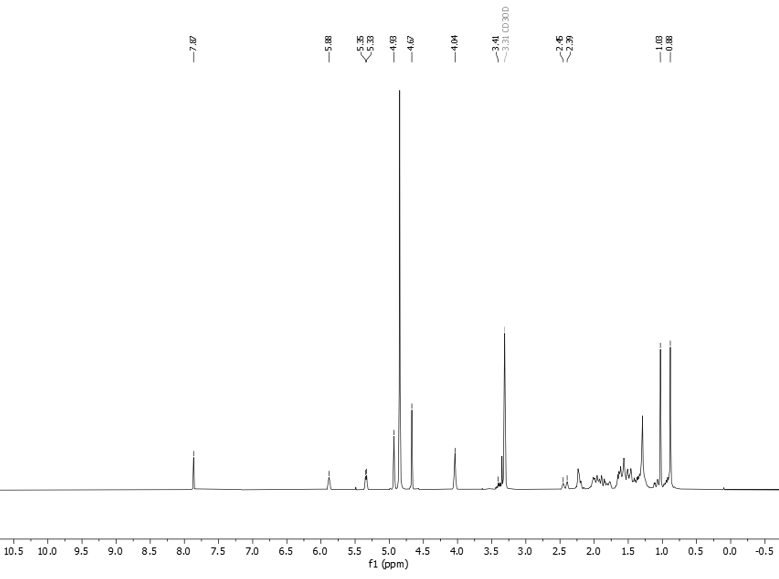


Supplementary Figure S31. ^1^H NMR spectrum (300 MHz, CD_3_OD) of ENT-A037.


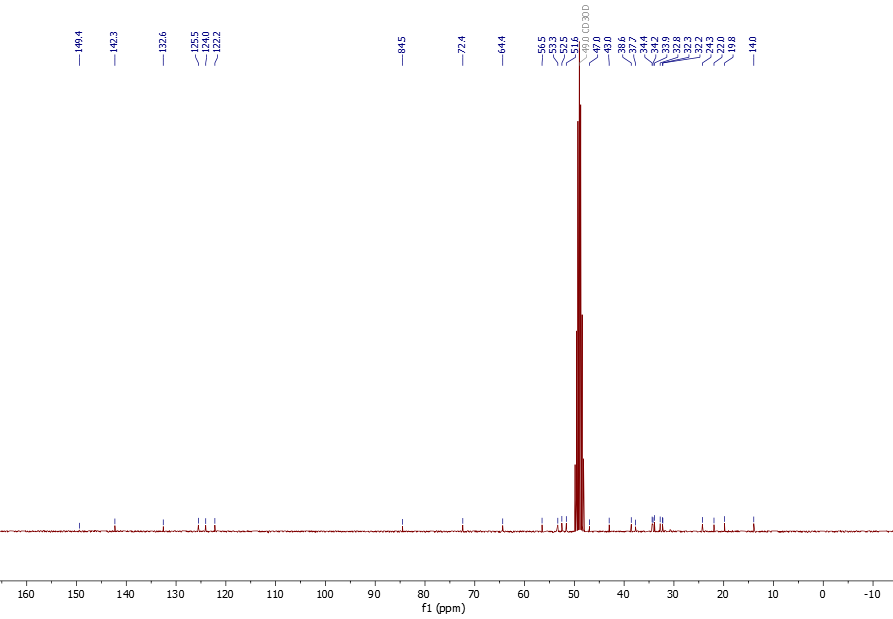


Supplementary Figure S32. ^13^C NMR spectrum (75 MHz, CD_3_OD) of ENT-A037.

Supplementary Figure S33. APCI-HR-MS spectrum of ENT-A037.


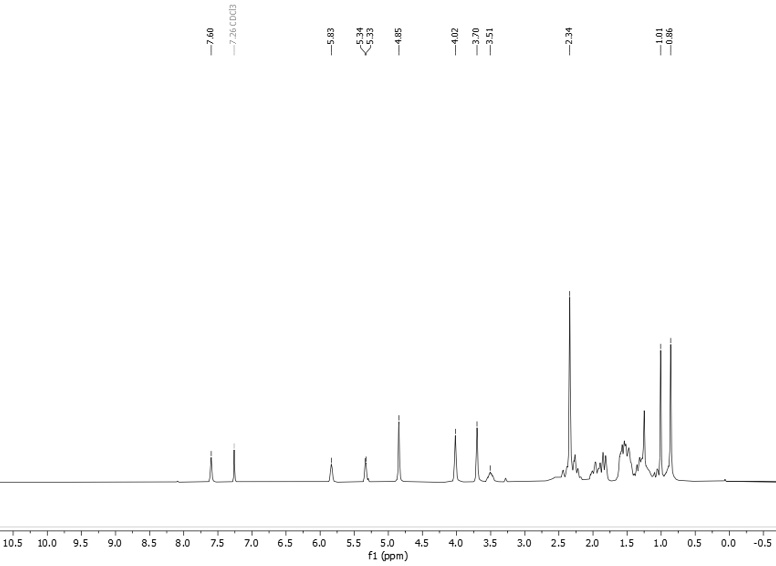


Supplementary Figure S34. ^1^H NMR spectrum (300 MHz, CDCl_3_) of ENT-A046.


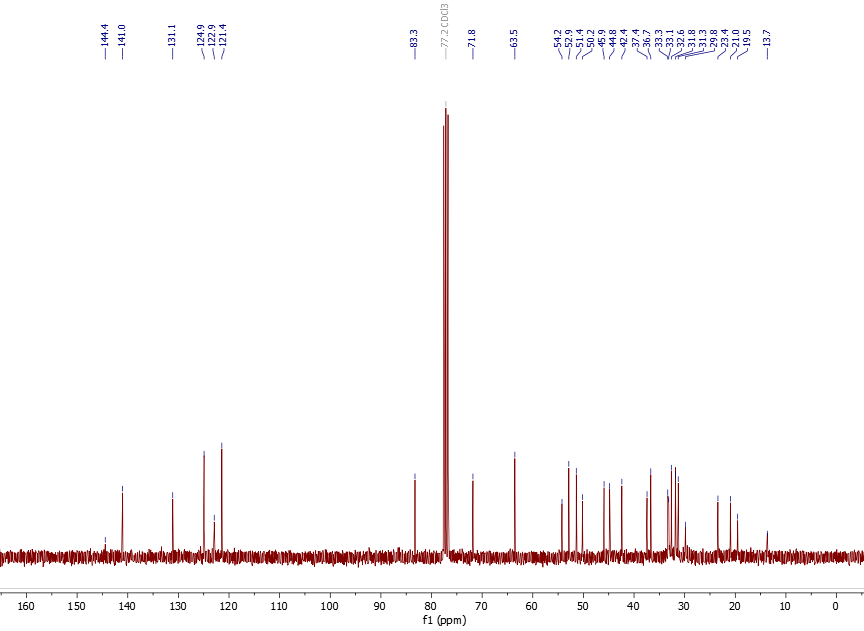


Supplementary Figure S35. ^13^C NMR spectrum (75 MHz, CDCl_3_) of ENT-A046.

Supplementary Figure S36. APCI-HR-MS spectrum of ENT-A046.


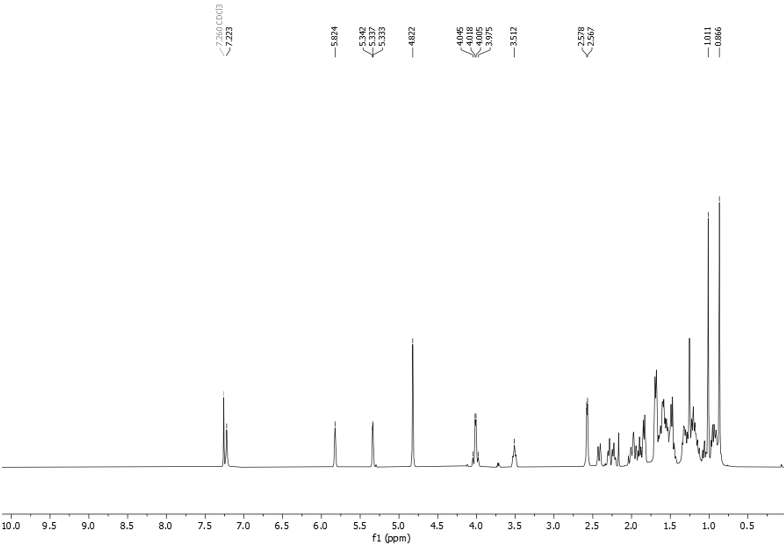


Supplementary Figure S37. ^1^H NMR spectrum (600 MHz, CDCl_3_) of ENT-A047.


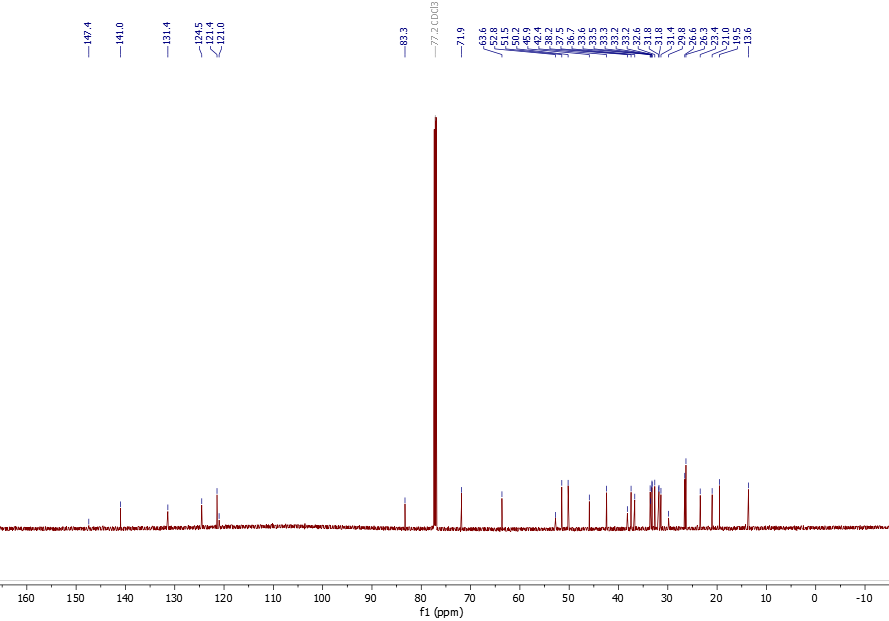


Supplementary Figure S38. ^13^C NMR spectrum (151 MHz, CDCl_3_) of ENT-A047.

Supplementary Figure S39. APCI-HR-MS spectrum of ENT-A047.


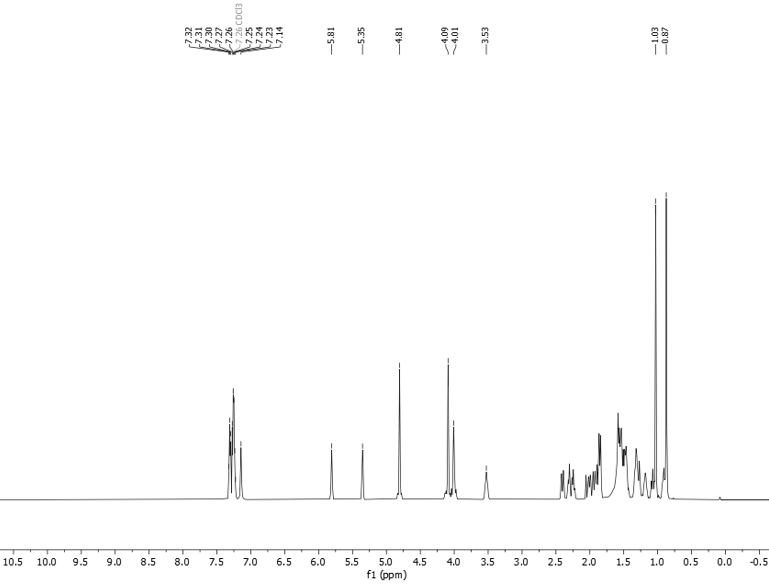


Supplementary Figure S40. ^1^H NMR spectrum (600 MHz, CDCl_3_) of ENT-A055.


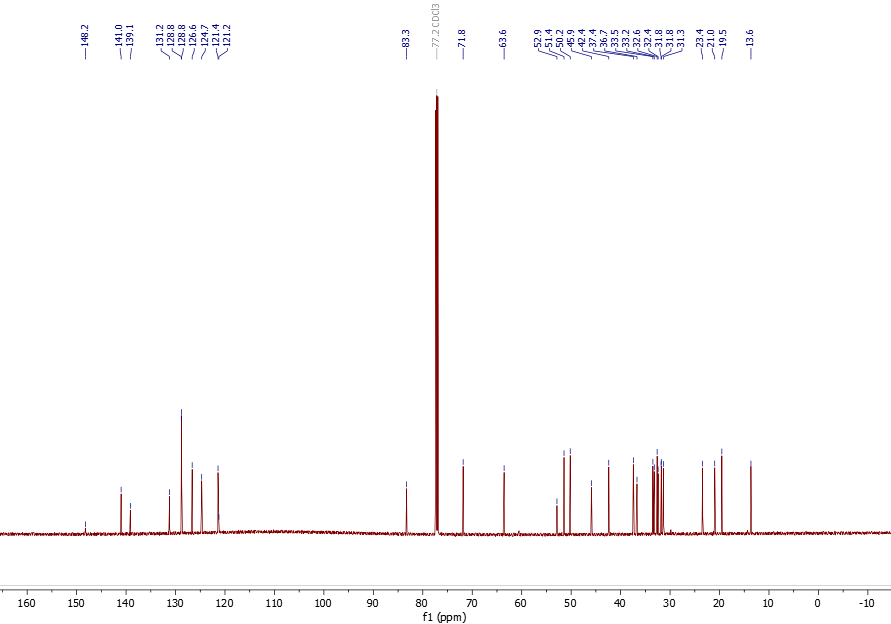


Supplementary Figure S41. ^13^C NMR spectrum (151 MHz, CDCl_3_) of ENT-A055.

Supplementary Figure S42. APCI-HR-MS spectrum of ENT-A055.


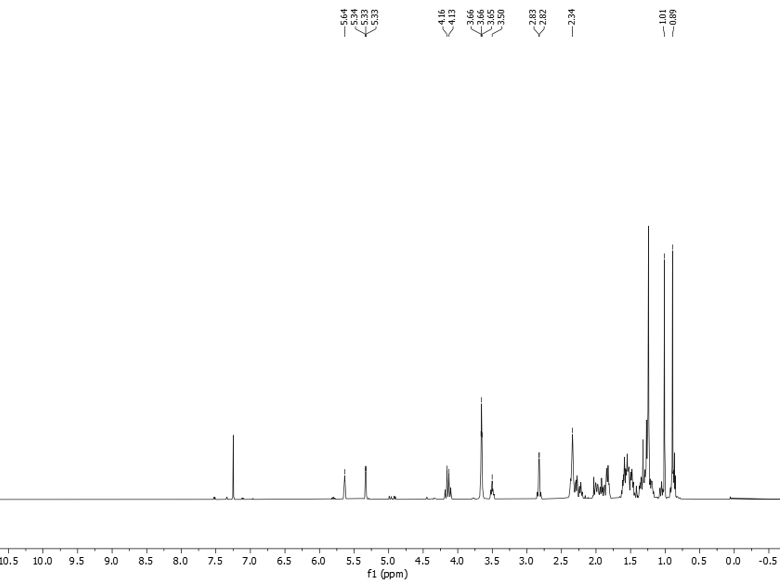


Supplementary Figure S43. ^1^H NMR spectrum (600 MHz, CDCl_3_) of ENT-A056.


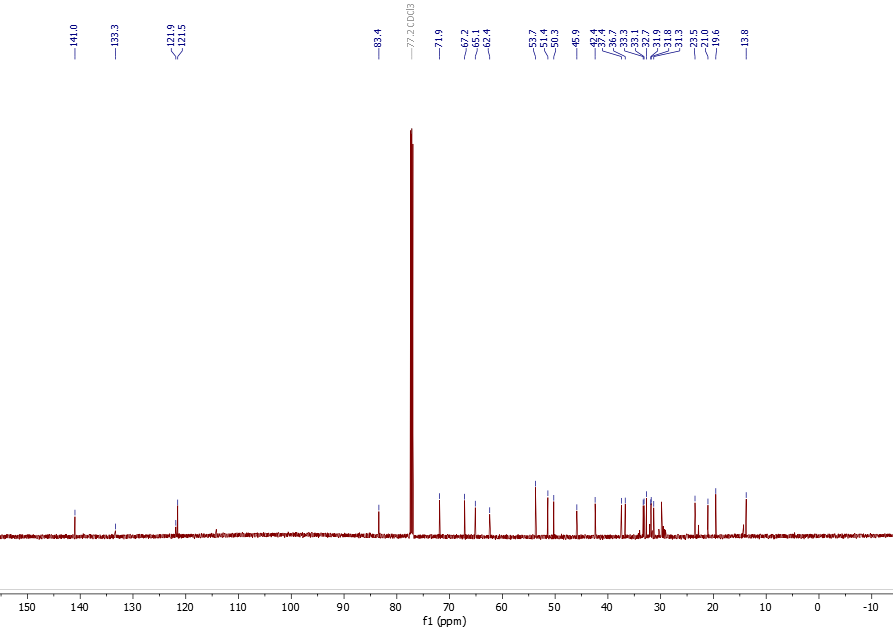


Supplementary Figure S44. ^13^C NMR spectrum (151 MHz, CDCl_3_) of ENT-A056.

Supplementary Figure S45. APCI-HR-MS spectrum of ENT-A056.


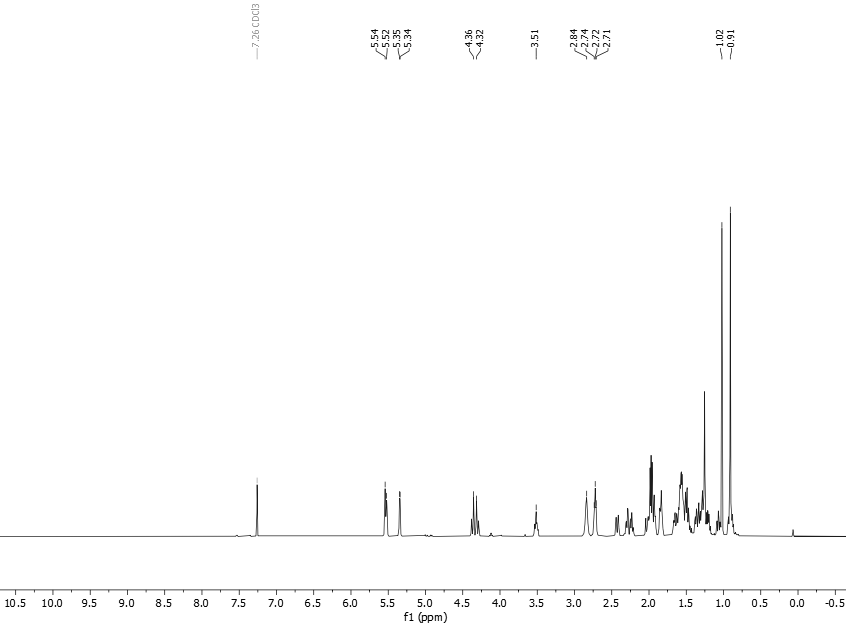


Supplementary Figure 46. ^1^H NMR spectrum (600 MHz, CDCl_3_) of ENT-A065.


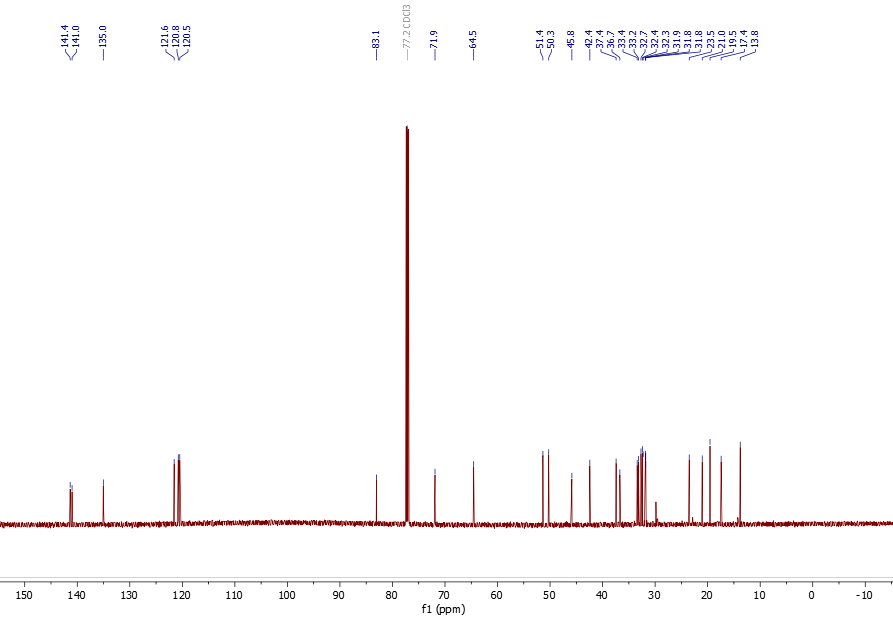


Supplementary Figure S47. ^13^C NMR spectrum (151 MHz, CDCl_3_) of ENT-A065.

Supplementary Figure S48. APCI-HR-MS spectrum of ENT-A065.


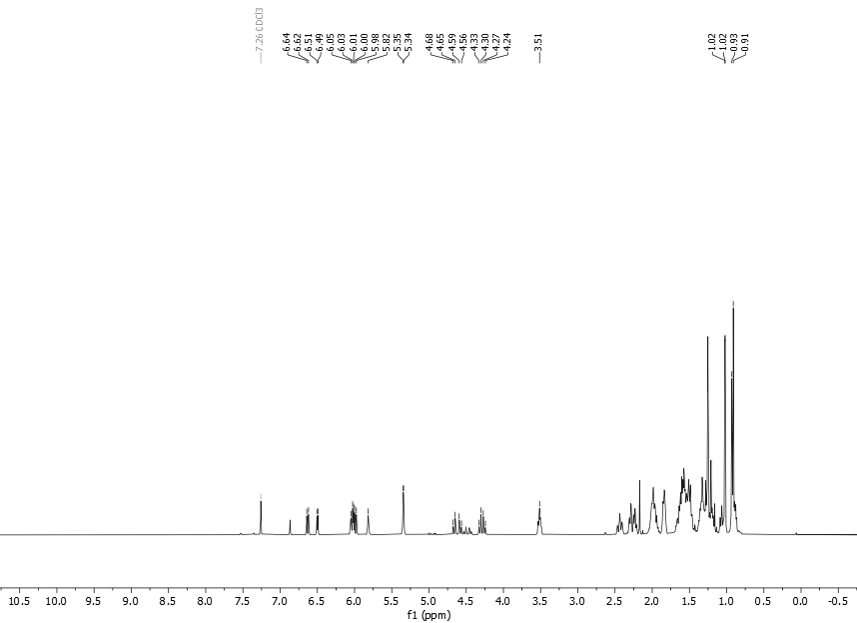


Supplementary Figure S49. ^1^H NMR spectrum (600 MHz, CDCl_3_) of ENT-A066.


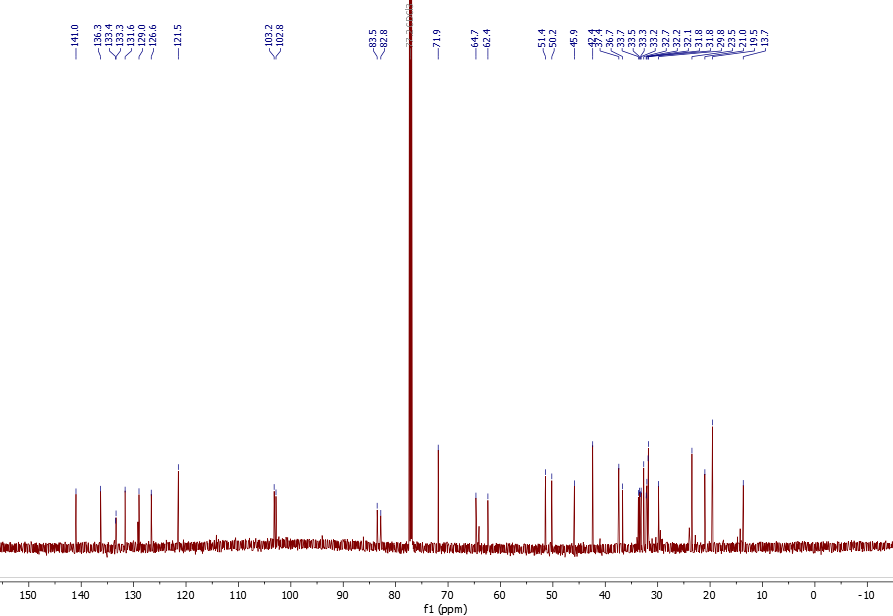


Supplementary Figure S50. ^13^C NMR spectrum (151 MHz, CDCl_3_) of ENT-A066.

Supplementary Figure S51. APCI-HR-MS spectrum of ENT-A066.


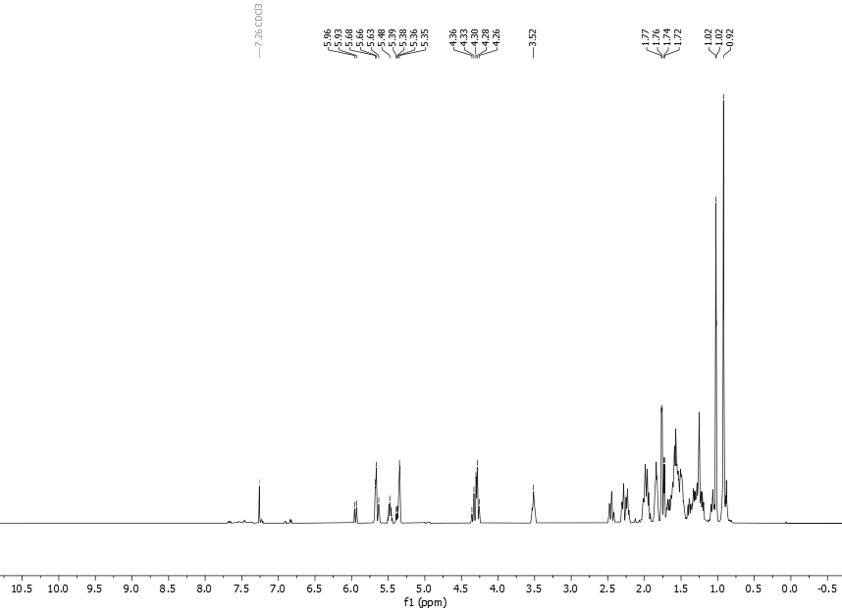


Supplementary Figure S52. ^1^H NMR spectrum (600 MHz, CDCl_3_) of ENT-A068.


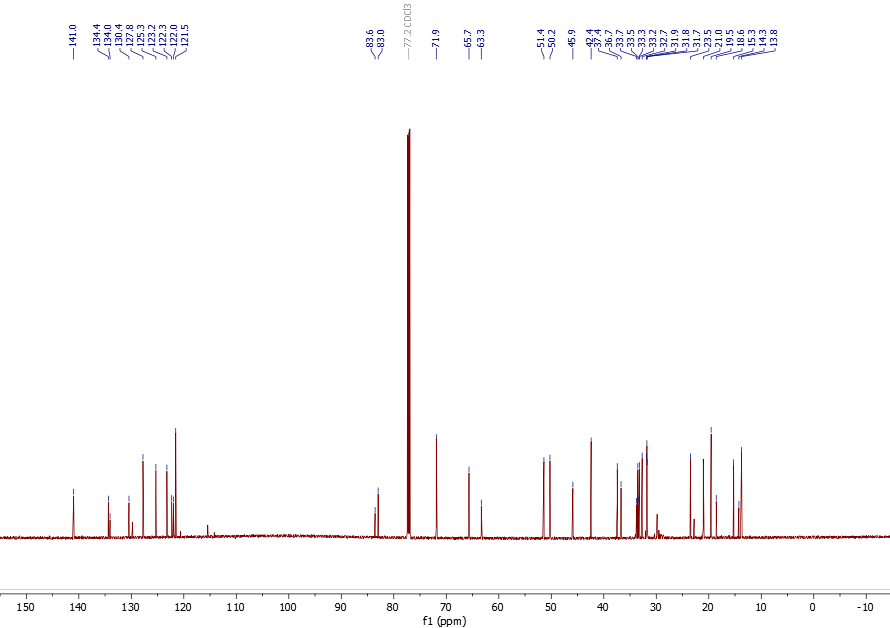


Supplementary Figure S53. ^13^C NMR spectrum (151 MHz, CDCl_3_) of ENT-A068.

Supplementary Figure S54. APCI-HR-MS spectrum of ENT-A068.


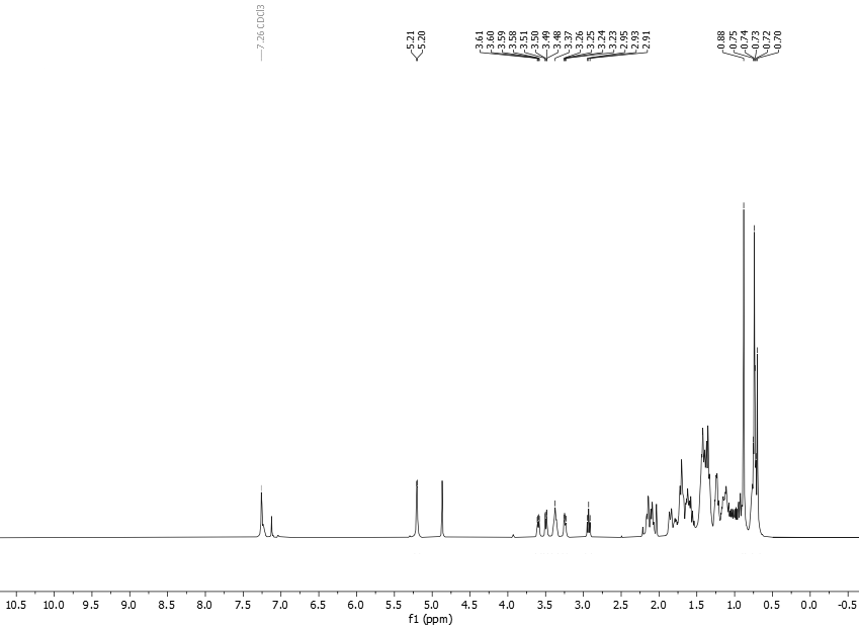


Supplementary Figure S55. ^1^H NMR spectrum (600 MHz, CDCl_3_) of ENT-A069.


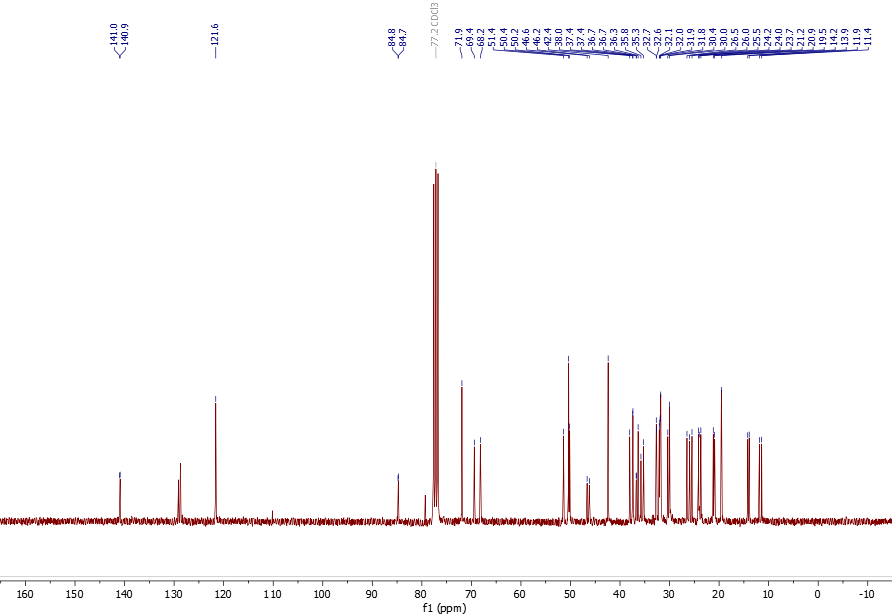


Supplementary Figure S56. ^13^C NMR spectrum (75 MHz, CDCl_3_) of ENT-A069.

Supplementary Figure S57. APCI-HR-MS spectrum of ENT-A069.


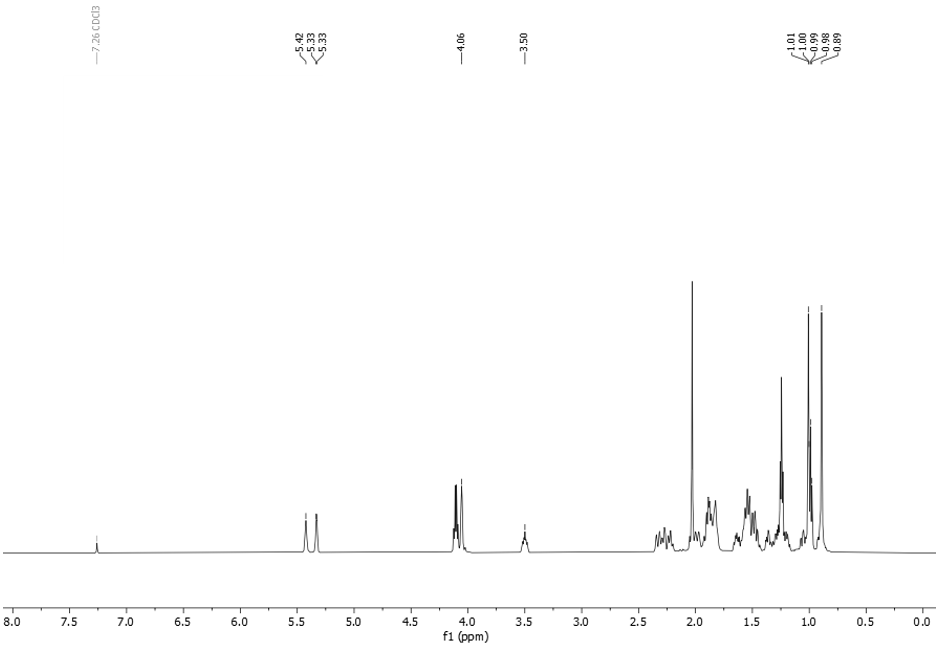


Supplementary Figure S58. ^1^H NMR spectrum (600 MHz, CDCl_3_) of ENT-A070.


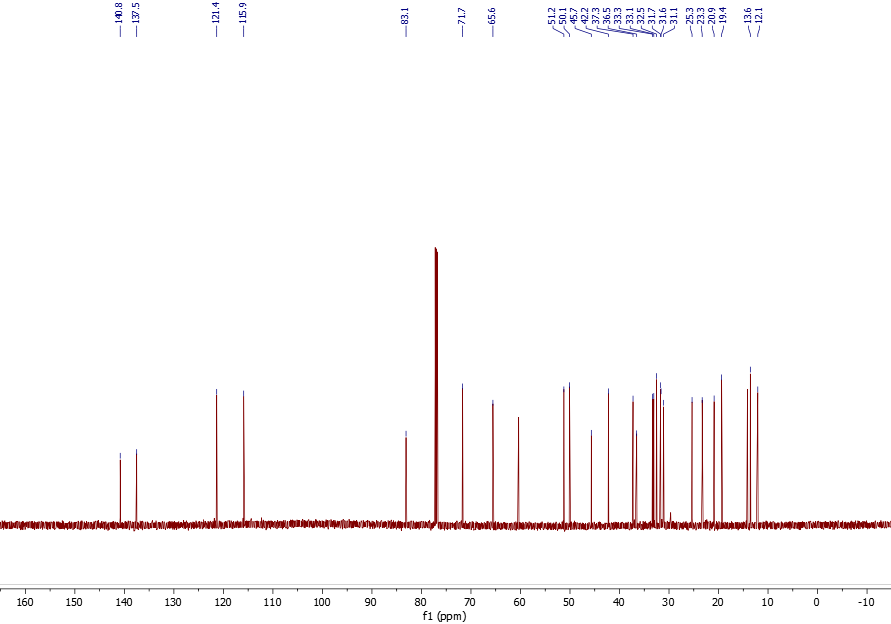


Supplementary Figure S59. ^13^C NMR spectrum (151 MHz, CDCl_3_) of ENT-A070.

Supplementary Figure S60. APCI-HR-MS spectrum of ENT-A070.


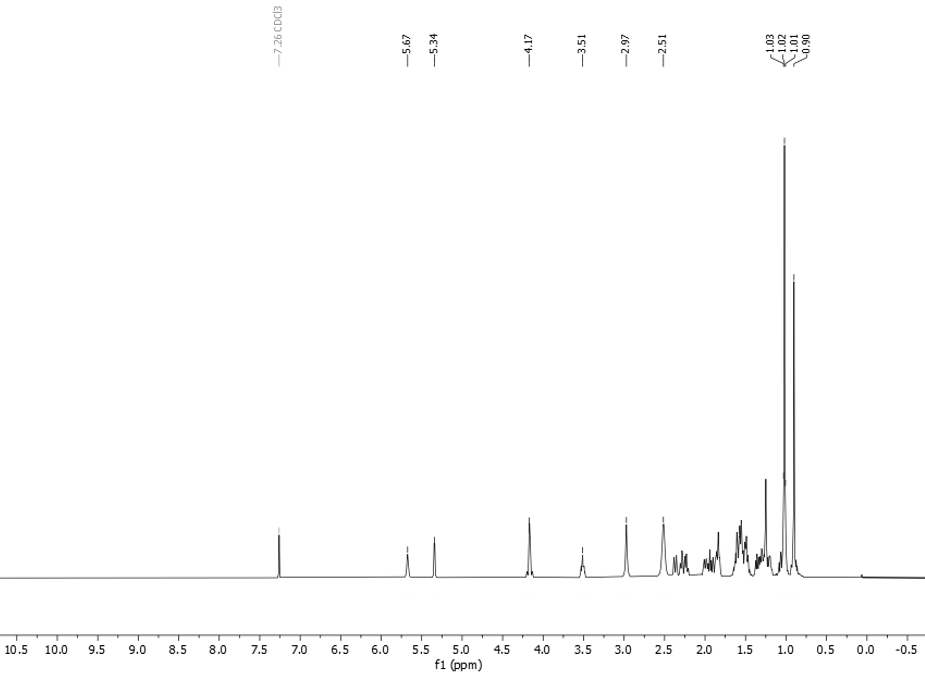


Supplementary Figure S61. ^1^H NMR spectrum (600, CDCl_3_) of ENT-A075.


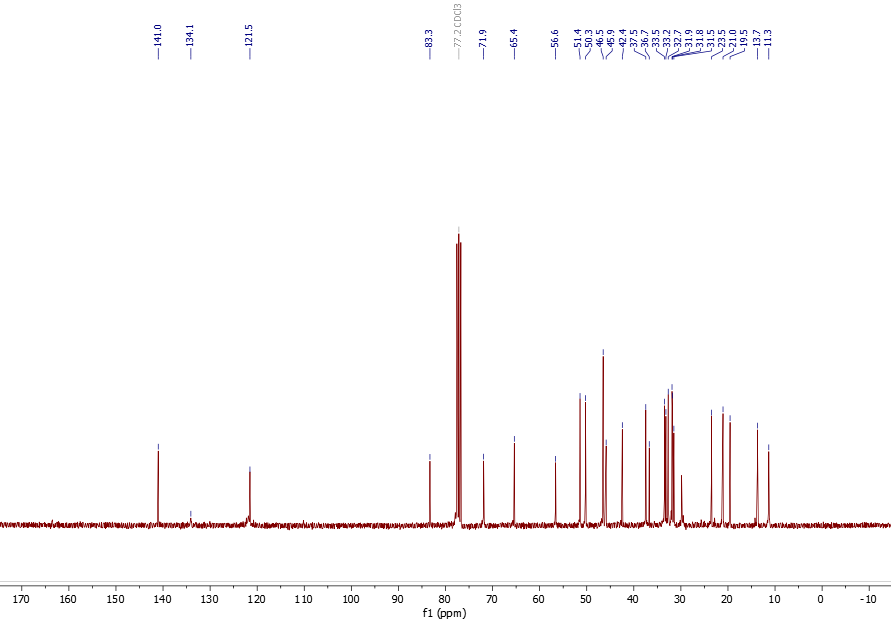


Supplementary Figure S62. ^13^C NMR spectrum (75 MHz, CDCl_3_) of ENT-A075.

Supplementary Figure S63. APCI-HR-MS spectrum of ENT-A075.


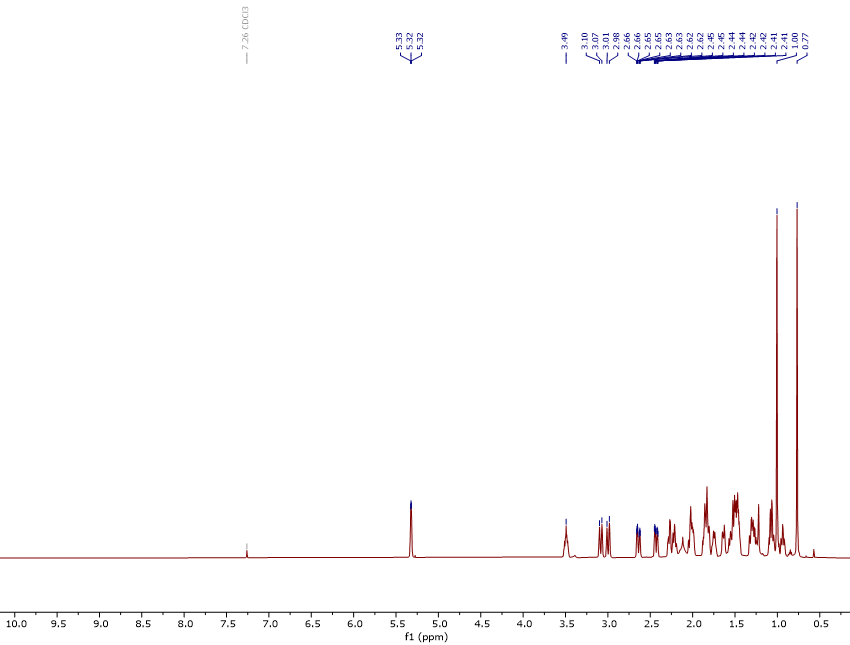


Supplementary Figure S64. ^1^H NMR spectrum (600 MHz, CDCl_3_) of ENT-A076.


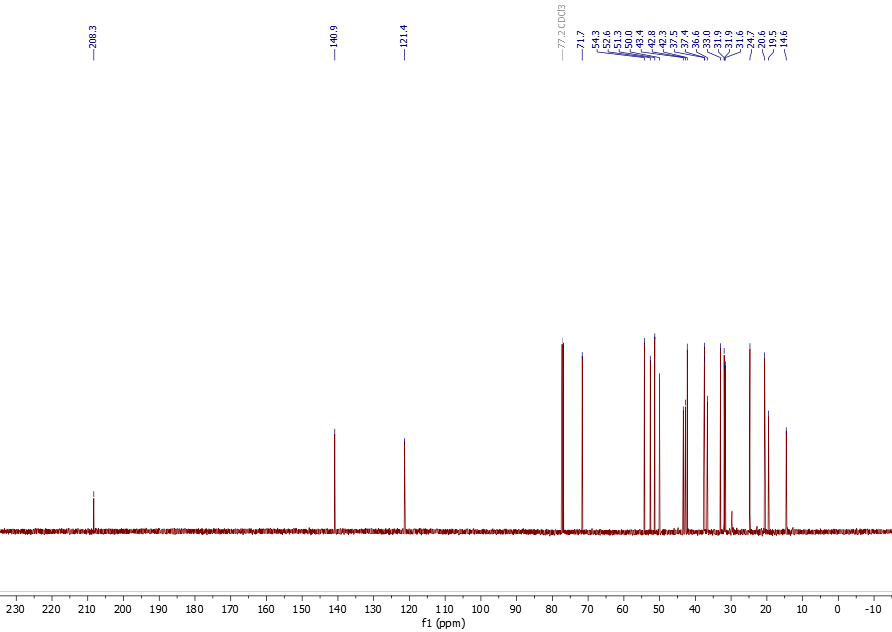


Supplementary Figure S65. ^13^C NMR spectrum (151 MHz, CDCl_3_) of ENT-A076.

Supplementary Figure S66. APCI-HR-MS spectrum of ENT-A076.


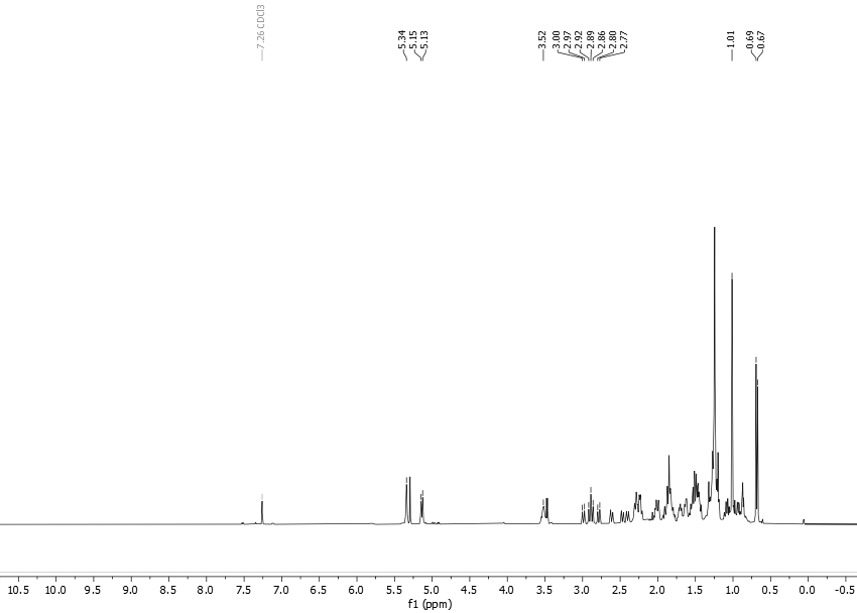


Supplementary Figure S67. ^1^H NMR spectrum (600 MHz, CDCl_3_) of ENT-A077.


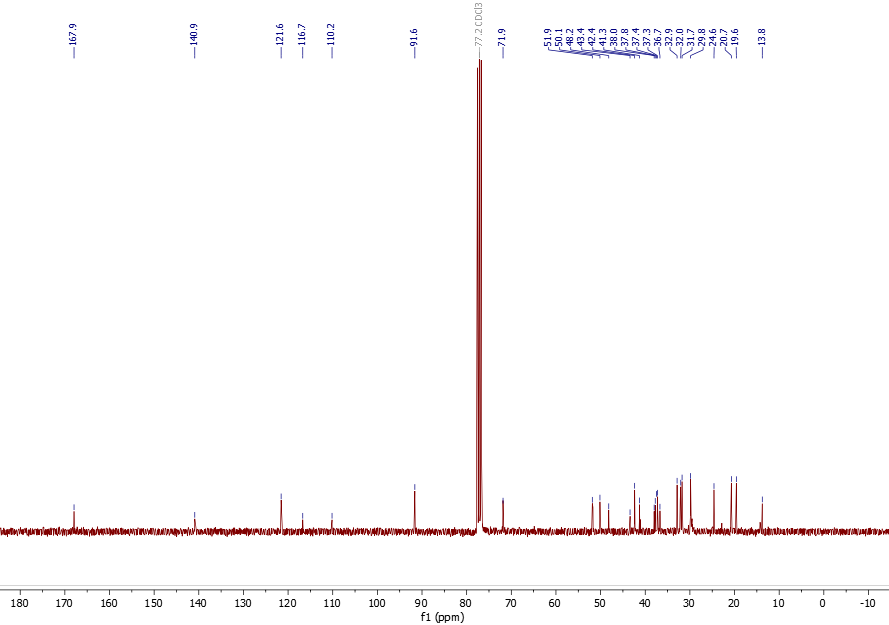


Supplementary Figure S68. ^13^C NMR spectrum (75 MHz, CDCl_3_) of ENT-A077.

Supplementary Figure S69. APCI-HR-MS spectrum of ENT-A077.


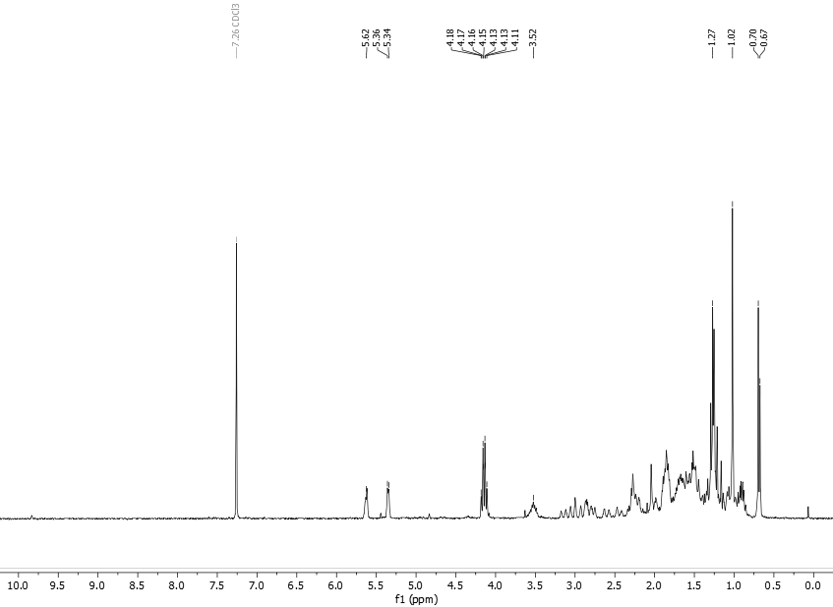


Supplementary Figure S70. ^1^H NMR spectrum (300 MHz, CDCl_3_) of ENT-A079.


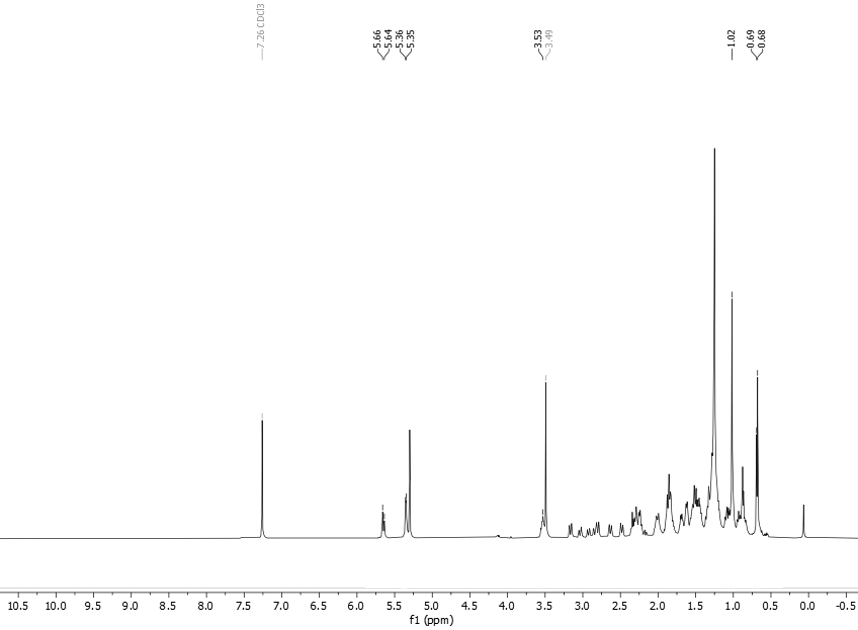


Supplementary Figure S71. ^1^H NMR spectrum (600 MHz, CDCl_3_) of ENT-A080.


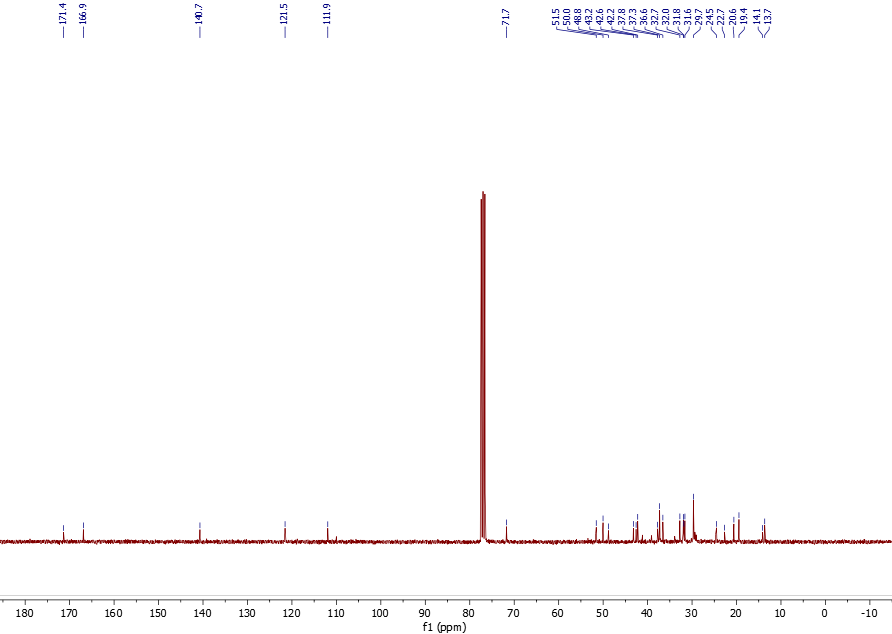


Supplementary Figure S72. ^13^C NMR spectrum (151 MHz, CDCl_3_) of ENT-A080.

Supplementary Figure S73. APCI-HR-MS spectrum (MeOH) of ENT-A080.


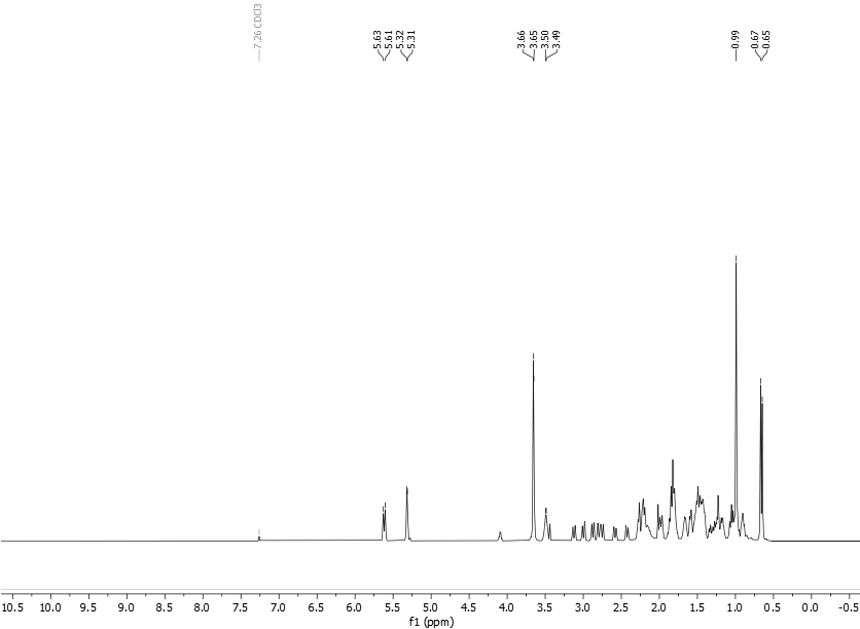


Supplementary Figure S74. ^1^H NMR spectrum (600 MHz, CDCl_3_) of ENT-A087.


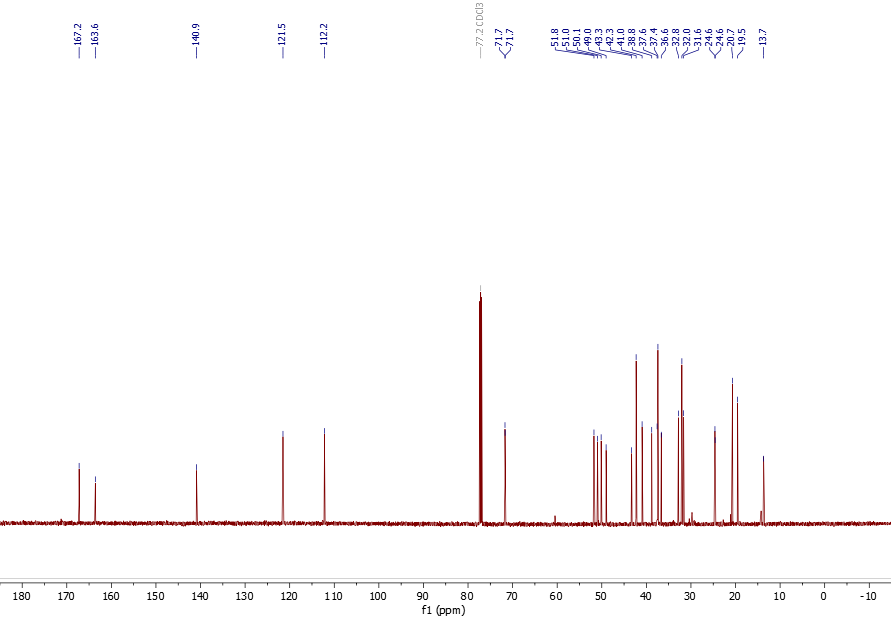


Supplementary Figure S75. ^13^C NMR spectrum (75 MHz, CDCl_3_) of ENT-A087.

Supplementary Figure S76. APCI-HR-MS spectrum of ENT-A087.


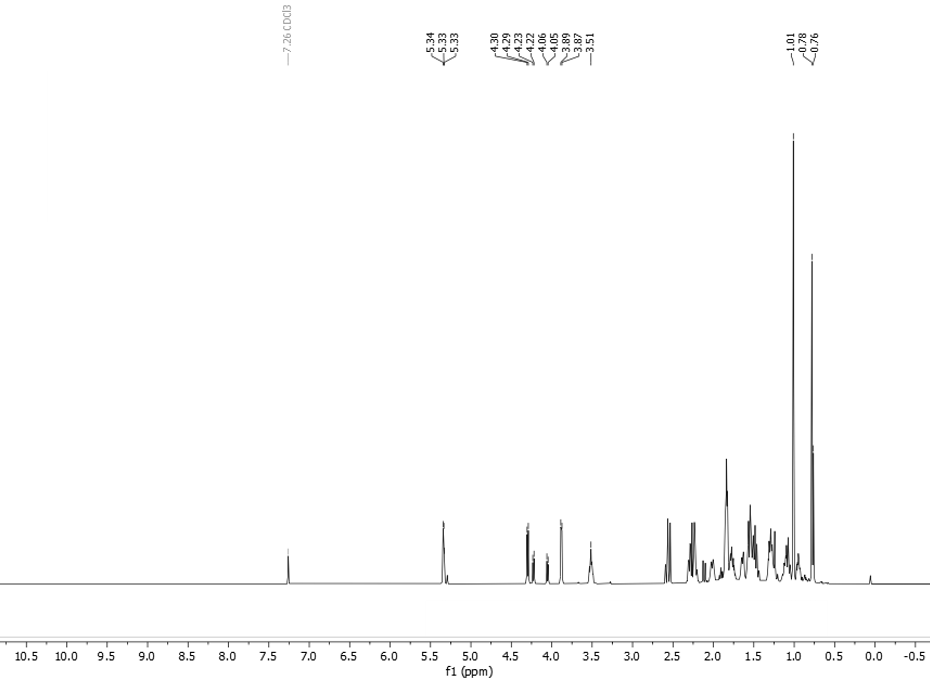


Supplementary Figure S77. ^1^H NMR spectrum (600 MHz, CDCl_3_) of ENT-A088.


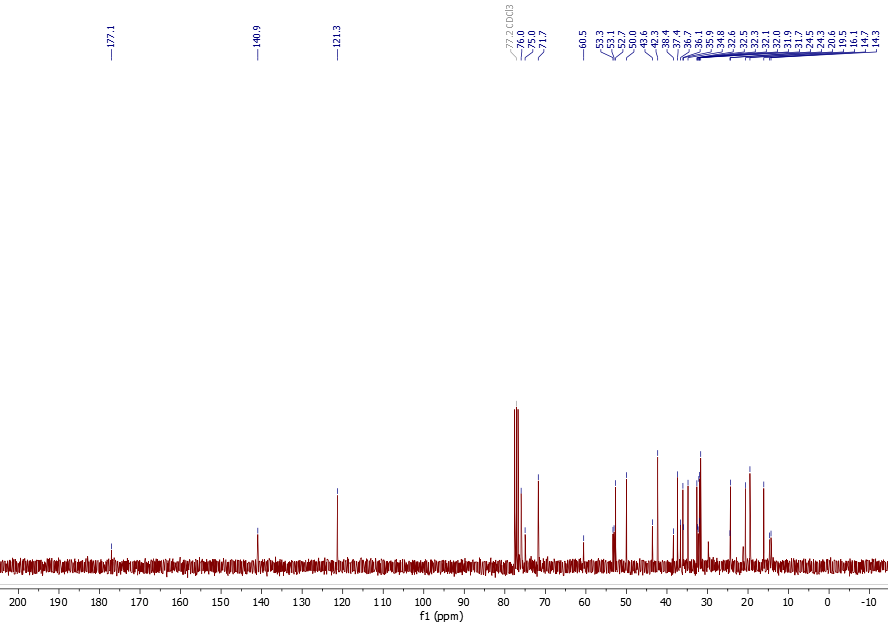


Supplementary Figure S78. ^13^C NMR spectrum (75 MHz, CDCl_3_) of ENT-A088.

## HLPC chromatograms

##
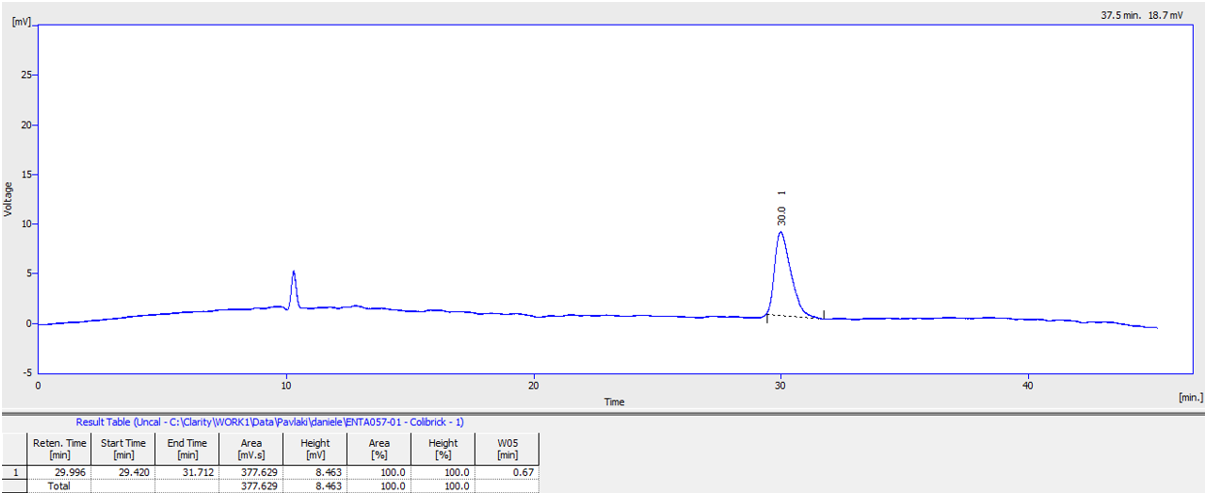


Supplementary Figure S79. HLPC chromatogram of ENT-A007.


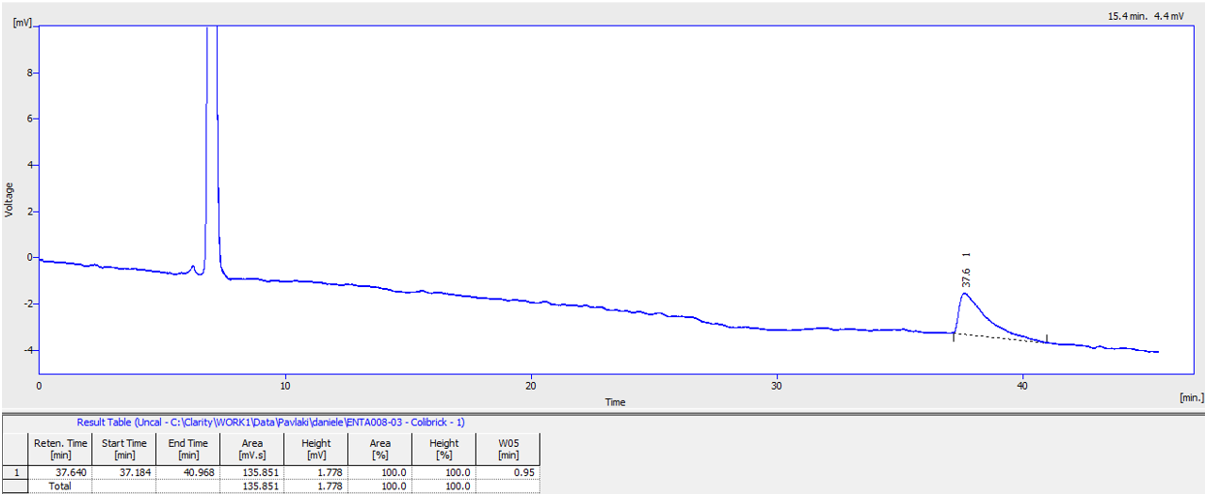


Supplementary Figure S80. HLPC chromatogram of ENT-A008.


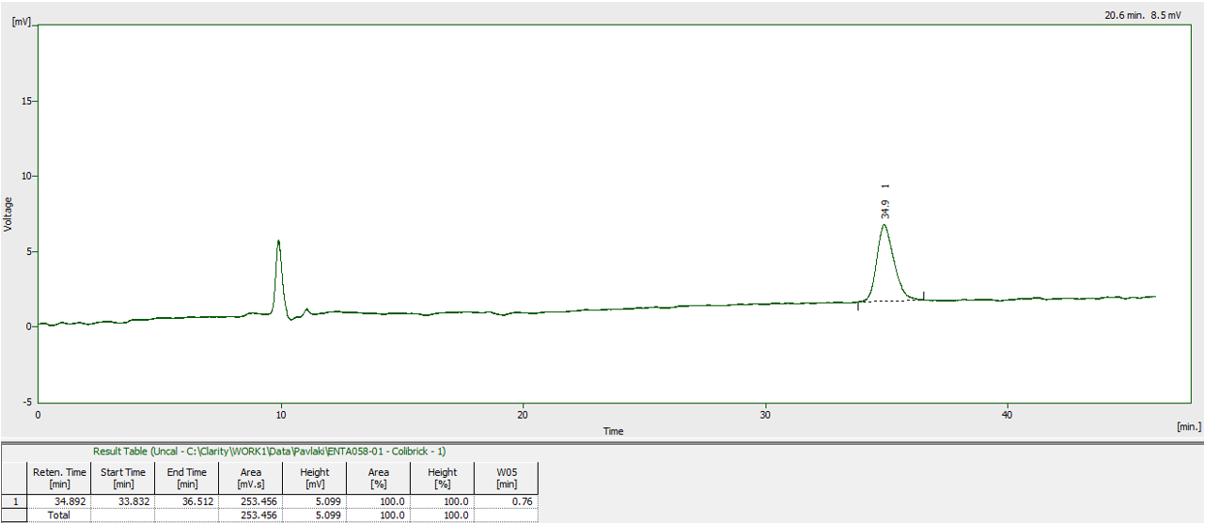


Supplementary Figure S81. HLPC chromatogram of ENT-A009.


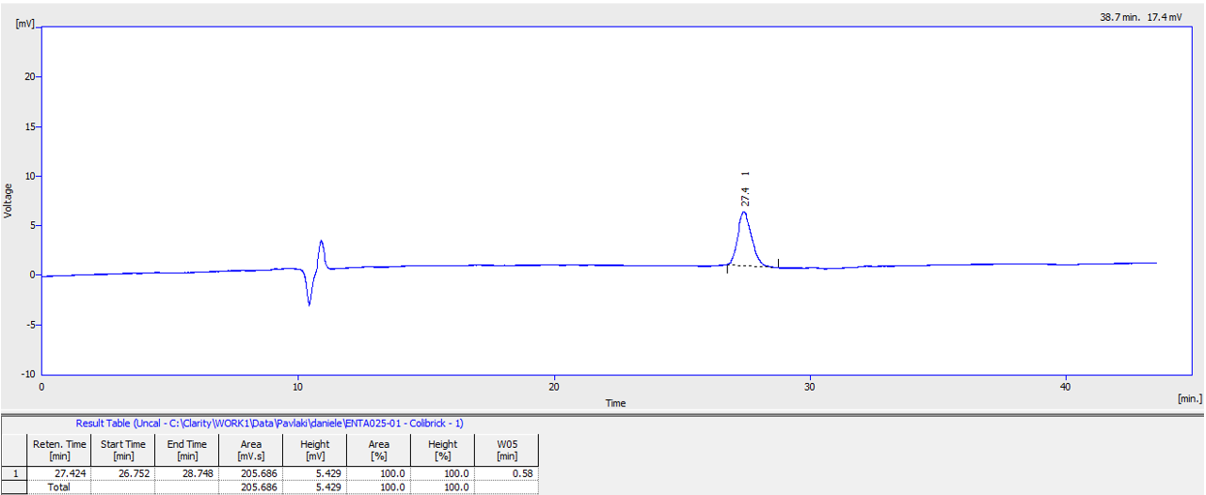


Supplementary Figure S82. HLPC chromatogram of ENT-A025.


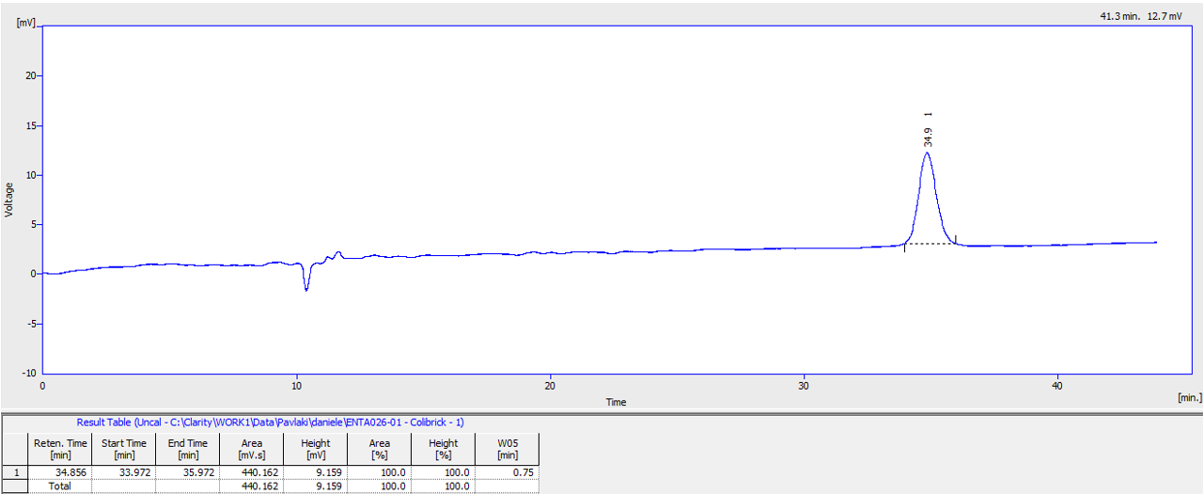


Supplementary Figure S83. HLPC chromatogram of ENT-A026.


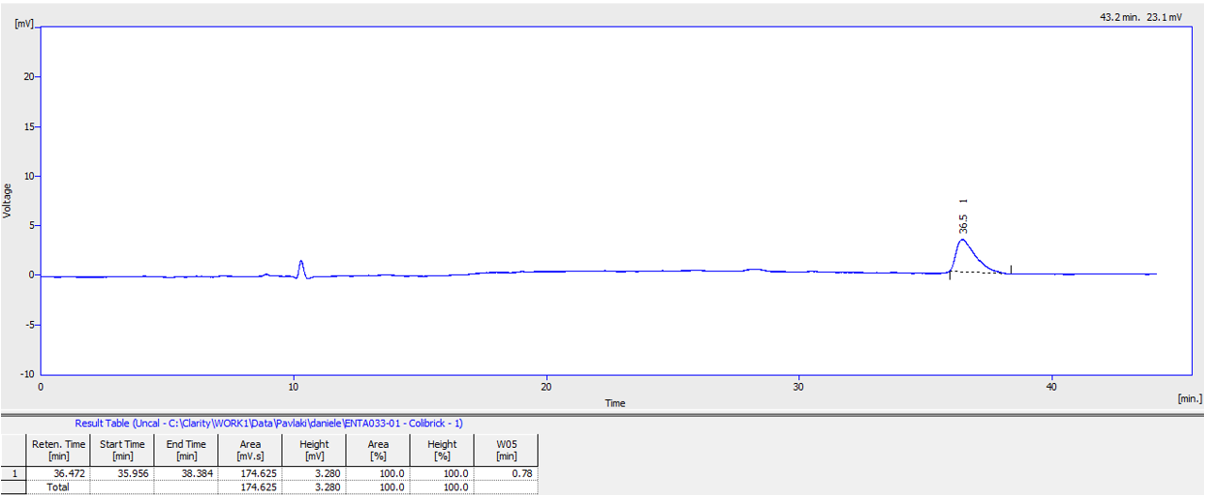


Supplementary Figure S84. HLPC chromatogram of ENT-A033.


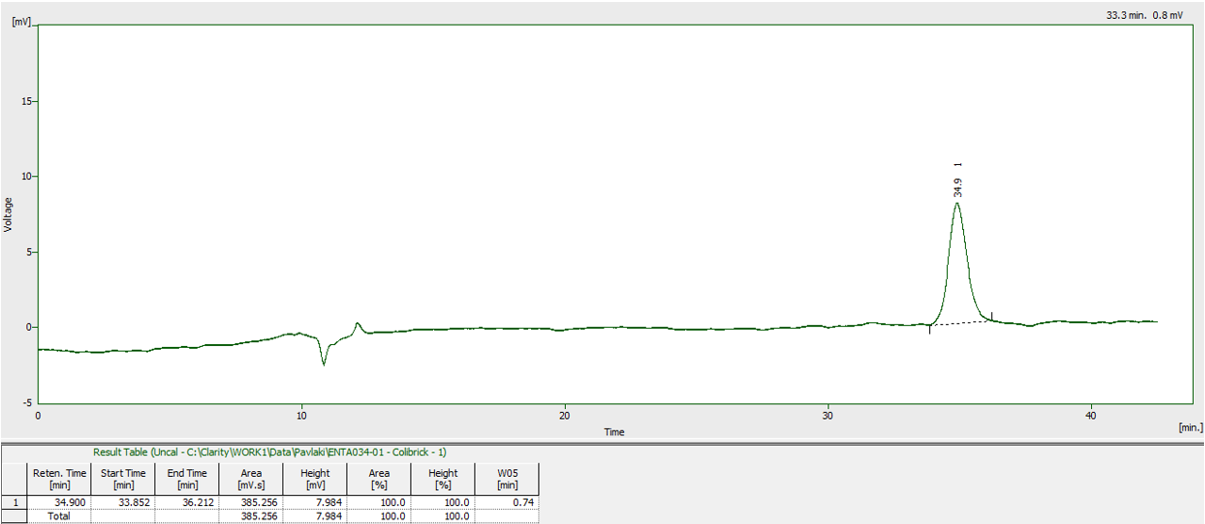


Supplementary Figure S85. HLPC chromatogram of ENT-A034.


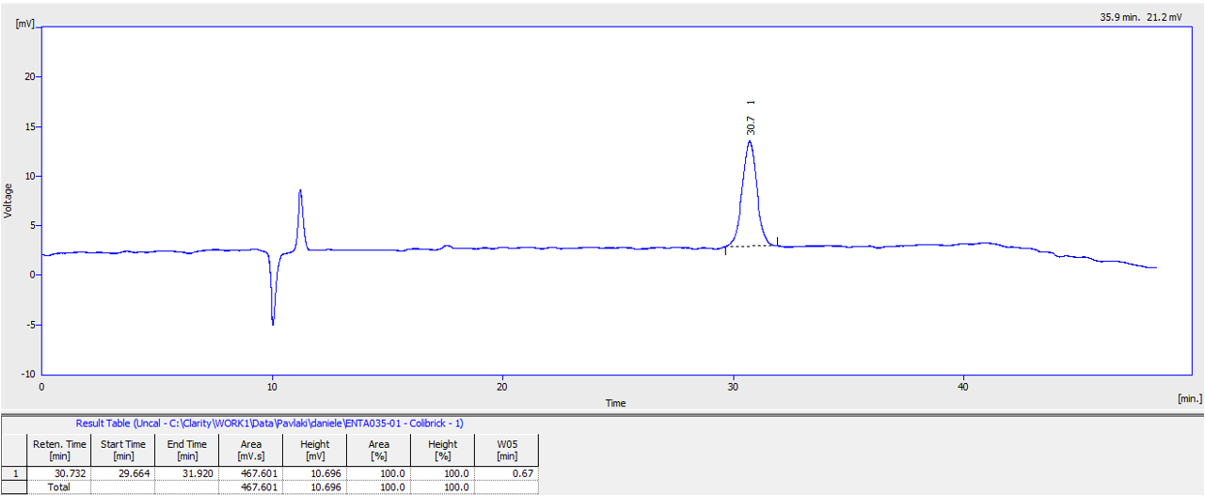


Supplementary Figure S86. HLPC chromatogram of ENT-A035.


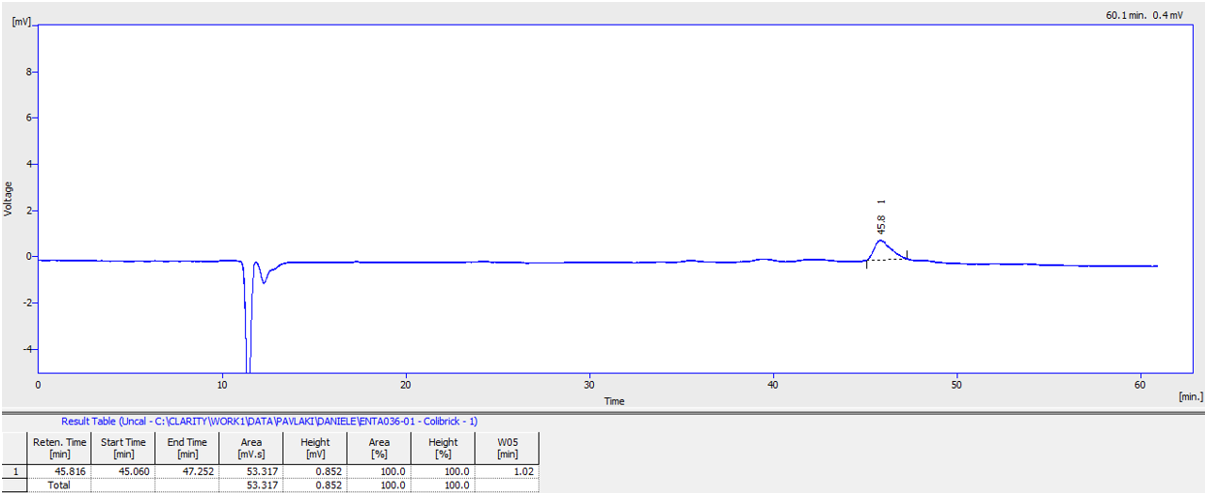


Supplementary Figure S87. HLPC chromatogram of ENT-A036.


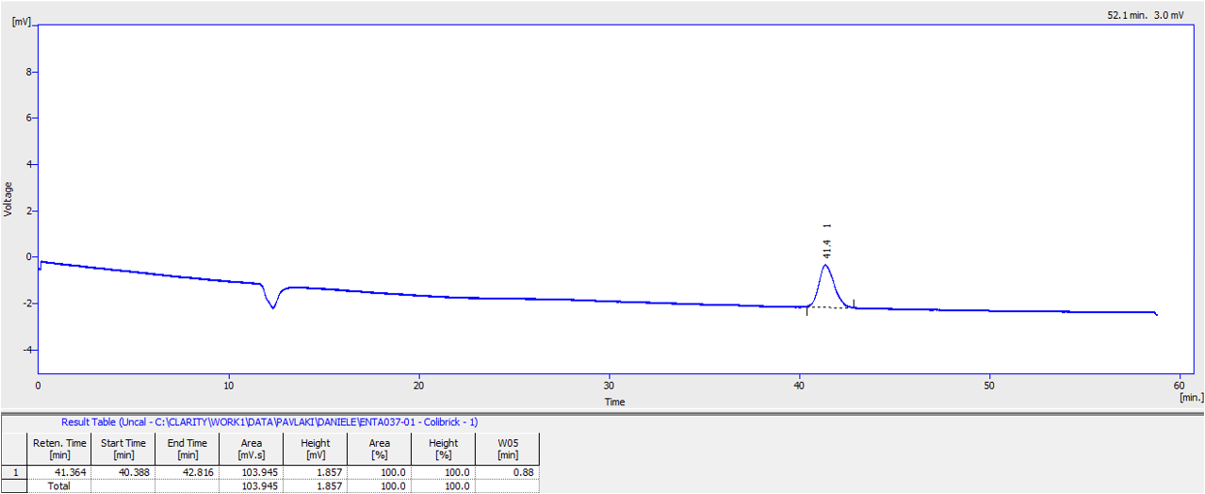


Supplementary Figure S88. HLPC chromatogram of ENT-A037.


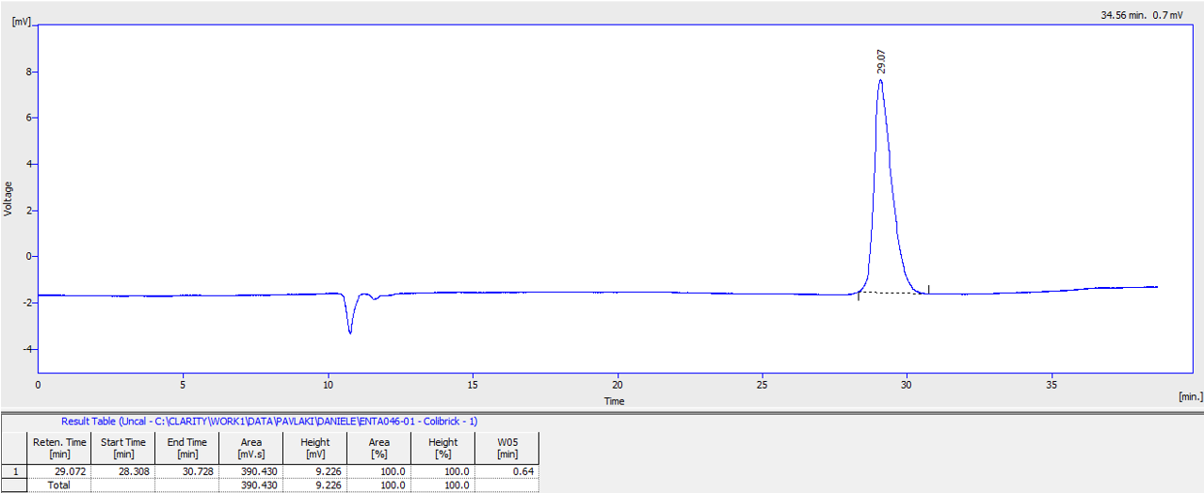


Supplementary Figure S89. HLPC chromatogram of ENT-A046.


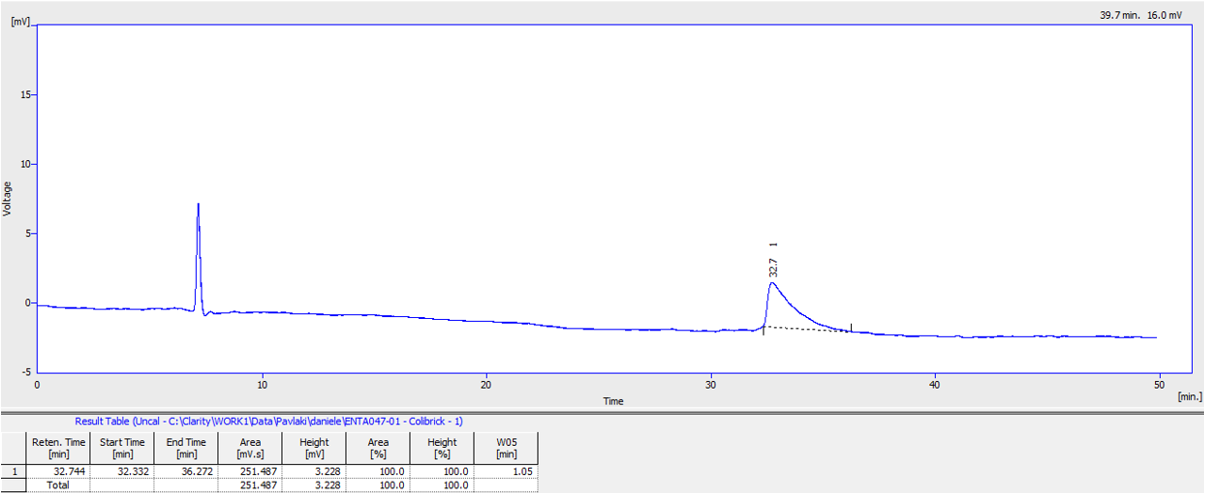


Supplementary Figure S90. HLPC chromatogram of ENT-A047.


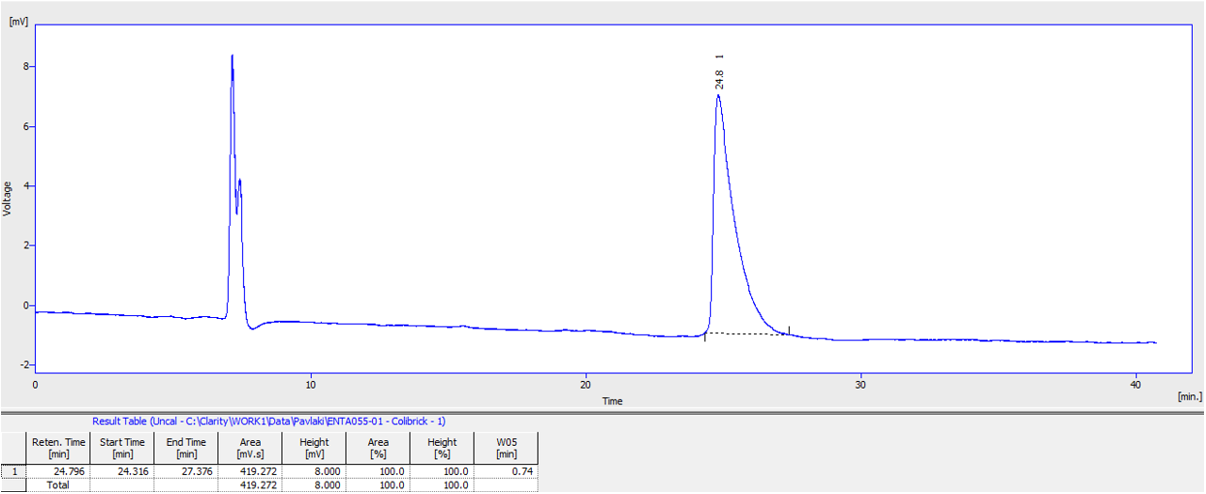


Supplementary Figure S91. HLPC chromatogram of ENT-A055.


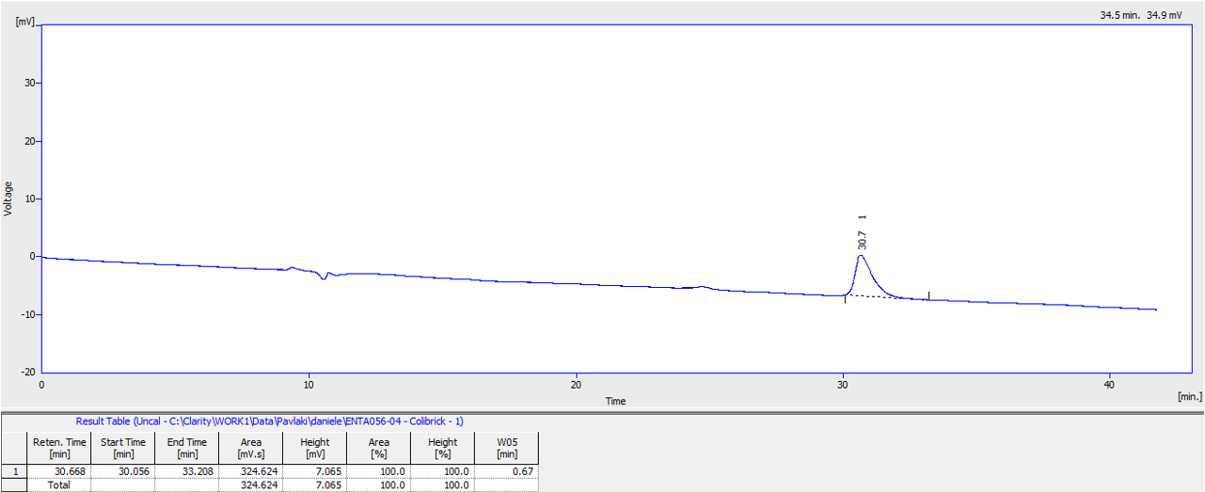


Supplementary Figure S92. HLPC chromatogram of ENT-A056.


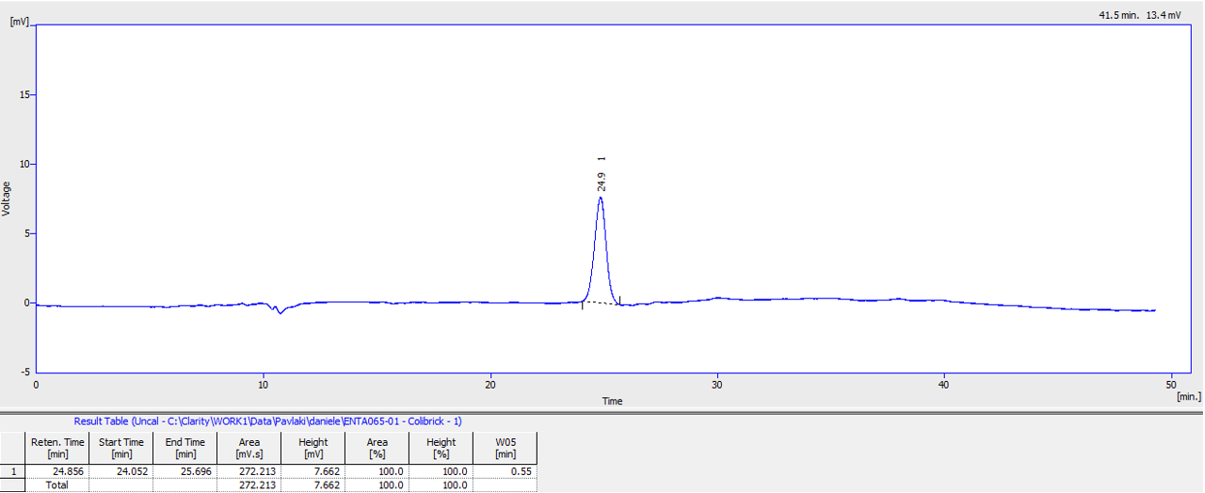


Supplementary Figure S93. HLPC chromatogram of ENT-A065.


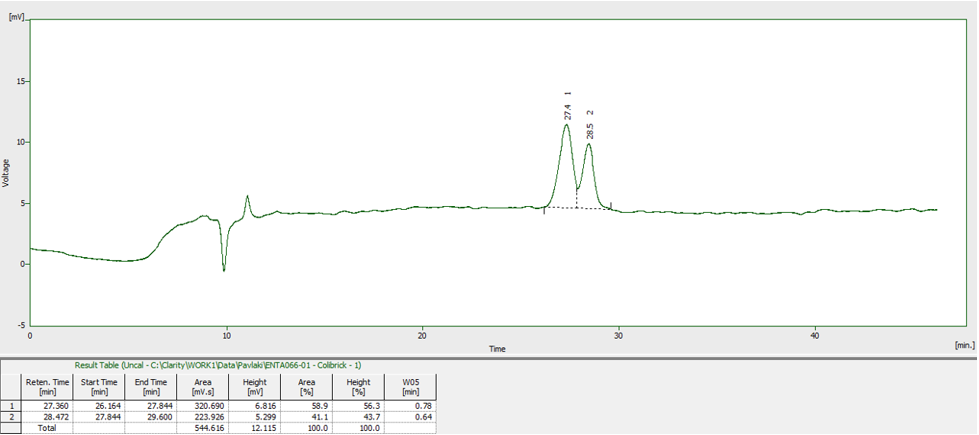


Supplementary Figure S94. HLPC chromatogram of ENT-A066.


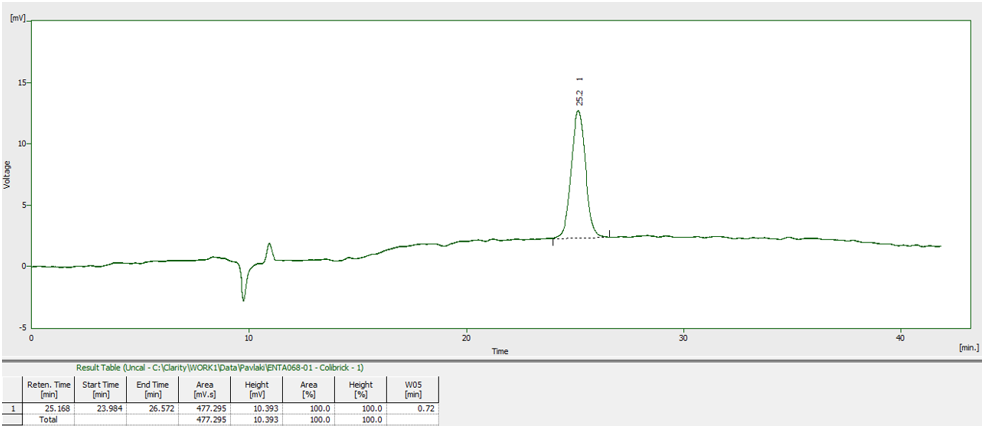


Supplementary Figure S95. HLPC chromatogram of ENT-A068.


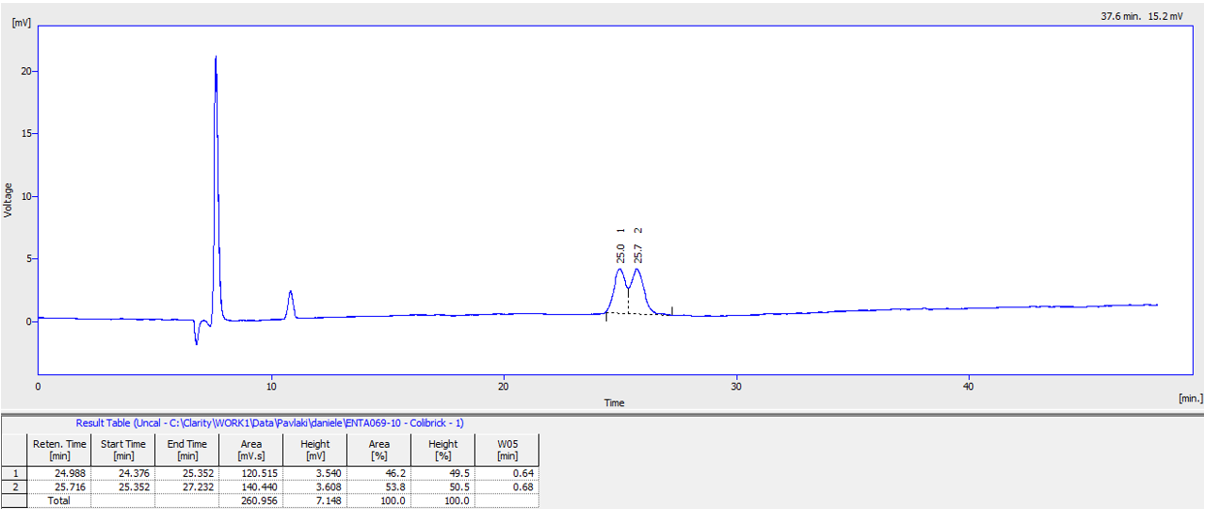


Supplementary Figure S96. HLPC chromatogram of ENT-A069.


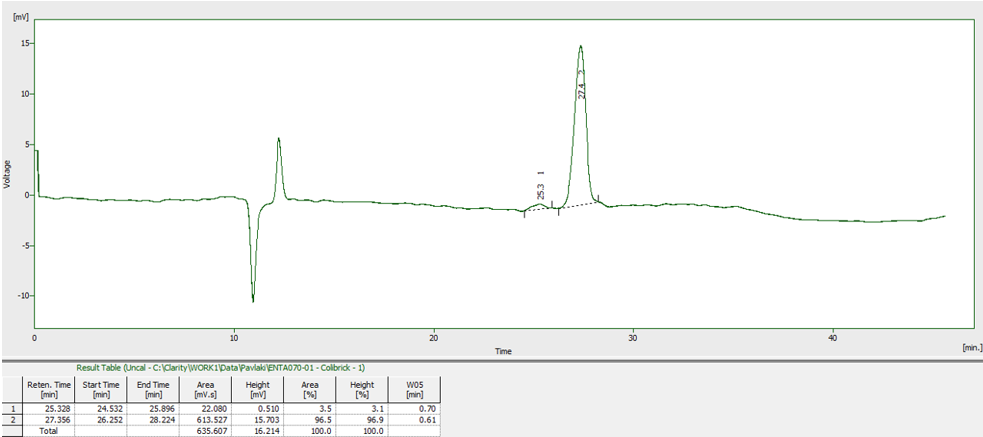


Supplementary Figure S97. HLPC chromatogram of ENT-A070.


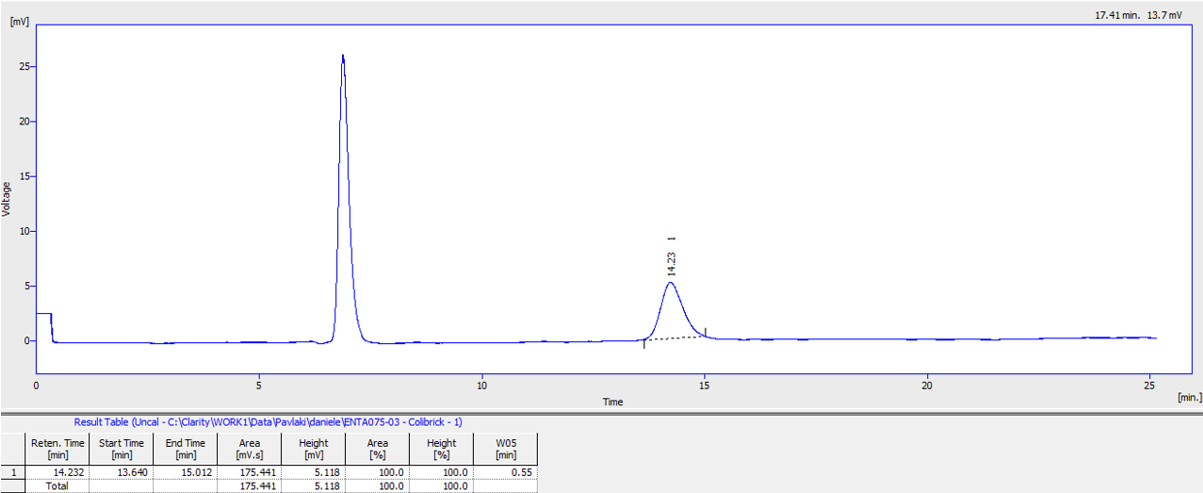


Supplementary Figure S98. HLPC chromatogram of ENT-A075.


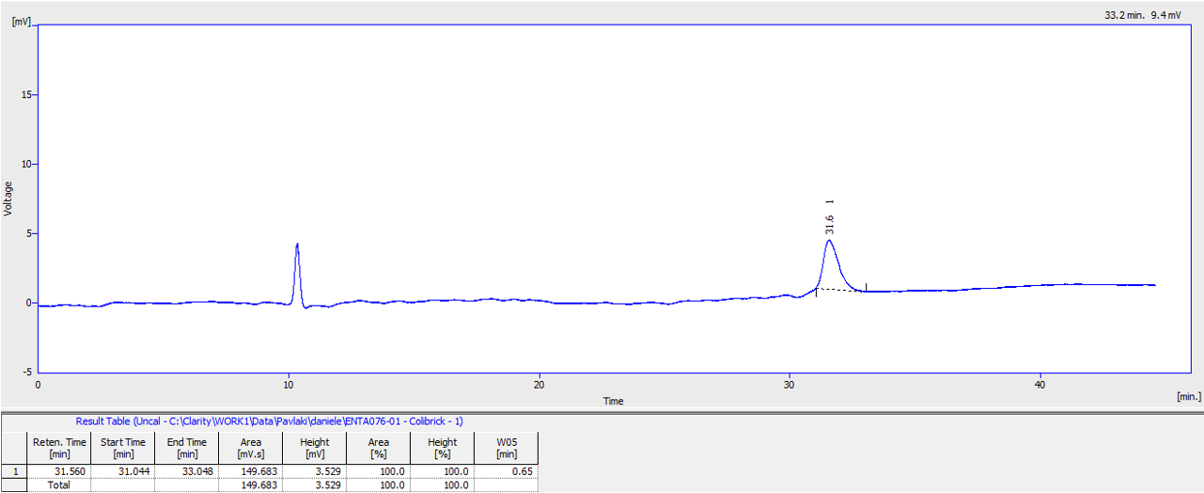


Supplementary Figure S99. HLPC chromatogram of ENT-A076.


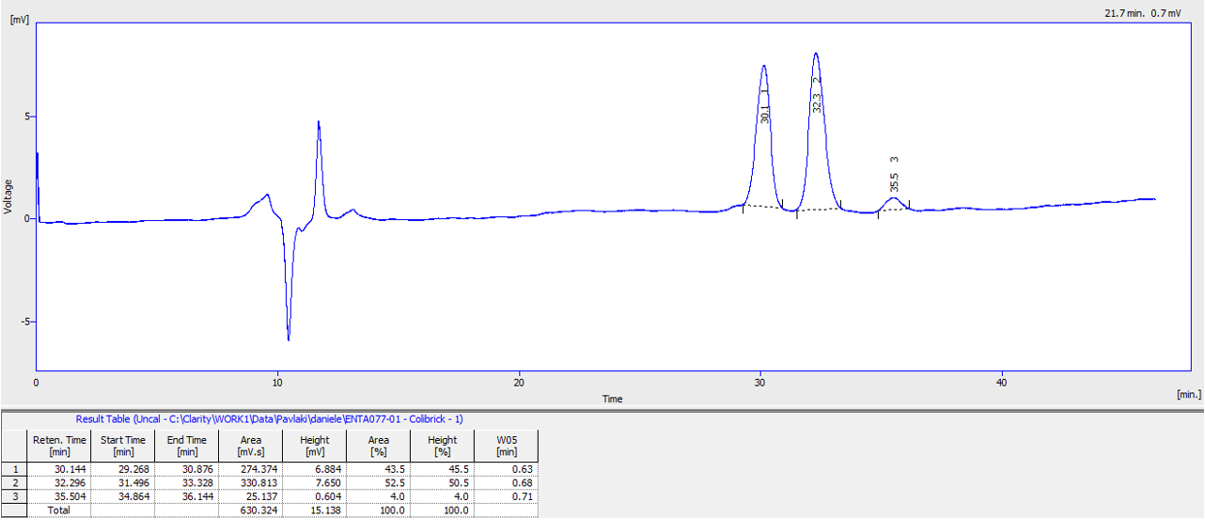


Supplementary Figure S100. HLPC chromatogram of ENT-A077.


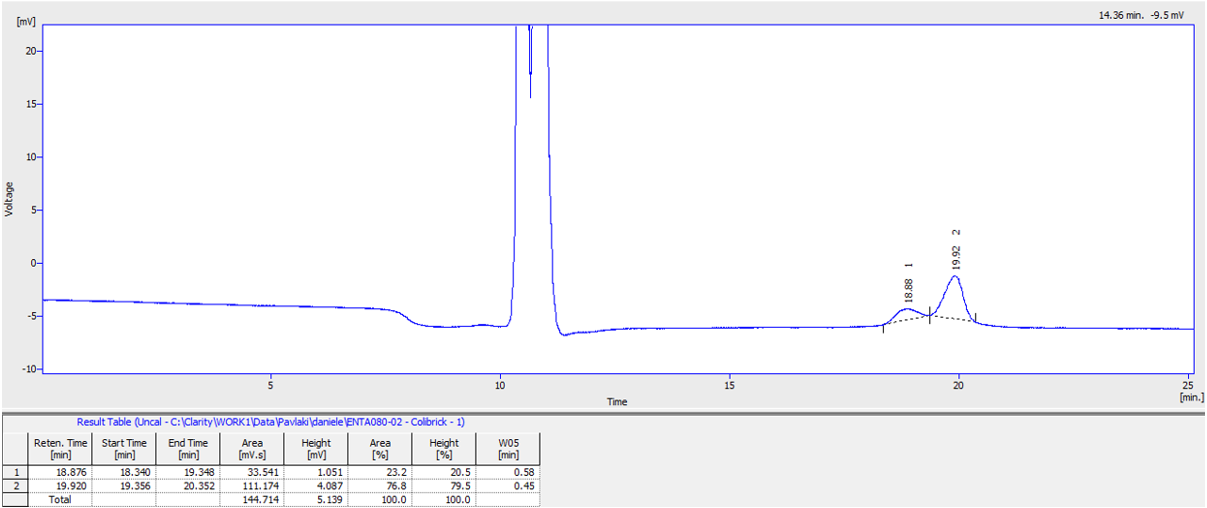


Supplementary Figure S101. HLPC chromatogram of ENT-A080.


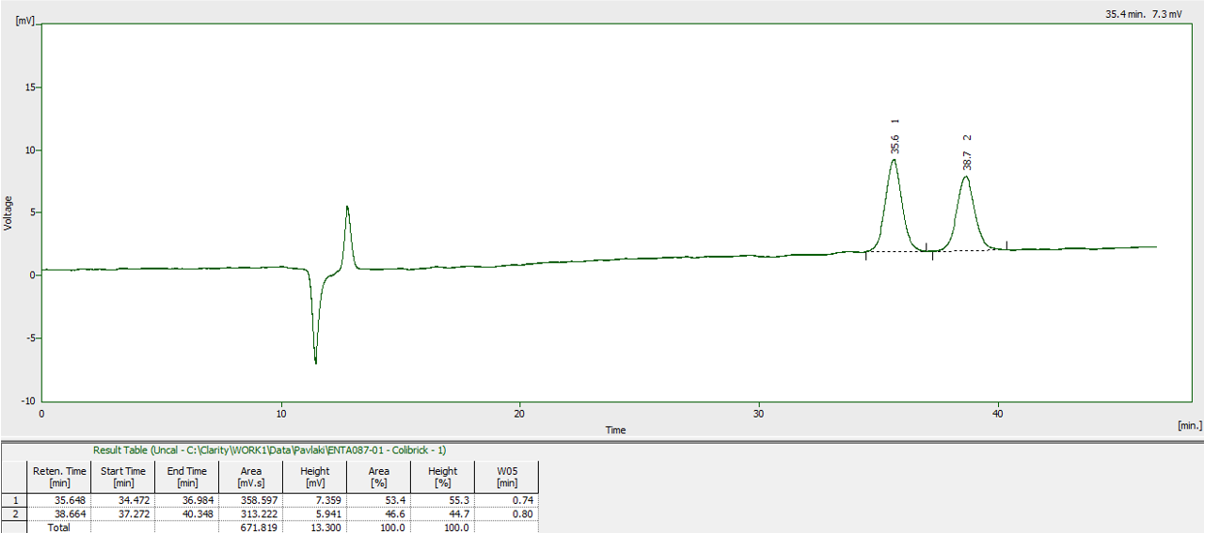


Supplementary Figure S102. HLPC chromatogram of ENT-A087.


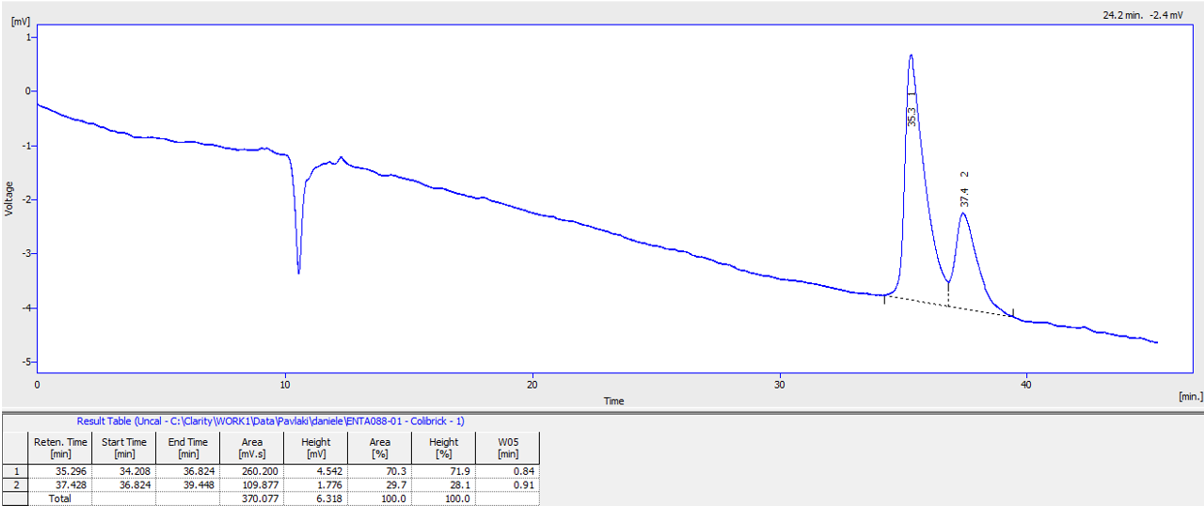


Supplementary Figure S103. HLPC chromatogram of ENT-A088.
